# Computational prediction of substrate scope of a homogeneous catalyst: the case of metal-free C–H borylation by a frustrated Lewis pair catalyst

**DOI:** 10.1039/d6sc04421b

**Published:** 2026-08-06

**Authors:** Jacob M. Nielsen, Maria H. Rasmussen, Jan H. Jensen

**Affiliations:** a Department of Chemistry, University of Copenhagen Denmark jhjensen@chem.ku.dk

## Abstract

Predicting substrate scope in catalytic reactions from first principles requires evaluating not only the lowest-energy productive pathway, but also competing processes that can suppress turnover. Here we present FRUST, an automated computational workflow for substrate-scope prediction, and apply it to metal-free C–H borylation mediated by the boron-based frustrated Lewis pair catalyst developed by Fontaine and co-workers (*Science* 2015, **349**, 513–516). FRUST generates intermediates and transition-state guesses for each unique aromatic C–H position, performs conformational sampling, and estimates barriers using constrained xTB geometries refined by DFT single-point calculations. Benchmarking against literature substrates shows that the workflow captures the dominant mechanistic trends of the FLP catalytic cycle and identifies TS1 as the principal barrier-defining step across both benchmark and screening sets. Application to 69 aromatic substrates identified a small number of low-barrier candidates, including phenols, *N*-aryl amides, and selected aprotic arenes. However, targeted DFT validation showed that even the most promising aprotic candidates lie near or beyond the empirical reactivity window, suggesting that the current FLP catalyst is unlikely to exhibit broad substrate scope beyond known activated heteroarenes. Analysis of protic substrates further revealed important off-cycle limitations: O–H borylation of phenols is predicted to deactivate neighbouring C–H sites, while *N*-aryl amides are likely to undergo N–H borylation without subsequent productive C–H borylation. These results demonstrate that efficient computational screening can retain mechanistic resolution, but also highlight the need to include side-reaction prediction when assessing catalyst scope and designing next-generation metal-free borylation catalysts.

## Abstract 2

Predicting substrate scope in catalytic reactions from first principles requires evaluating not only the lowest-energy productive pathway, but also competing processes that can suppress turnover.

## Graphical abstract

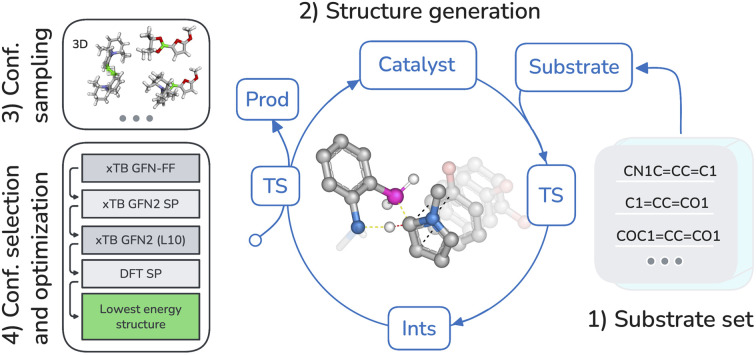

## 1 Introduction

While “thousands of reports of synthetic methodology get published each year… the vast majority of the reactions never find their way into industrial application”.^1^ One reason is that the substrate scope has been shown to be small and another is that the substrate scope has not been fully delineated (due to time and expense constraints) and is therefore largely unknown. Few chemists are then willing to take the chance on such new reactions.

Take, for example, the boron-based frustrated Lewis pair (FLP) catalyst for C–H borylation developed by Fontaine and co-workers.^2,3^ While there are several transition-metal (TM) based catalysts for C–H borylation, metal-free alternatives are highly sought after since TMs can be expensive, toxic, and air-sensitive.^4^ However, Fontaine's metal-free alternative has been shown to work only on a relatively limited number of activated C–H sites and appears to be inactivated by O–H and N–H groups in the substrate through side reactions.^2,5^

In principle, substrate scope can be predicted computationally but it is very difficult to do in practice, because the barrier heights for all steps in the catalytic cycle must be computed for each substrate to check whether the barrier to the rate determining step is sufficiently low to allow for reasonable reaction rates. Computing barriers involves the determination of transition states (TSs) which is a difficult process to automate and tends to be time-consuming. While many algorithms have been proposed to automate transition-state searches—from classical eigenvector-following and rational-function optimisation to dimer/min-mode-following, chain-of-states methods such as NEB and string/growing-string/freezing-string variants, activation–relaxation techniques, and, more recently, machine-learning-assisted optimisers,^6–33^ in practice the failure rate is relatively large and the computational cost can be quite high compared to finding intermediates.

To our knowledge, the only computational study that has predicted substrate scope for an appreciable number of substrates is the study by Sawatlon *et al.*,^34^ who considered 81 different substrates for the Suzuki-coupling reactions. The TSs determination appears to have been done manually and side reactions, which can also limit substrate scope, were not considered.

Here we introduce a computational method for predicting substrate scope and apply it to Fontaine's FLP catalyst. The core of the method is based on a TS-template approach,^11,35–39^ which we^38^ previously used to screen catalysts. Applied to 69 Ir-compatible aromatic substrates, the workflow identifies a small set of apparent low-barrier candidates. Subsequent targeted DFT calculations and side-reaction analysis show that several of these candidates are limited by catalyst deactivation or competing heteroatom borylation, highlighting the need to combine productive-cycle screening with analysis of mechanistic failure modes. The methodological advance in FRUST is not the introduction of a new TS search algorithm, but the use of constrained transition-state templates as calibrated energetic surrogates for all elementary steps and all unique aromatic C–H positions in a catalytic cycle. This makes it possible to screen substrate scope at the level of rate-determining barrier estimates before committing selected cases to full DFT transition-state optimization. FRUST therefore complements general automated reaction-discovery and transition-state workflows by addressing a different task: prospective, mechanism-resolved substrate-scope triage for a defined catalytic manifold.

By combining automated barrier estimation with targeted analysis of competing pathways, this work provides a practical test of whether Fontaine's FLP catalyst can be extended beyond its currently known substrate scope. Rather than identifying a broad set of immediately viable new substrates, the calculations show that most Ir-compatible arenes remain kinetically inaccessible with the present FLP catalyst. The few substrates that appear promising in the automated screen require further scrutiny, as more detailed DFT calculations and analysis of heteroatom borylation reveal competing pathways that can prevent productive C–H borylation. Thus, the main outcome is a mechanistic map of the limitations of the current catalyst, together with a workflow for distinguishing genuine substrate-scope opportunities from apparent low-barrier cases that are undermined by side reactions.

## 2 Methods

### 2.1 General considerations

The goal is to predict whether a substrate is likely to be converted to the desired product with an appreciable yield, in a reasonable amount of time, under reasonable reaction conditions. In order to be a viable substrate all of the following conditions have to be met:

(1) The standard free energy of the product must be lower than the standard free energies of the reactants and all intermediates, *i.e.* the thermodynamic sink should be the product. This assumes that the standard free energies have been corrected to reflect any effective changes in standard state due to, for example, large excess of one or more reactants, or the continuous removal of one or more products to drive the reaction forward. For example, the reaction mechanism studied in this paper involves the production of H_2_ gas. Since most of the experiments are done in a sealed container it is appropriate to use the 1 bar standard state for H_2_ since the partial pressure of H_2_ will reach an equilibrium value that depends on the extent of the reaction. However, if the experiments were done in an open container where H_2_ can escape, the partial pressure of H_2_ will equilibrate to a constant value (the partial pressure of H_2_ in the atmosphere) so that would be the correct standard state.

(2) All standard free energy barriers computed relative to the reactant in the main catalytic cycle that converts the substrate to the desired product must be below a certain threshold. This assumes that there is no thermodynamic sink preceding that barrier. This threshold is usually around 25 to 30 kcal mol^−1^ depending on the temperature the reaction is run at. For example, a first order reaction with a half-life of 6 hours (*i.e.* 50% yield) will have barriers (standard activation free energies) of 23.6 and 28.1 kcal mol^−1^ at 25 °C and 80 °C, respectively following the Eyring equation. These values will be slightly different for higher order reactions (where the half-life also depends on initial concentration), but given exponential dependence of the rate constant on the barrier height and the inherent errors in computed barriers, a value between 25 to 30 kcal mol^−1^ is often used as a cutoff.

(3) Any reaction path that does not lead to the desired product must have at least one barrier that is at least 2–3 kcal mol^−1^ higher than the rate determining step (or state – RDS^40^) in the catalytic cycle. This again assumes that there is no thermodynamic sink preceding that barrier.

The fact that all criteria have to be met can be used to increase the efficiency of the screen. For example, if the standard free energy of the product is significantly higher than that of the reactants, one can categorize the substrate as out-of-scope without doing any additional calculations. Similarly, if the first barrier in the catalytic cycle is above the threshold one does not need to compute the barriers for the subsequent steps.

### 2.2 Computational methodology

**Fig. 1:**
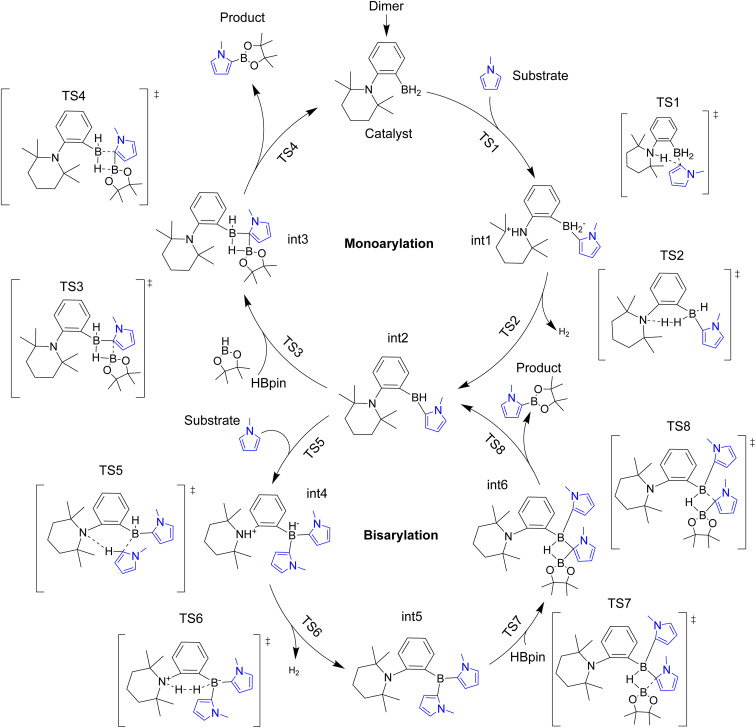
Catalytic manifold for FLP-mediated C–H borylation, including the productive monoborylation cycle and the competing bis-arylation side reaction. The amine-borane dimer first dissociates to the catalytically active monomer. In the productive cycle, the catalyst reacts with the substrate through TS1 to form the zwitterionic intermediate int1, followed by H_2_ release through TS2 to give int2. Reaction of int2 with pinacolborane (HBpin) then proceeds through int3 and TS3/TS4 to furnish the borylated product and regenerate the catalyst. In the competing bis-arylation branch, int2 is intercepted by a second substrate equivalent and proceeds through int4–int6 and TS5–TS8 to form a bis-arylated off-cycle product. All intermediates and transition states considered in the present study are shown explicitly. The substrate shown (in blue) is 1-methylpyrrole, as used in the original study by Légaré *et al.*^2^

**Fig. 2:**
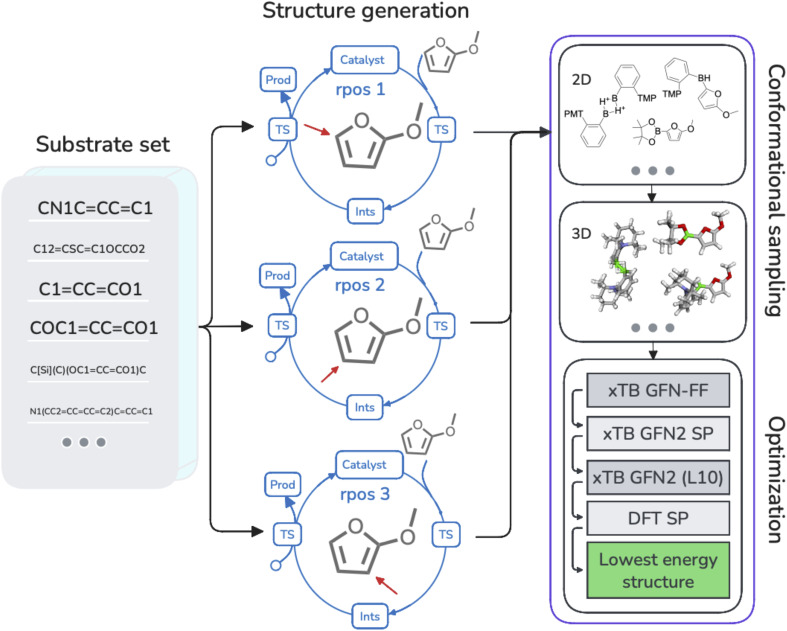
The FRUST workflow. Substrate set: the process starts with a list of SMILES for substrates of interest. Structure generation: for each substrate, the complete set of structures that make up the catalytic cycle is generated. This is then repeated for every unique aromatic C–H bond in the substrate; we refer to these sites as reactive positions (rpos) (*e.g.* rpos 1, rpos 2, …). Conformational sampling: for each structure, a conformational ensemble is generated. Optimization: the entire ensemble is optimized using the semi-empirical Extended Tight Binding force field (xTBFF)^41^ method, followed by GFN2-xTB^42^ single-point (SP) calculations. The 10 lowest-energy (L10) conformers are re-optimized at the GFN2-xTB level, and finally density functional theory (DFT) single-point calculations are performed on the constrained optimized xTB structures; the lowest-energy structure is retained for analysis.

To estimate reaction barriers and intermediate energies, we generate transition state guess structures (TSGs) and intermediate structures for each step of the FLP catalytic cycle (see Fig. 1), illustrated here using 1-methylpyrrole (blue) as a representative substrate. The overall workflow, termed FRUST (see Fig. 2), is designed to accommodate arbitrary aromatic substrates. The procedure consists of four main stages. First, a list of SMILES strings representing the substrates to be examined is provided as input. Second (structure generation), intermediate structures and TSGs are constructed for every unique C–H position of each substrate (we refer to these as rpos *i.e.* reactive position(s)). For example, for 2-methoxyfuran (shown in Fig. 2), three distinct reactive C–H positions are identified, resulting in three sets of intermediate 1, three sets of TS1 guesses and so on being generated. In effect, the entire catalytic cycle is instantiated separately for each rpos. Note here that TSGs are created using a template-based transfer protocol explained below. In the third step, conformational sampling is performed for all generated structures. Finally, intermediates are subjected to unconstrained geometry optimizations at the xTB level of theory, whereas TSGs are refined under selected geometric constraints to preserve the intended reaction-coordinate geometry and avoid collapse to adjacent minima.

#### 2.2.1 Structure generation

Structure generation proceeds *via* two complementary routes: one for the TSGs, and one for the intermediates.

##### 2.2.1.1 TSGs

**Fig. 3:**
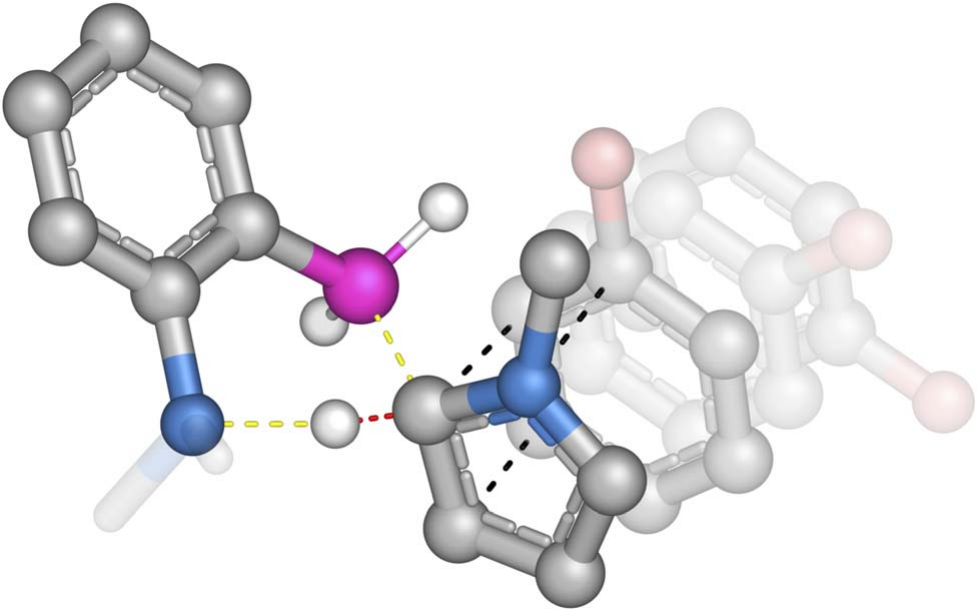
The reference transition state (TS) geometry with transfer substrate: the catalyst and the substrate in the reference structure (1-methylpyrrole) is here shown for TS1. The atom colors are blue (nitrogen), purple (boron), red (oxygen), grey (carbon) and hydrogen (white). The yellow dashed bonds shows where bonds are being formed and the red shows the bond to hydrogen being broken on the reference substrate. Finally, the superimposition of a transfer substrate (phenol, transparent structure) onto the reference substrate is shown. The black dashed lines show the three atom pairs that are being used for alignment. Indices are selected such that the rpos will align with the C–H site to borylate. Note that the (TMP, 2,2,6,6-tetramethylpiperidine) substructure on the catalyst has been partly hidden for better viewing and only relevant hydrogens are shown.

TSGs are generated using a template-based transfer protocol starting from a pre-optimized reference TS geometry (Fig. 3). This structure is obtained from Légaré *et al.*^2^ The reference structure contains a prototypical substrate (1-methylpyrrole) bound in the active site. We then identify the atoms to be constrained (shown in Fig. S1 and S2) and then use RDKit's rdDistGeom.EmbedMultipleConfs to generate *n* conformations for the remaining atoms, where *n* is 50, 200, or 300 conformers generated for molecules with ≤7, 8–12, or >12 rotatable bonds, respectively.^43^ The resulting ensembles of 3D conformers are optimized with GFN-FF and GFN2-xTB single-point (SP) energies are obtained on these structures. During all steps, *i.e.* sampling with ETKDG and subsequent xTB optimizations the geometric constraints were set in the respective stages. The 10 lowest-energy structures are retained and re-optimized at the GFN2-xTB level (again with relevant constraints), followed by DFT single-point calculations using the ωB97X-D functional^44^ and the 6-31G** basis set. For each species, the electronic energy, Δ*E*, at the ωB97X-D/6-31G** level of theory on constrained GFN2-xTB structures is used for screening.

Δ*E*^‡^_DFT//xTB_ values obtained on TSGs are corrected using the linear relationship derived from the benchmarking study (see Benchmarking the barrier estimation). For example, for TS1 we obtain Δ*G*^‡,cal^_DFT_ = 0.92Δ*E*^‡^_DFT//xTB_ + 2.97. Thus, a value of Δ*E*^‡^_DFT//xTB_ = 20 kcal mol^−1^ is mapped to Δ*G*^‡,cal^_DFT_ = 0.92 × 20 kcal mol^−1^ + 2.97 = 21.37 kcal mol^−1^.

##### 2.2.1.2 TSs

For benchmarking and validation, the optimized TSGs are re-optimized under geometric constraints at the ωB97X-D/6-31G** level, followed by an unconstrained TS optimization at the same level. TSs are verified by one imaginary frequency. We discard structures that have multiple or no imaginary frequencies, or ones that are very small compared to the average of a particular TS class. A final SP refinement in chloroform (SMD) is performed using ωB97X-D/6-31+G** with gibbs energy correction at the ωB97X-D/6-31G** level.

##### 2.2.1.3 Intermediates

Intermediates and products are generated by identifying unique C–H position on the substrate and relevant bonds are created using RDKit. The only exception is intermediate 3. This one is generated analogously to the transition states where a reference geometry from Légaré *et al.*^3^ is used. The substrate is grafted onto the reference structure and a constrained core is created by locking bond and angle distances. This is done because RDKit's ETKDG cannot generate chemically sound structures for this complex due to intermediate 3's 3-center-2-electron bonding pattern (see Fig. 1).

Conformational sampling and optimizations for intermediates follow the same procedure as for TSs (Fig. 2, conformational sampling and optimization). The only difference is that no constrained core is defined, except for intermediate 3 which follows the exact same procedure.

#### 2.2.2 Thermodynamics

All thermodynamic corrections are computed at 25 °C and 1 M standard state (except H_2_ which is computed at 1 bar). The free energy terms are computed using either ORCA (v. 6.1.0)^45^ or xTB (v. 6.7.1).^46^ While the experiments usually are done at higher temperatures, the main focus here is the difference in free energy changes relative to those of known substrates where the effect of temperature and other parameters will tend to cancel.

## 3 Results and discussion

### 3.1 Benchmarking the barrier estimation

To assess the accuracy of xTB-derived TSG geometries for predicting DFT energetics, we compare DFT-SP electronic energies computed on constrained xTB-optimized TSGs with reference barriers obtained from DFT-optimized TS structures. A set of 11 substrates, comprising 32 unique C–H positions in total, was curated from earlier studies on FLP borylation chemistry;^2,3^ thus, selection was based on the availability of experimental evidence. The set predominantly consists of pyrrole- and furan-derived heteroaromatic systems (Table S1). This composition reflects the available experimental and computational literature for FLP-mediated C–H borylation and therefore defines the principal applicability domain of the calibrated model. Predictions for substrates outside this domain, especially six-membered arenes and substrates bearing protic heteroatoms, are treated as screening hypotheses rather than final reactivity assignments and are subjected to targeted DFT validation below.

**Fig. 4:**
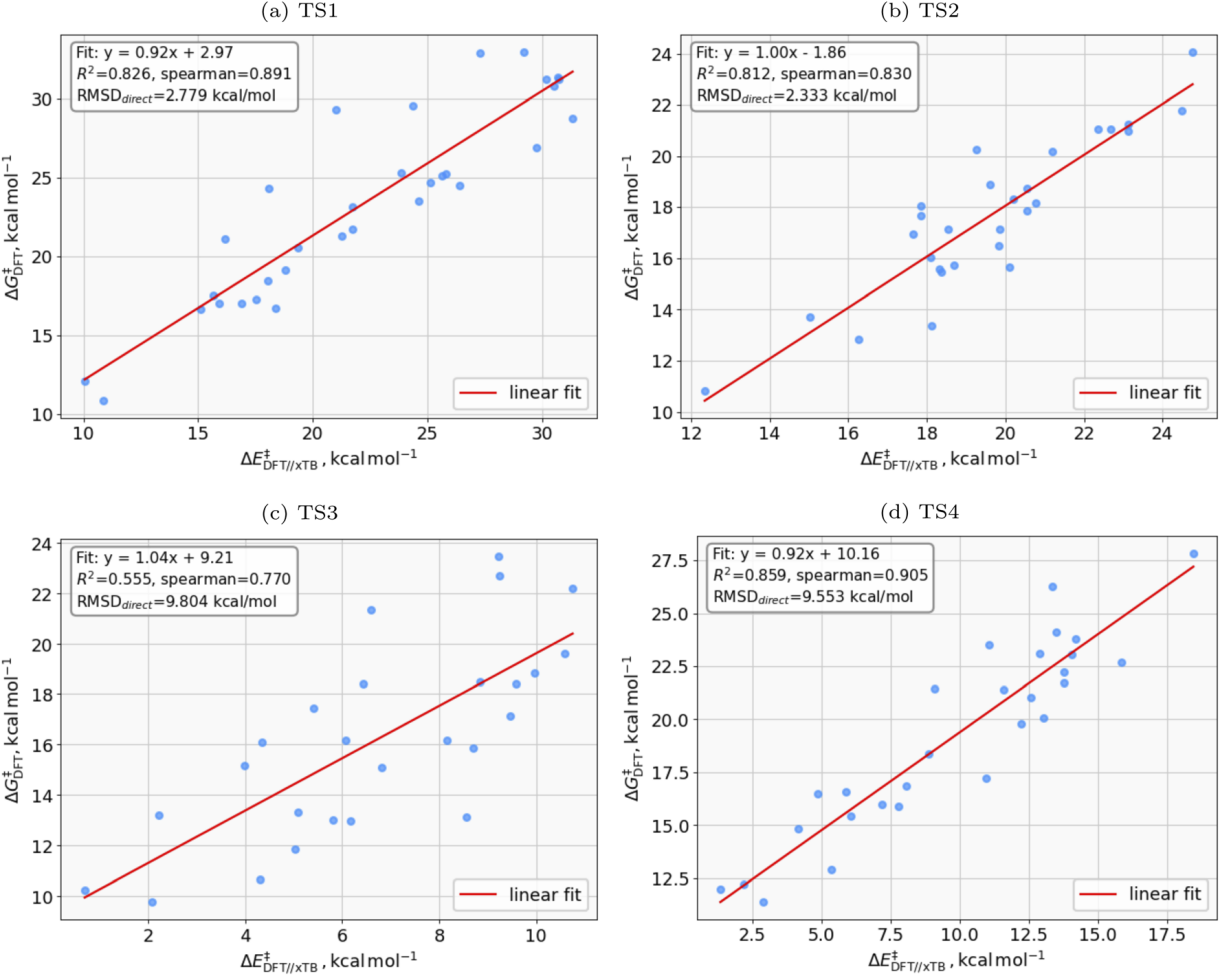
Correlation between DFT and xTB-derived energetics for TS1–TS4. The *y*-axis shows Gibbs free activation energies (Δ*G*^‡^_DFT_) obtained from DFT-optimized structures at the ωB97X-D/6-31G** level of theory corrected with a SP in chloroform SMD (ωB97X-D/6-31+G**). The *x*-axis shows DFT single-point electronic energies (Δ*E*^‡^_DFT//xTB_) computed at the same level on GFN2-xTB-optimized geometries. The red line represents linear regression. RMSD values displayed on the graph are from the unscaled correlation. The panels correspond to (a) TS1, (b) TS2, (c) TS3 and (d) TS4.

The linear relationship between the two approaches for TS1–TS4 is shown in Fig. 4.

After scaling, we observe good correlation for TS1, TS2, and TS4, each yielding *R*^2^ ≈ 0.8 with RMSD values of 2.5, 1.3, and 1.6 kcal mol^−1^, respectively. In contrast, TS3 shows a noticeably weaker correlation, with *R*^2^ = 0.56 and an RMSD of 2.5 kcal mol^−1^ (Fig. 4). One possible explanation is that the potential energy surface associated with TS3 is less well defined. The corresponding transition states exhibit relatively small imaginary frequencies, none larger than 180*i* cm^−1^. Smaller imaginary frequencies are consistent with a flatter potential energy surface and therefore greater variation in TS geometry. The other transition states display substantially larger imaginary frequencies; for example, TS1 shows values around 1000*i* cm^−1^ (see Tables S2–S5).

**Fig. 5:**
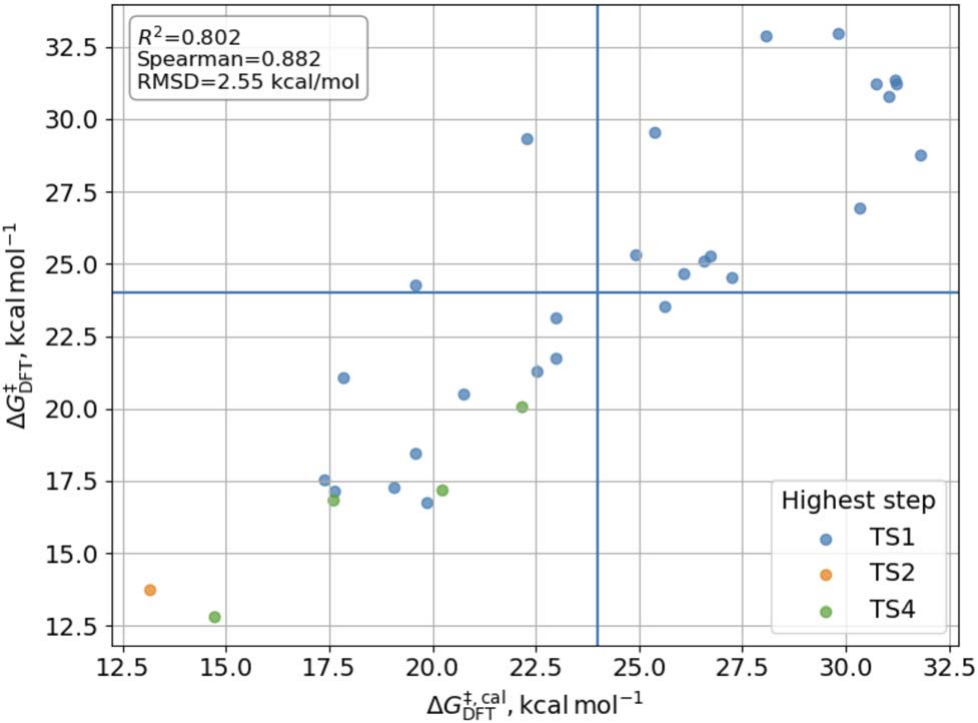
Comparison of DFT reference RDS barriers (Δ*G*^‡^_DFT_) and calibrated barrier estimates (Δ*G*^‡,cal^_DFT_) for the benchmarking set. Points are colored according to which transition state defines the DFT reference RDS. The horizontal and vertical lines indicate a 24 kcal mol^−1^ cutoff used to distinguish substrates expected to be viable under the reported reaction conditions. Cases in the lower-right and upper-left quadrants therefore correspond to different screening classifications by the two approaches.

The scaled barrier estimates will be used to screen a variety of substrates in the next section, where the most important factor is the accuracy of the barrier height of the RDS. Fig. 5 shows the corresponding plot for the RDS barriers. In most cases the RDS barrier is TS1 so the correlation is very similar to that observed to TS1, and the relatively poor performance observed for TS3 is unlikely to impact the conclusions. For example, if we impose 24 kcal mol^−1^ as the RDS-barrier cutoff for a viable substrate (discussed further below), then the TSG approach fails for only three cases. For tri(propan-2-yl)-pyrrol-1-ylsilane and trimethyl(pyrrol-1-yl)silane the TSG approach underestimates the TS1 barrier of position 4 and 3 by 7.0 and 4.7 kcal mol^−1^, respectively; while for 2-methoxyfuran the TS1 estimated barrier for position 4 is slightly overestimated at 25.6 kcal mol^−1^, while the actual barrier is 23.5 kcal mol^−1^.

### 3.2 Substrate screening

Having benchmarked the constrained-xTB/SP model (Δ*G*^‡,cal^_DFT_) against DFT reference barriers, we next apply it to substrate screening. We consider a subset of 69 aromatic substrates (totaling 267 C–H positions) from a dataset previously explored for iridium-catalysed C–H borylation.^47^ The set spans simple phenyl derivatives (phenols, anisoles, *N*-aryl amides), five-membered heterocycles (pyrazoles, indoles, benzoxazoles, benzimidazoles), and fused bicyclic systems (naphthalenes, quinolines, and related azaarenes).

Across the 267 evaluated (substrate, rpos) cases, the RDS is overwhelmingly associated with TS1: TS1 is predicted to be the RDS barrier in 257 cases (97%), with only a small fraction assigned to TS4 (1.5%) or TS2 (1.5%) (Fig. S4). Importantly, this dominance is not driven by widespread near-degeneracy. The separation between the highest and second-highest predicted barriers has a median of 5.08 kcal mol^−1^ (10th–90th percentile: 1.32–7.43 kcal mol^−1^), and only 9.0% of sites exhibit a margin below 1 kcal mol^−1^. Consistent with the benchmarking results, TS1 remains highly informative even when it is not strictly rate-determining: TS1 lies within 1 kcal mol^−1^ of the RDS barrier in 98.5% of cases. Finally, after calibration to the DFT reference scale, 30.7% of screened sites fall below the 30 kcal mol^−1^ viability threshold, motivating the focused subset of low-barrier candidates discussed next.

As we discuss below a practical RDS barrier cutoff for a viable substrate is about 24 kcal mol^−1^ and based on this cutoff we predict that 22 of the tested substrates are viable (Table S6). For 12 of these, the most active atom is in five-membered rings: six indoles, four pyrazoles, and two pyrropyridines. However, all 12 molecules contain an N–H site which is a concern because pyrrole has been experimentally shown not to be a viable substrate.^2^ We investigate this issue further below.

**Fig. 6:**
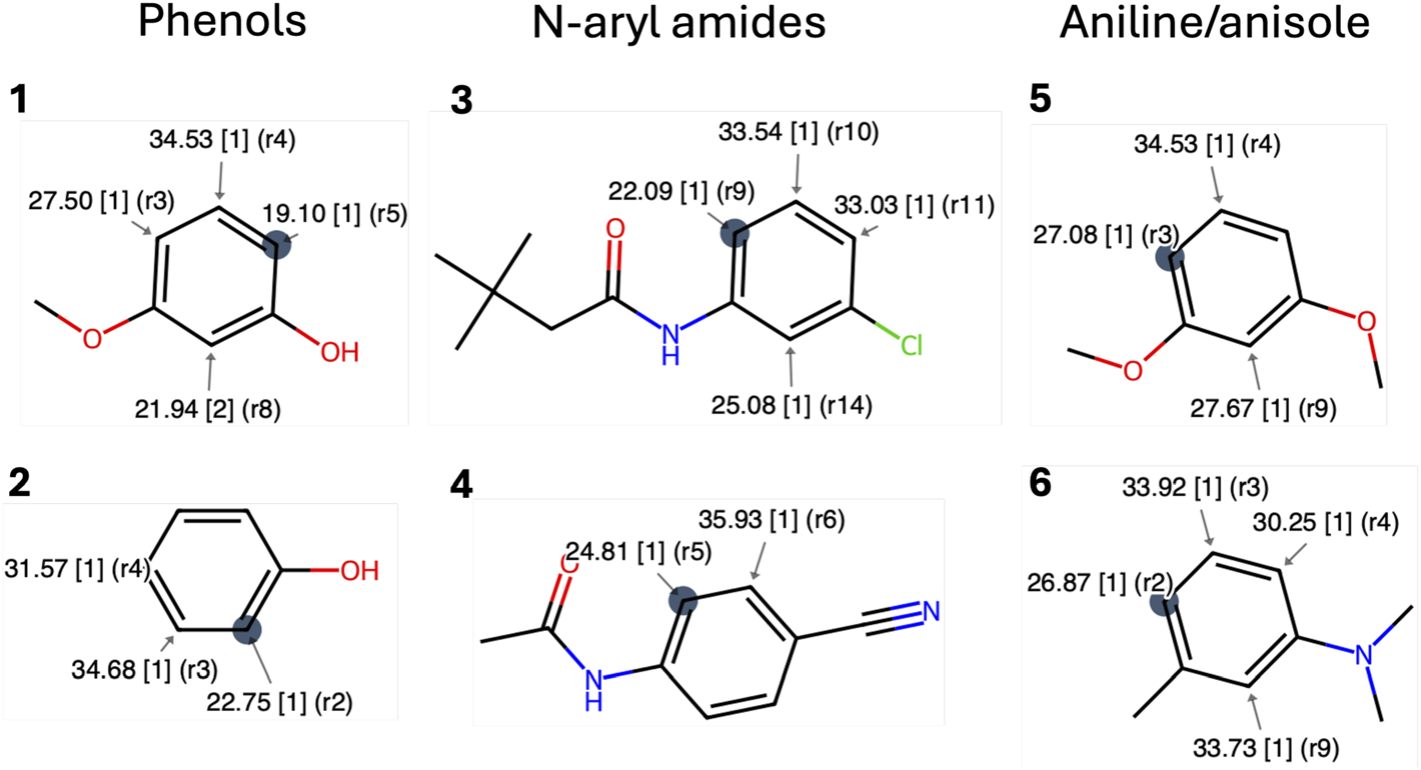
Selection of low-barrier candidates from the screening campaign. Each aromatic carbon is annotated with (i) the estimated RDS barrier (Δ*G*^‡,cal^_DFT_) among TS1–TS4 at that position, (ii) an index in square brackets indicating which transition state attains this maximum, *i.e.* which transition state defines the predicted RDS and (iii) in brackets the rpos number, that is used as an unique identifier. In 1, TS1 is predicted to be the RDS at all positions except one (rpos 8), where TS2 instead gives the largest barrier. Overall, TS1 is most frequently predicted as the RDS across the screening dataset.

Interestingly, for the remaining ten substrates the most active atom is in six-membered rings (nine phenols/naphthols and one *N*-aryl amide), which has not been observed experimentally. We therefore decided to investigate some of these cases further and selected two phenols (1 and 2 in Fig. 6) and the *N*-aryl amide 3, as well as an additional *N*-aryl amide (4) with an estimated RDS barrier just above the cutoff (24.8 kcal mol^−1^). All four of these substrates include protic heteroatoms that may introduce competitive side reactions, so we also selected two additional aprotic substrates (5 and 6) that have the lowest RDS barriers in that subclass, for further study (Fig. 6).

Both 1 and 2 exhibit relatively low predicted RDS barriers in the constrained-xTB/SP screen. In both cases, the most favourable reactive positions are adjacent to the hydroxyl group, with values of 19.10 and 22.75 kcal mol^−1^, respectively, and with TS1 defining the RDS at those sites. For the *N*-aryl amides, 3 and 4, the workflow predicts slightly higher but still favorable RDS barriers of 22.09 and 24.81 kcal mol^−1^, again with TS1 defining the RDS across the annotated positions. Finally, the anisole derivative 5 and the tertiary aniline 6 represent the lowest-barrier candidates in the absence of an acidic proton. Their predicted RDS barriers (27.08 and 26.87 kcal mol^−1^, respectively) suggest that kinetically accessible pathways may still be available.

#### 3.2.1 Validation of the aprotic compounds

**Fig. 7:**
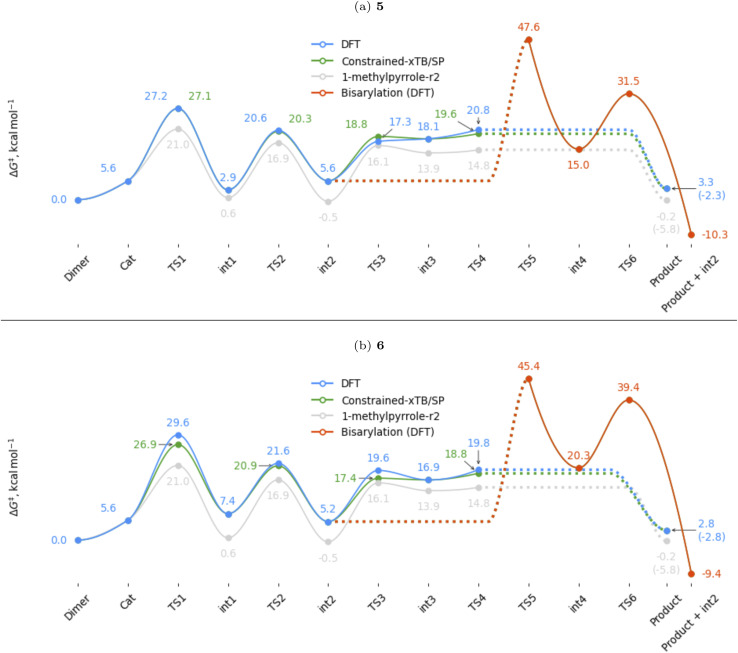
DFT validation of two representative screening hits, 5 and 6. For each substrate, the blue profile shows the DFT reference free-energy profile (Δ*G*^‡^_DFT_), with all states reported relative to the dimer (ωB97X-D/6-31G**). Relative product energy to the catalyst is given in parentheses. The green profile shows the corresponding constrained-xTB/SP barrier estimates for the transition states (Δ*G*^‡,cal^_DFT_), likewise referenced to the dimer. The orange line represents the bis-arylation reaction as shown on Fig. 1. Because the screening workflow yields calibrated barrier estimates for transition states only, the intermediate and product energies shown in the green profile are taken from the DFT reference profile and are included solely to provide the full mechanistic context. Thus, the comparison is intended to assess how well the constrained-xTB/SP model reproduces the relative heights and ordering of the key transition-state barriers along the catalytic cycle. For reference we have also shown the DFT free-energy RDS surface for a known substrate (*N*-methyl pyrrole, Fig. 1). DFT validation of two representative screening hits: (a) substrate 5 and (b) substrate 6.

We carried out DFT calculations for two representative low-barrier candidates selected from the screening campaign, namely 5 and 6. The resulting free-energy profiles are shown in Fig. 7.

Fig. 7 shows that, for both 5 and 6, the constrained-xTB/SP model correctly identifies TS1 as the transition state associated with the RDS when compared to DFT. For 5, the TS1 barrier is reproduced almost exactly, differing by only 0.1 kcal mol^−1^ between the two approaches. For 6, TS1 is underestimated by 2.7 kcal mol^−1^, but it remains clearly higher than the remaining barriers and is therefore still assigned as the RDS. The subleading barriers are also described well: for 6, the ordering is preserved exactly, whereas for 5 the only discrepancy is a reversal of TS2 and TS4. Since both TS2 and TS4 remain substantially lower in energy than TS1, this difference does not affect the mechanistic interpretation relevant to screening.

Both compounds have product energies that remain above the dimer reference (+3.3 and +2.8 kcal mol^−1^). This does not, however, imply that the catalytic transformation itself is thermodynamically unfavorable. The dimer is a dormant resting state rather than the active catalyst, and the +5.6 kcal mol^−1^ offset corresponds to the energetic cost of generating the catalytically competent monomer. Referencing energies to the dimer is therefore appropriate when discussing apparent activation barriers from the resting state, but less appropriate for judging the thermodynamics of product formation within the catalytic cycle. When referenced instead to the active catalyst, the products lie at −2.3 and −2.8 kcal mol^−1^ (shown in parentheses in Fig. 7), showing that product formation is favorable after catalyst activation.

We also show DFT calculations for the bis-arylation side reaction (orange line, Fig. 7) described by Lavergne *et al.*^3^ and Rochette *et al.*^5^ and illustrated in Fig. 1. In this pathway, a second substrate reacts with the catalyst-derived intermediate to form a bis-arylated complex (Fig. 1, int4). Although thermodynamically favored, this side reaction is unlikely to compete for either 5 or 6, as indicated by the high TS5 barriers (5: 47.6 kcal mol^−1^; 6: 45.4 kcal mol^−1^).

Having identified 5 and 6 as promising candidates, we next sought to define a practical barrier threshold for productive FLP borylation under the reported reaction conditions. To this end, DFT reaction profiles were computed for three substrates reported to be unreactive by Légaré *et al.*, namely Boc-protected pyrrole, 1-phenylpyrrole, and 1,3,5-trimethoxybenzene.^2^ As shown in Fig. S7, these substrates uniformly display RDSs near 25 kcal mol^−1^, with TS1 energies of 25.5, 25.0, and 25.4 kcal mol^−1^, respectively. These data provide a useful empirical upper bound for reactivity. In the present benchmark, substrates with barriers approaching 25 kcal mol^−1^ appear to lie beyond the range compatible with efficient turnover, whereas lower-barrier substrates remain viable.

This interpretation is supported by the full benchmark set. Among all experimentally successful substrate/rpos combinations, the highest computed rate-determining barrier is 23.1 kcal mol^−1^ for TMS-pyrrole (compound 3d in the study of Légaré *et al.*; see Table S7).^2^ Taken together, these results place the transition between productive and unproductive substrates within a narrow window. On this basis, a practical cutoff of *ca.* 23–24 kcal mol^−1^ appears reasonable: substrates below this range are likely to be competent under the reported conditions, whereas substrates with barriers near 25 kcal mol^−1^ are unlikely to undergo borylation. Accordingly, the computed barriers for 5 and 6 place these substrates on the non-reactive side of the empirical threshold, however, this also shows promise for future catalyst optimization as the gap to reactivity might be within range.

#### 3.2.2 Validation of compounds with protic heteroatoms

In most of the selected cases, the RDS assigned by the constrained-xTB/SP model is associated with TS1. One notable exception is 1, where one annotated C–H site (Δ*G*^pred^_TS2_ = 21.94 kcal mol^−1^) is predicted to be TS2-limited. To assess whether this reflects a genuine mechanistic shift or an artefact of the screening model, we carried out DFT calculations for this case. The DFT reference instead identifies TS1 as the rate-determining transition state, with TS1 at 26.30 kcal mol^−1^ and TS2 lower at 24.22 kcal mol^−1^, whereas the constrained-xTB/SP model underestimates TS1 by roughly 7 kcal mol^−1^ and slightly elevates TS2 (19.18 and 21.94 kcal mol^−1^, respectively), leading to an inverted RDS assignment. Notably, this inversion is specific to rpos 8: for the other evaluated sites on 1 (rpos 3, 4, and 5; see Fig. 6 for the rpos mapping), both DFT and the constrained-xTB/SP model identify TS1 as the transition state defining the RDS (DFT: 27.34, 33.46, 24.02 kcal mol^−1^*vs.* constrained-xTB/SP: 27.50, 34.53, 19.10 kcal mol^−1^ for TS1 at rpos 3, 4, and 5, respectively), and the ordering of TS barriers is preserved across these sites (Fig. S8). Importantly, although the constrained-xTB/SP model assigns TS2 as the RDS-defining transition state at rpos 8, this does not alter the qualitative screening conclusion for 1 as a low-barrier candidate.

For both *N*-aryl amides, the screening workflow correctly identifies TS1 as the rate-determining transition state. However, the DFT barriers for the productive C–H borylation pathway are substantially higher than the corresponding constrained-xTB/SP estimates from the screening workflow. For 3, the screening model predicts an RDS barrier of 22.09 kcal mol^−1^, whereas the corresponding DFT value is 29.7 kcal mol^−1^. For 4, the constrained-xTB/SP prediction is 24.81 kcal mol^−1^, while the DFT barrier is 31.1 kcal mol^−1^. Thus, due to an underestimation of TS1 (similar to what we observed for 1) the screening workflow underestimates the C–H barrier by 7.6 and 6.3 kcal mol^−1^ for 3 and 4, respectively. These cases illustrate the intended role and current limitation of the screening model. For substrate classes that are structurally distant from the benchmark set, the constrained-template barriers should be interpreted as candidate-generating estimates rather than definitive reactivity predictions. We therefore regard out-of-domain low-barrier cases as requiring full DFT TS optimization before being classified as viable. Conversely, imposing a hard applicability-domain cutoff at the screening stage could increase the number of false negatives by discarding unusual substrates before validation. A future implementation could attach confidence labels based on structural similarity to the benchmark set and on the margin between the RDS and second-highest predicted barriers.

#### 3.2.3 Computational cost

The main cost of estimating a barrier for a reaction at a particular site is 10 constrained xTB optimisations followed by a DFT single point, which can be done in a matter of minutes on a single CPU for the systems considered in this study. This cost then has to be multiplied by the number of barriers in the main catalytic cycle (four in our case) and the number of possible (unique) reaction sites, which ranges from 2 to 11 in this study. So, we are able to screen all 69 substrates in a couple of hours using *ca.* 200 CPU cores. The vast majority of the CPU time expended in this study is the DFT optimisations of TSs used to calibrate the method and validate a subset of the predictions. In future studies this CPU requirement can likely be reduced by replacing some or all DFT calculations by corresponding machine learning potential-calculations.

### 3.3 Side reactions involving protic heteroatoms

#### 3.3.1 Literature precedent for competing borylation pathways

Four of the six substrates chosen for further study (Fig. 6) contain protic heteroatoms, which may be involved in competing side reactions. Preshlock *et al.* note that sufficiently acidic N–H and O–H bonds react with boron hydrides to evolve H_2_ and form B–N or B–O bonds, whereas the N–H bonds of pyrrole and indole do not spontaneously react with HBpin to generate N-Bpin species.^48^ In the same study, aniline-derived substrates were shown to form mono-*N*-borylated intermediates rapidly under Ir-catalyzed borylation conditions, with the N–B bond persisting during the subsequent C–H borylation step.^48^ Taken together, these observations suggest that direct substrate borylation or pre-borylation is chemically plausible for aniline- and phenol-like substrates, but not necessarily for pyrrole-like systems.^48^

Second, related FLP literature suggests that protic substrates can generate stable off-cycle adducts that inhibit productive turnover. Rochette *et al.* examined the reactivity of the amine-borane FLP [NMe_2_-C_6_H_4_-BH_2_]_2_ toward alcohols, amines, and thiols, and found that alcohol- and amine-derived activation products are inert toward the subsequent metathesis step with HBpin 5. In other words, the initially formed activation products become trapped as stable intermediates and do not re-enter the productive catalytic cycle. The authors rationalized this behavior by noting that alkoxy and amido substituents can reduce the Lewis acidity of boron through lone-pair donation, thereby slowing or preventing metathesis with HBpin. Their DFT analysis supported this picture: for the *tert*-butylamine-derived system, the amido intermediate was found to be highly stable, lying 12.8 kcal mol^−1^ below the starting materials, and the second E–H activation step was associated with a barrier of 32.3 kcal mol^−1^ at the ωB97X-D/6-31+G** level. On this basis, Rochette *et al.* concluded that the presence of alcohols and protic amines can rationalize the lack of catalytic activity in FLP-mediated heteroarene borylation under such conditions.^5^

Taken together, these reports suggest two plausible side-reaction scenarios for the substrates examined here: direct substrate borylation, *i.e.* pre-borylation on O–H/N–H-containing arenes, and off-cycle trapping of the FLP catalyst or catalytic intermediates by protic functionality. The first step is to predict whether N/O-borylation is likely to occur spontaneously. If so, then C–H borylation must be investigated using the N/O-preborylated form as substrate. If not, then off-cycle trapping must be investigated. Spontaneous borylation of phenols has already been established experimentally, but is unknown for *N*-aryl amides and will be addressed next.

#### 3.3.2 Computational probe of spontaneous substrate borylation

The propensity for spontaneous substrate borylation is expected to depend on both the acidity of the proton and the nucleophilicity of the resulting conjugate base. Thus, acidity alone is not a reliable predictor: aniline, which undergoes spontaneous borylation, has a significantly higher p*K*_a_ (30.6 in DMSO) than pyrrole and indole (23.0 and 21.0 in DMSO, respectively), which do not. This indicates that borylation propensity cannot be rationalized solely in terms of proton acidity.

**Fig. 8:**
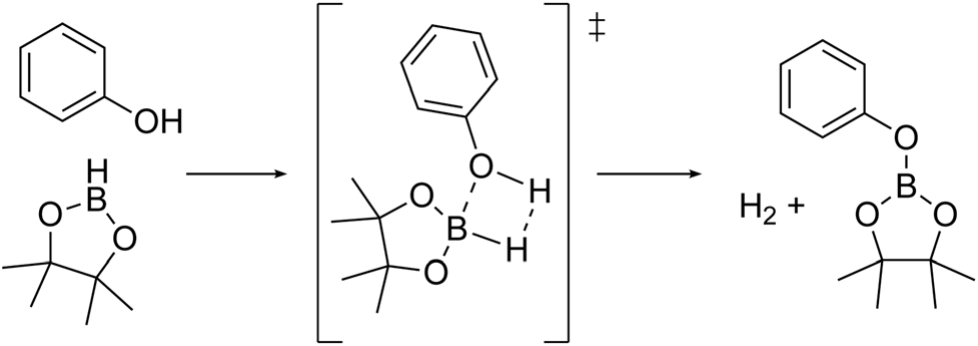
Concerted substrate-borylation pathway used to evaluate spontaneous borylation propensity. A protic substrate reacts directly with HBpin to form the corresponding heteroatom-Bpin adduct with concomitant release of H_2_. The computed barriers for this pathway are used as a qualitative measure of borylation propensity, distinguishing phenol- and aniline-like substrates from pyrrole- and indole-like substrates.

**Fig. 9:**
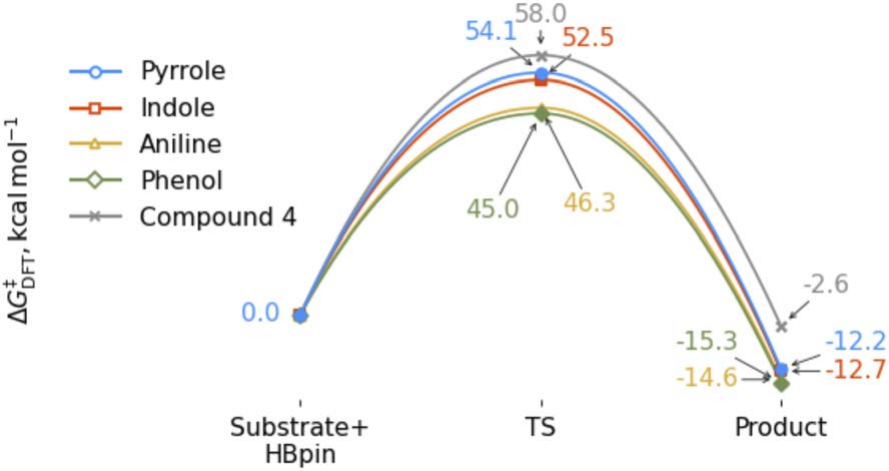
Computed free-energy profiles for direct substrate borylation by HBpin for phenol, aniline, pyrrole, and indole at the ωB97X-D/6-31+G** level of theory. Energies are reported relative to the separated substrate + HBpin reactants. The figure shows that all four reactions are exergonic overall, but that phenol and aniline have lower transition-state barriers than pyrrole and indole.

To probe this relationship more directly, we evaluate whether the experimentally observed reactivity correlates with the computed barrier for a concerted borylation pathway (Fig. 8) for phenol, aniline, pyrrole, indole, and compound 4 (Fig. 9). Although the absolute barriers for aniline and phenol are too high to be consistent with experiment, the relative values reveal a clear and chemically meaningful trend: phenol and aniline exhibit lower barriers (45.9 and 46.5 kcal mol^−1^, respectively) than pyrrole and indole (54.1 and 52.2 kcal mol^−1^, respectively), corresponding to a separation of approximately 6–8 kcal mol^−1^. This distinction reproduces the experimental behavior, where phenol- and aniline-like substrates undergo spontaneous borylation, while pyrrole- and indole-like substrates do not.

While the overall reaction energies are also consistent with experiment (−15.3, −14.6, −12.2, and −12.7 kcal mol^−1^ for phenol, aniline, pyrrole, and indole, respectively), these thermodynamic differences are comparatively small and do not distinguish the substrate classes as clearly. In contrast, the barrier heights provide a more sensitive and discriminating metric for borylation propensity.

Taken together, these results indicate that the concerted barrier captures the key features governing spontaneous borylation and can therefore be used predictively. On this basis, the computed barrier for compound 4 (58.0 kcal mol^−1^) places it in the high-barrier regime, and we predict that it will not undergo spontaneous borylation.

#### 3.3.3 Phenol-Bpin as an alternative substrate

**Fig. 10:**
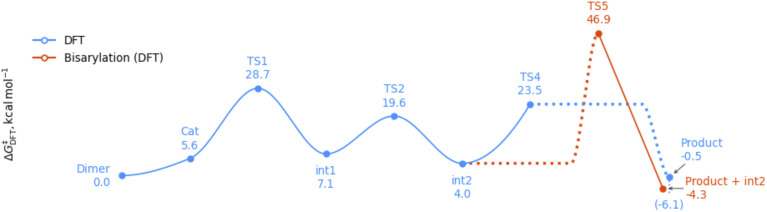
DFT free-energy profile for the catalytic cycle starting from phenol-Bpin as the substrate at the ωB97X-D/6-31G** level of theory. Energies are reported relative to the dimer reference. Relative product energy to the catalyst is given in parentheses. The blue profile corresponds to the productive pathway, while the orange profile shows the competing bis-arylation side pathway. In this model, TS1 defines the RDS of the productive cycle (28.7 kcal mol^−1^), whereas the bis-arylation pathway is disfavored by a substantially higher TS5 barrier of 46.9 kcal mol^−1^.

Fig. 10 shows the reaction profile for C–H borylation of the pre-borylated phenol. The first step is rate determining with a barrier 28.7 kcal mol^−1^, significantly higher than the 25 kcal mol^−1^ cutoff discussed above. Thus our prediction is that phenols are not viable substrates for the FLP catalyst considered in this study.

#### 3.3.4 Why does pyrrole fail?

**Fig. 11:**
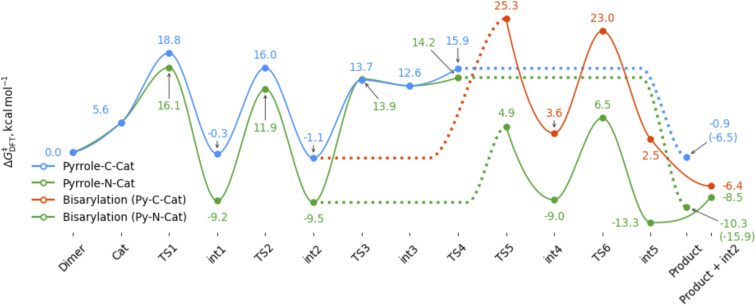
DFT free-energy profiles for pyrrole comparing conventional C–H borylation (blue) with activation at the N–H position (green) at the ωB97X-D/6-31G** level of theory. Energies are reported relative to the dimer reference. Relative product energies to the catalyst is given in parentheses. The C–H borylation pathway is mildly exergonic and does not contain a prohibitively high barrier, whereas the N–H-derived pathway gives access to substantially more stable intermediates, including species within the bis-arylation manifold.

Before testing whether off-cycle trapping occurs for *N*-aryl amides, we first address the same question for pyrrole, since that has been experimentally determined to give no yield (Fig. 11). The blue profile corresponds to conventional C–H borylation at the position adjacent to nitrogen, whereas the green profile corresponds to N–H activation. The productive C–H pathway is not prohibitively high in energy: it is slightly exergonic overall, with the product at −0.9 kcal mol^−1^ relative to the dimer, and its RDS (TS1) is only 18.8 kcal mol^−1^. On its own, this pathway would therefore appear compatible with catalytic turnover.

In contrast, N–H activation leads into a lower-energy off-cycle manifold. The first two barriers along this pathway, TS1 and TS2, are 16.1 and 11.9 kcal mol^−1^, respectively, and are lower than those calculated for C–H borylation, while the resulting intermediates are substantially more stable than the productive C–H borylation product at −0.9 kcal mol^−1^. Specifically, int1 and int2 lie at −9.2 and −9.5 kcal mol^−1^, while the bis-arylation intermediate int5 is lower still at −13.3 kcal mol^−1^. Although the N–H-derived product is itself exergonic at −8.5 kcal mol^−1^, it is not the thermodynamic sink of the profile. This is consistent with the experimental observation that a pyrrole-Bpin product is not formed, and instead suggests that pyrrole is diverted into a low-barrier trapping manifold rather than proceeding through productive C–H borylation.

One related side reaction that we have not considered so far is the dimerisation of int2, which could make that part of the cycle the thermodynamic sink, if it is as exergonic as for the catalyst itself. However, our calculations show that the dimerization of int2 (from the C-borylation pathway) is endergonic by 5.4 kcal mol^−1^ and thus not an issue. Since pyrrole is sterically less demanding than the larger substrates considered elsewhere in this study, these results suggest that dimerization is unlikely to become more favorable for those cases as well.

In conclusion, the calculations point to trapping, rather than an inaccessible productive barrier, or a resting dimeric state, as the more likely reason that pyrrole fails in FLP-mediated borylation. Within the present model, productive C–H borylation is energetically feasible, but N–H activation opens a lower-energy off-cycle manifold that is more consistent with the experimentally observed lack of reactivity.

#### 3.3.5 *N*-aryl amides

Two *N*-aryl amides, 3 and 4 (see Fig. 6), were selected in the screening campaign because they were the lowest-barrier representatives of that subclass, with estimated RDS barriers of 22.09 and 24.81 kcal mol^−1^, respectively. Unlike the aprotic screening hits, however, both compounds contain an N–H moiety, which is predicted not to be spontaneously borylated. In light of the trapping behavior identified above for pyrrole, we therefore examined whether these substrates might likewise be diverted into an N–H-derived off-cycle manifold.

**Fig. 12:**
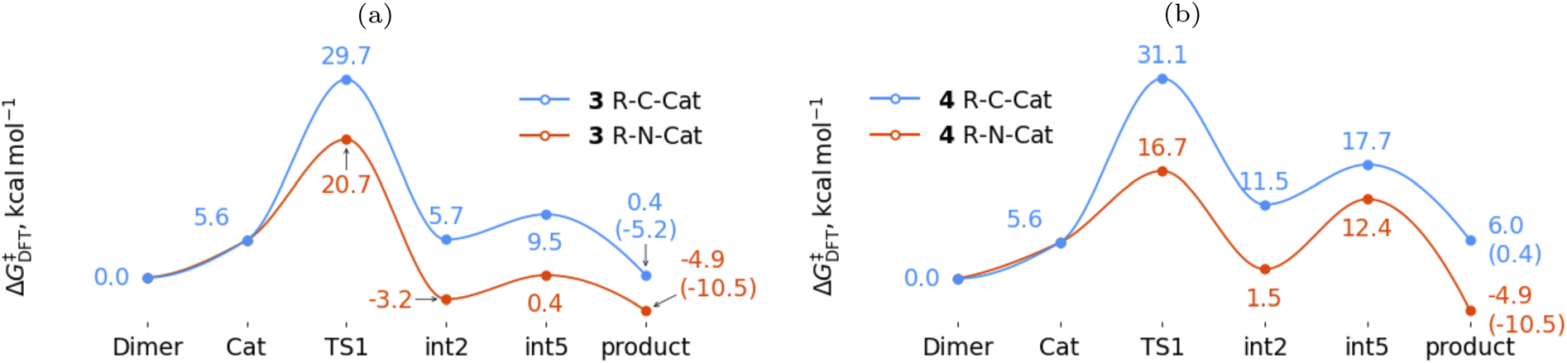
DFT free-energy profiles for the two *N*-aryl amide substrates: (a) compound 3 and (b) compound 4, computed at the ωB97X-D/6-31G** level of theory. Energies are reported relative to the catalyst dimer. Relative product energies to the catalyst are given in parentheses. In each panel, the blue profile corresponds to the productive C–H borylation pathway, whereas the orange profile corresponds to activation at the N–H position.

The resulting DFT profiles are shown in Fig. 12. In both cases, the N–H-derived pathway is thermodynamically more favorable than the corresponding C–H borylation pathway: the N–H-derived product lies 5.3 kcal mol^−1^ lower than the C–H product for 3 and 10.9 kcal mol^−1^ lower for 4. Nevertheless, unlike pyrrole, these *N*-aryl amides do not show the same signature of trapping. For both substrates, all intermediates along the N–H-derived pathway remain above the final N–H-derived product. Thus, although N–H activation is thermodynamically preferred, the calculations do not indicate accumulation in especially deep off-cycle minima analogous to those found for pyrrole. Our prediction is therefore that the FLP catalyst will *N*-borylate the amide N–H group. However, subsequent C–H borylation is unlikely, given the high barriers for C–H borylation for these two substrates.

## 4 Summary and outlook

We have presented FRUST, a computational workflow for predicting substrate scope in catalytic reactions, and applied it to metal-free C–H borylation mediated by the boron-based frustrated Lewis pair catalyst developed by Fontaine and co-workers. The central challenge addressed in this work is that substrate scope prediction requires more than identifying a single low-energy pathway: to assess whether a substrate is viable, one must estimate the relative energetics of multiple steps across the catalytic cycle and, where relevant, consider competing processes that can suppress productive turnover.

FRUST approaches this problem through automated generation of intermediates and transition-state guess structures for each unique aromatic C–H position, followed by conformational sampling and barrier estimation using constrained xTB geometries with DFT single-point refinement. Benchmarking against literature substrates showed that this approximate strategy captures the dominant mechanistic trends of the FLP catalytic cycle and reproduces the RDS in most cases. In particular, TS1 emerged as the dominant barrier-defining transition state both in the benchmark set and across the broader substrate screen, suggesting that it may serve as a useful first-pass indicator in future screening applications.

Application of the workflow to 69 aromatic substrates identified a limited number of low-barrier candidates spanning phenols, *N*-aryl amides, and selected aprotic arenes. However, targeted DFT validation showed that even the most promising aprotic screening hits, 5 and 6, lie close to or beyond the empirical reactivity window defined by experimentally successful and unsuccessful literature substrates. Thus, while the workflow is able to identify comparatively favorable candidates, the present study also suggests that the current FLP catalyst is unlikely to achieve broad substrate scope beyond the activated heteroarenes already known experimentally.

Follow-up DFT analysis of representative side reactions further clarified why low predicted barriers in the productive C–H borylation cycle are not, by themselves, sufficient to guarantee productive reactivity. For phenol-containing substrates, initial borylation of the O–H group provides a plausible deactivation pathway: formation of the phenoxy-Bpin adduct is expected to inactivate neighbouring C–H sites toward subsequent productive borylation. For the *N*-aryl amides, a related but distinct limitation is predicted. The FLP catalyst is likely to borylate the amide N–H group preferentially; however, the resulting species is not expected to undergo subsequent C–H borylation, because the corresponding C–H activation barriers remain too high. These results highlight that computational substrate-scope prediction must account not only for the productive catalytic cycle, but also for heteroatom borylation and other mechanistic failure modes that can suppress productive turnover.

Several limitations of the present workflow point directly to opportunities for future development. First, the transition-state templates are derived from a relatively narrow set of reference systems and appear to be less transferable to substrate classes such as *N*-aryl amides. Expanding the template library, or replacing fixed templates with more adaptive transition-state generation strategies, could improve robustness across broader chemical space. Second, the present workflow is designed primarily to estimate barriers in the productive catalytic cycle. A more complete substrate-scope predictor should also include automated identification and ranking of plausible side reactions,^49,50^ especially for substrates bearing protic or strongly Lewis-basic functionalities. Third, the current screening protocol relies on a linear calibration between xTB-derived and DFT reference barriers. While effective in the present case, this strategy will likely benefit from larger and more chemically diverse benchmark sets.

More broadly, this study shows that computational substrate-scope prediction can be made sufficiently efficient to evaluate many substrates and reactive positions while still retaining mechanistic resolution. Although the present FLP catalyst appears to remain limited in scope, the workflow provides a practical foundation for future catalyst discovery and optimization efforts. In particular, it should be well suited to identifying structural modifications that lower the key barrier(s) for marginal substrates such as 5 and 6, and to guiding the design of next-generation metal-free borylation catalysts with improved activity and functional-group tolerance.

## Author contributions

Jacob M. Nielsen: methodology, software, validation, formal analysis, investigation, data curation, visualization and writing—original draft. Maria H. Rasmussen: methodology, validation, supervision and writing—review and editing. Jan H. Jensen: conceptualization, methodology, supervision, funding acquisition, project administration and writing—review and editing.

## Conflicts of interest

There are no conflicts to declare.

## Supplementary Material

SC-OLF-D6SC04421B-s001

SC-OLF-D6SC04421B-s002

## Data Availability

Paper repository: https://github.com/jensengroup/frust-paper. FRUST repository: https://github.com/MolinDiscovery/FRUST. Supplementary information (SI) is available. See DOI: https://doi.org/10.1039/d6sc04421b.

## References

[cit1] Rana D., Pflüger P. M., Hölter N. P., Tan G., Glorius F. (2024). Standardizing Substrate Selection: A Strategy toward Unbiased Evaluation of Reaction Generality. ACS Cent. Sci..

[cit2] Légaré M.-A., Courtemanche M.-A., Rochette É., Fontaine F.-G. (2015). Metal-free catalytic C-H bond activation and borylation of heteroarenes. Science.

[cit3] Légaré Lavergne J., Jayaraman A., Castro L. C. M., Rochette É., Fontaine F.-G. (2017). Metal-Free Borylation of Heteroarenes Using Ambiphilic Aminoboranes: On the Importance of Sterics in Frustrated Lewis Pair C–H Bond Activation. J. Am. Chem. Soc..

[cit4] Dalton T., Faber T., Glorius F. (2021). C–H Activation: Toward Sustainability and Applications. ACS Cent. Sci..

[cit5] Rochette É., Boutin H., Fontaine F.-G. (2017). Frustrated Lewis Pair Catalyzed S–H Bond Borylation. Organometallics.

[cit6] Grimme S. (2019). Exploration of Chemical Compound, Conformer, and Reaction Space with Meta-Dynamics Simulations Based on Tight-Binding Quantum Chemical Calculations. J. Chem. Theory Comput..

[cit7] Peters B., Heyden A., Bell A. T., Chakraborty A. (2004). A growing string method for determining transition states: Comparison to the nudged elastic band and string methods. J. Chem. Phys..

[cit8] Jónsson H. , Mills G., and Jacobsen K. W., Nudged elastic band method for finding minimum energy paths of transitions, in Classical and Quantum Dynamics in Condensed Phase Simulations, World Scientific, 1998, pp. 385–404

[cit9] Henkelman G., Uberuaga B. P., Jónsson H. (2000). A climbing image nudged elastic band method for finding saddle points and minimum energy paths. J. Chem. Phys..

[cit10] Ren W. E. W., Vanden-Eijnden E. (2002). String method for the study of rare events. Phys. Rev. B: Condens. Matter Mater. Phys..

[cit11] Bhoorasingh P. L., Slakman B. L., Seyedzadeh Khanshan F., Cain J. Y., West R. H. (2017). Automated Transition State Theory Calculations for High-Throughput Kinetics. J. Phys. Chem. A.

[cit12] Zimmerman P. M. (2013). Automated discovery of chemically reasonable elementary reaction steps. J. Comput. Chem..

[cit13] Van de Vijver R., Zádor J. (2020). KinBot: Automated stationary point search on potential energy surfaces. Comput. Phys. Commun..

[cit14] Zimmerman P. M. (2015). Single-ended transition state finding with the growing string method. J. Comput. Chem..

[cit15] Maeda S., Ohno K., Morokuma K. (2013). Systematic exploration of the mechanism of chemical reactions: The global reaction route mapping (GRRM) strategy using the ADDF and AFIR methods. Phys. Chem. Chem. Phys..

[cit16] Rasmussen M. H., Jensen J. H. (2020). Fast and automatic estimation of transition state structures using tight binding quantum chemical calculations. PeerJ Phys. Chem..

[cit17] Vaucher A. C., Reiher M. (2018). Minimum energy paths and transition states by curve optimization. J. Chem. Theory Comput..

[cit18] Ingman V. M., Schaefer A. J., Andreola L. R., Wheeler S. E. (2021). QChASM: Quantum chemistry automation and structure manipulation. WIREs Comput. Mol. Sci..

[cit19] Cerjan C. J., Miller W. H. (1981). On finding transition states. J. Chem. Phys..

[cit20] Banerjee A., Adams N., Simons J., Shepard R. (1985). Search for stationary points on surfaces. J. Phys. Chem..

[cit21] Baker J. (1986). An algorithm for the location of transition states. J. Comput. Chem..

[cit22] Barkema G. T., Mousseau N. (1996). Event-based relaxation of continuous disordered systems. Phys. Rev. Lett..

[cit23] Mousseau N., Barkema G. T. (1998). Traveling through potential energy landscapes of disordered materials: The activation-relaxation technique. Phys. Rev. E.

[cit24] Henkelman G., Jónsson H. (1999). A dimer method for finding saddle points on high dimensional potential surfaces using only first derivatives. J. Chem. Phys..

[cit25] Bernhard Schlegel H. (2003). Exploring potential energy surfaces for chemical reactions: An overview of some practical methods. J. Comput. Chem..

[cit26] Heyden A., Bell A. T., Keil F. J. (2005). Efficient methods for finding transition states in chemical reactions: Comparison of improved dimer method and partitioned rational function optimization method. J. Chem. Phys..

[cit27] Cancès E., Legoll F., Marinica M.-C., Minoukadeh K., Willaime F. (2009). Some improvements of the activation-relaxation technique method for finding transition pathways on potential energy surfaces. J. Chem. Phys..

[cit28] Bernhard Schlegel H. (2011). Geometry optimization. WIREs Comput. Mol. Sci..

[cit29] Behn A., Zimmerman P. M., Bell A. T., Head-Gordon M. (2011). Efficient exploration of reaction paths *via* a freezing string method. J. Chem. Phys..

[cit30] Stuyver T. (2024). TS-tools: Rapid and automated localization of transition states based on a textual reaction SMILES input. J. Comput. Chem..

[cit31] Yuan E. C.-Y., Kumar A., Guan X., Hermes E. D., Rosen A. S., Zádor J., Head-Gordon T., Blau S. M. (2024). Analytical *ab initio* hessian from a deep learning potential for transition state optimization. Nat. Commun..

[cit32] Simon S. L., Kaistha N., Agarwal V. (2026). An active learning algorithm for identifying transition states on a potential energy surface. J. Chem. Theory Comput..

[cit33] Koda S.-I., Saito S. (2026). dmf-g16: A Gaussian wrapper for reliable double-ended transition-state searches with native input formats. J. Comput. Chem..

[cit34] Sawatlon B., Wodrich M. D., Corminboeuf C. (2020). Probing Substrate Scope with Molecular Volcanoes. Org. Lett..

[cit35] Guan Y., Ingman V. M., Rooks B. J., Wheeler S. E. (2018). AARON: An automated reaction optimizer for new catalysts. J. Chem. Theory Comput..

[cit36] Jacobson L. D., Bochevarov A. D., Watson M. A., Hughes T. F., Rinaldo D., Ehrlich S., Steinbrecher T. B., Vaitheeswaran S., Philipp D. M., Halls M. D., Friesner R. A. (2017). Automated transition state search and its application to diverse types of organic reactions. J. Chem. Theory Comput..

[cit37] Zhao Q., Singla V., Hsu H.-H., Savoie B. M. (2025). Graphically defined model reactions are extensible, accurate, and systematically improvable. J. Chem. Theory Comput..

[cit38] Seumer J., Hansen J. K. S., Nielsen M. B., Jensen J. H. (2023). Computational evolution of new catalysts for the morita-baylis-hillman reaction. Angew Chem. Int. Ed. Engl..

[cit39] Young T. A., Silcock J. J., Sterling A. J., Duarte F. (2021). autodE: Automated calculation of reaction energy profiles- application to organic and organometallic reactions. Angew Chem. Int. Ed. Engl..

[cit40] Kozuch S., Martin J. M. L. (2011). The Rate-Determining Step is Dead. Long Live the Rate-Determining State. ChemPhysChem.

[cit41] Pracht P. , Caldeweyher E., Ehlert S., and Grimme S.. A Robust Non-Self-Consistent Tight-Binding Quantum Chemistry Method for large Molecules, ChemRxiv, 2019, preprint, 10.26434/chemrxiv.8326202.v1

[cit42] Bannwarth C., Ehlert S., Grimme S. (2019). GFN2-xTB—An Accurate and Broadly Parametrized Self-Consistent Tight-Binding Quantum Chemical Method with Multipole Electrostatics and Density-Dependent Dispersion Contributions. J. Chem. Theory Comput..

[cit43] RDKit. RDKit: Open-source cheminformatics. https://www.rdkit.org, 2024, Version 2024.09.5

[cit44] Chai J.-D., Head-Gordon M. (2008). Long-range corrected hybrid density functionals with damped atom–atom dispersion corrections. Phys. Chem. Chem. Phys..

[cit45] Neese F. (2025). Software Update: The ORCA Program System—Version 6.0. WIREs Comput. Mol. Sci..

[cit46] Bannwarth C., Caldeweyher E., Ehlert S., Hansen A., Pracht P., Seibert J., Spicher S., Grimme S. (2021). Extended tight-binding quantum chemistry methods. WIREs Comput. Mol. Sci..

[cit47] Borup R. M. , Jorner K., and Jensen J. H.. Chemically intuitive descriptors for predicting c–h borylation regioselectivity. ChemRxiv, 2026, preprint, 10.26434/chemrxiv.15004046/v1

[cit48] Preshlock S. M., Plattner D. L., Maligres P. E., Krska S. W., Maleczka Jr. R. E., Smith III M. R. (2013). A Traceless Directing Group for C-H Borylation. Angew. Chem., Int. Ed..

[cit49] Rasmussen M. H., Seumer J., Jensen J. H. (2023). Toward De Novo Catalyst Discovery: Fast Identification of New Catalyst Candidates for Alcohol-Mediated Morita–Baylis–Hillman Reactions. Angew. Chem., Int. Ed..

[cit50] SeumerJ. , RasmussenM. H., BreaO., MuuronenM., GöllerA. H., and JensenJ. H.. Practical computational exploration of chemical reaction space: Assessing the chemical stability of active pharmaceutical ingredients under acidic conditions, ChemRxiv, 2026, preprint, 10.26434/chemrxiv.15004121/v142733353

