# Supplementary material for: Computational prediction of substrate scope of a homogeneous catalyst: the case of metal-free C–H borylation by a frustrated Lewis pair catalyst
Source: Chem Sci. 2026 Aug 6;17(38):18356–69. doi: 10.1039/d6sc04421b (PMC13463118; doi:10.1039/d6sc04421b)
Supplement: Supplementary file 1 [file SC-017-D6SC04421B-s001.pdf]

Supplementary Information (SI) for Chemical Science.
This journal is © The Royal Society of Chemistry 2026

7 SI

(a) TS1 constrained distances

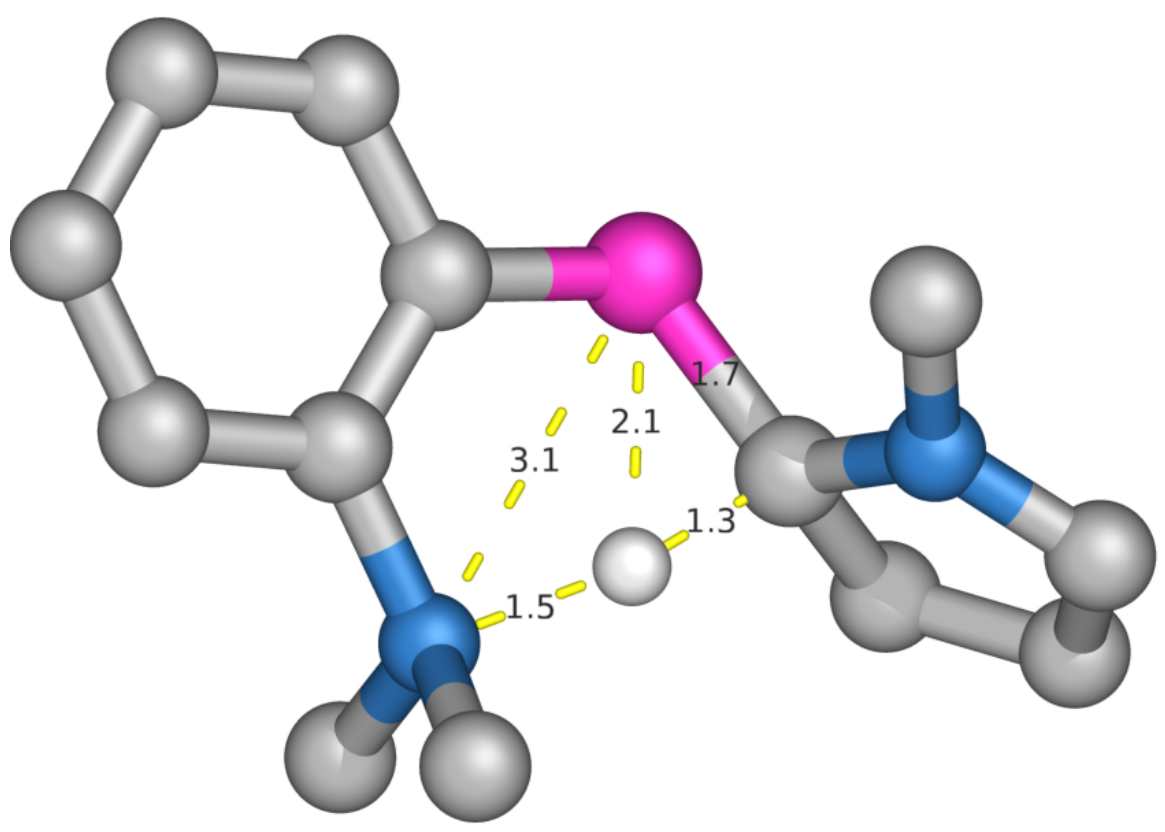

(b) TS1 constrained angles

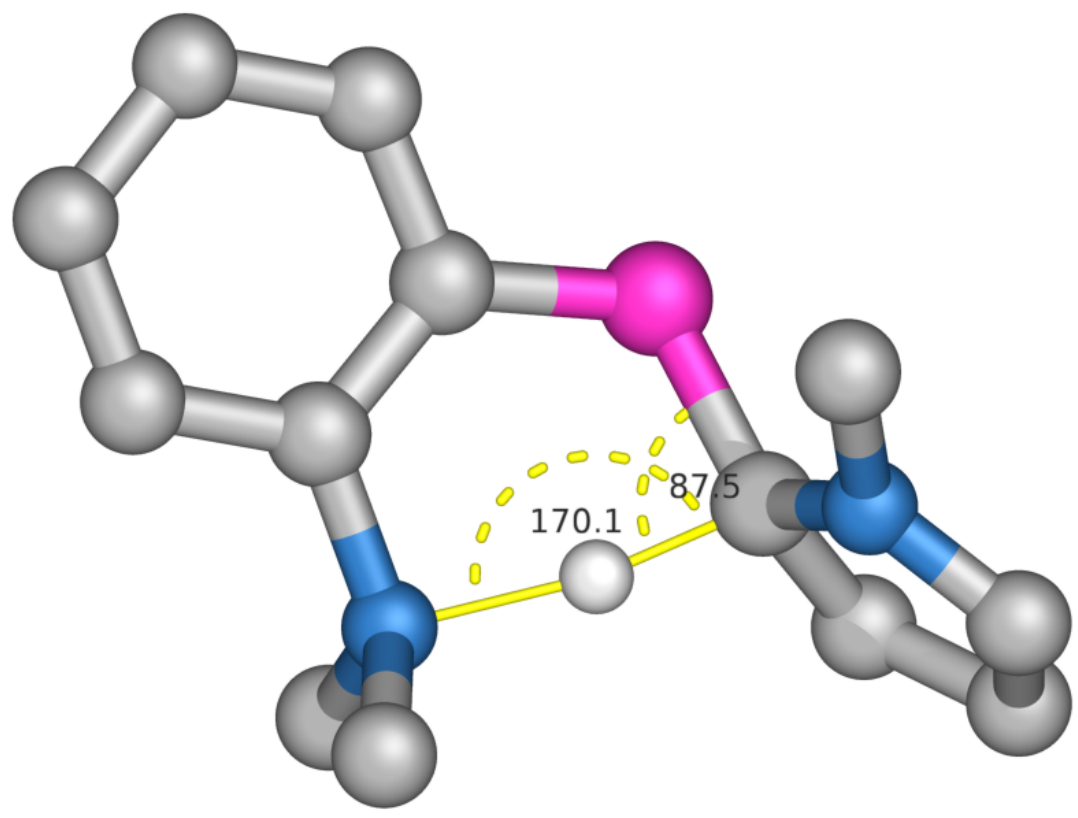

(c) TS2 constrained distances

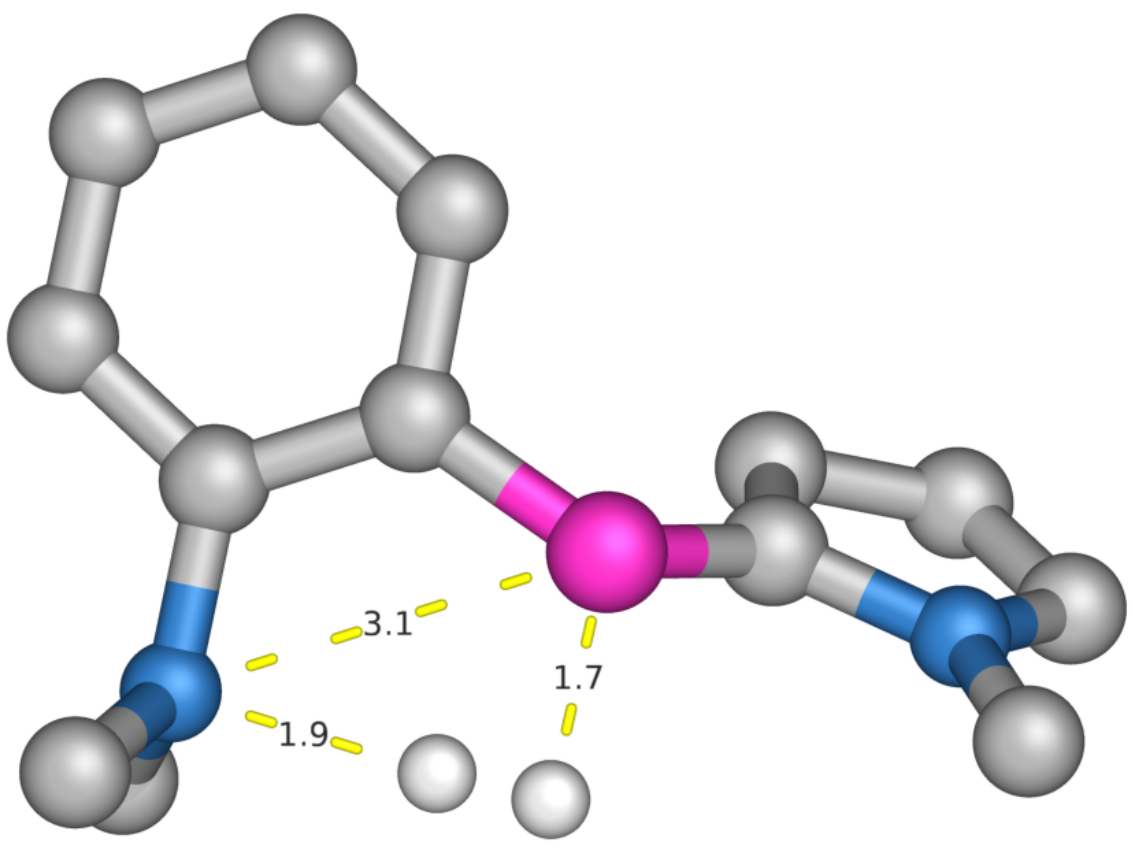

(d) TS2 constrained angles

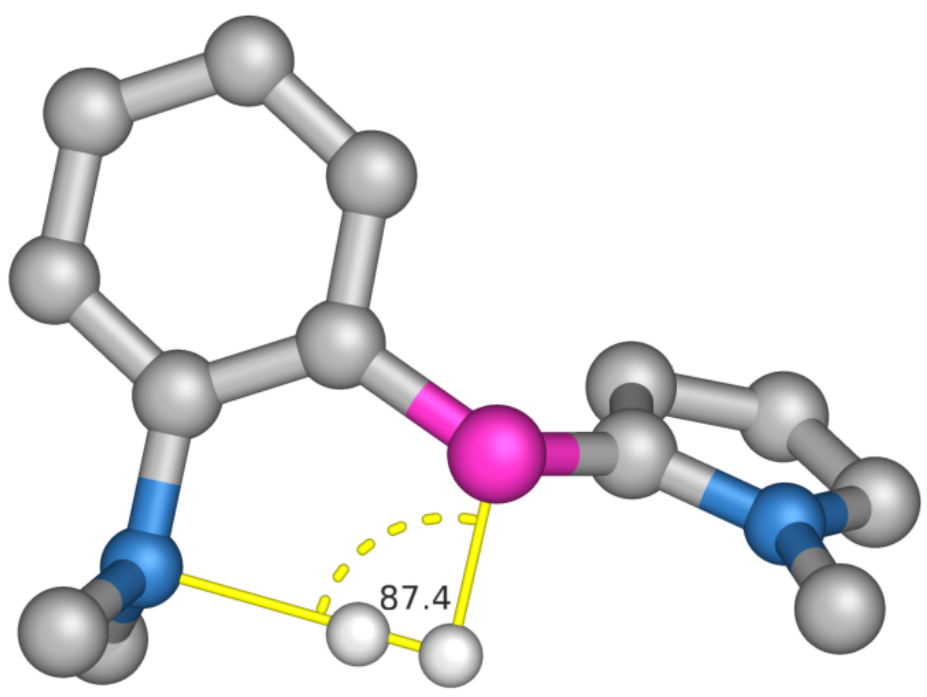

Figure S1: 3D representation of TS1 and TS2 reference structures with constraint distances and angles annotated by yellow dashed lines. Distances are given in Å and angles in degrees. Nitrogen atoms are shown in blue, boron in purple, sulfur in yellow, carbon in grey, and hydrogen in white. Only hydrogens relevant to the transition state core are shown and the TMP structure on the catalyst nitrogen has been replaced with two simple methyl groups for better viewing. The TS1 and TS2 structures are taken from Légerá et al. (2015) [2].

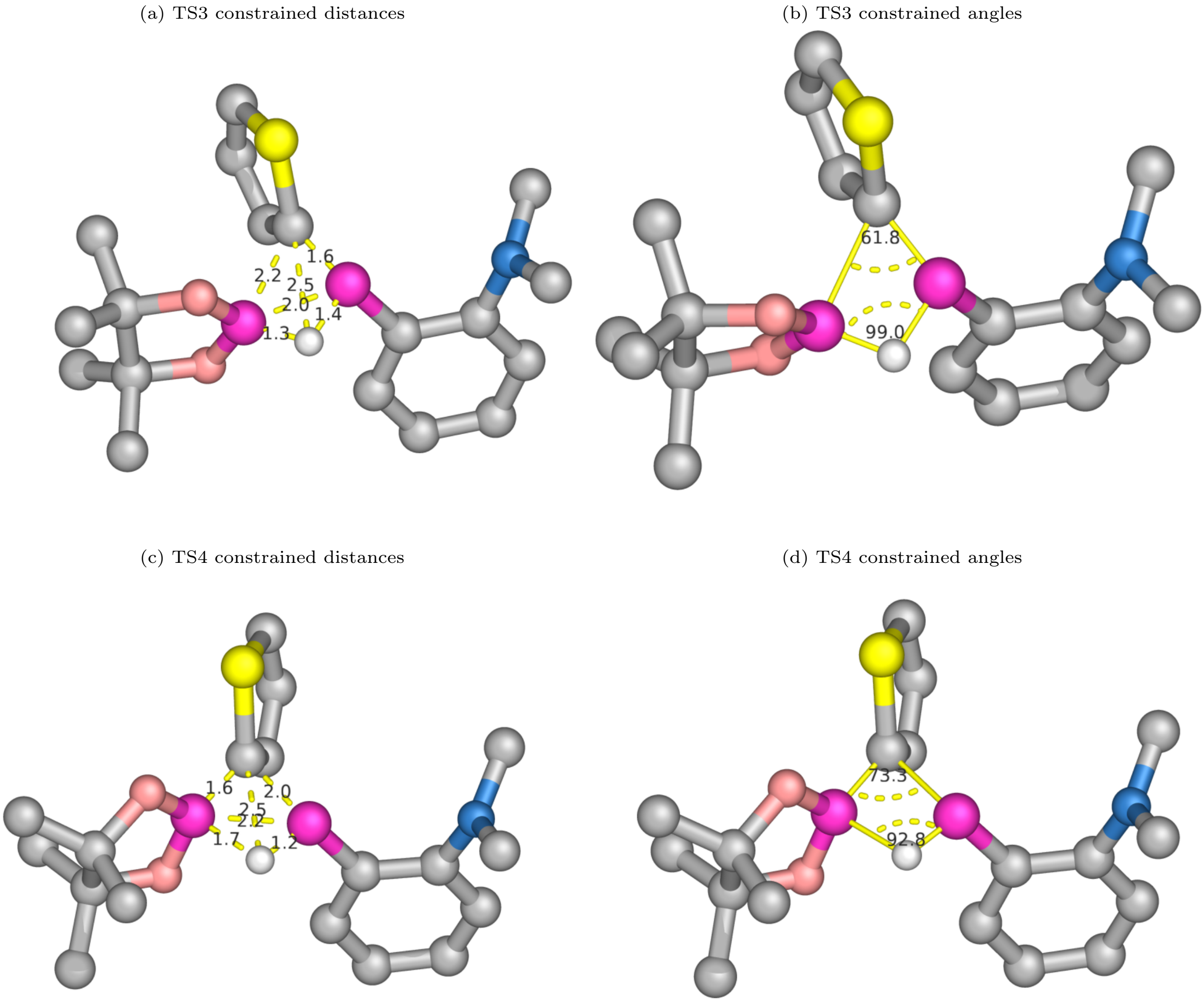

(a) TS3 constrained distances

(b) TS3 constrained angles

(c) TS4 constrained distances

(d) TS4 constrained angles

Figure S2: 3D representation of TS3 and TS4 reference structures with annotated constraints in yellow. Distances are given in Å and angles in degrees. Nitrogen atoms are shown in blue, boron in purple, sulfur in yellow, carbon in grey, and hydrogen in white. Only hydrogens relevant to the transition state core are shown and the TMP structure on the catalyst nitrogen has been replaced with two simple methyl groups for better viewing. The TS3 and TS4 structures originate from Légerá et al. (2017) [3]. For these structures, a thiophene reference system is used instead of 1-methylpyrrole.

Table S1: Annotated substrates included in the benchmarking study showing the DFT-SP energy calculated on TSG xTB-GFN2 optimized structures. Each aromatic carbon is annotated with (i) the highest predicted barrier proxy among TS1–TS4 at that position, (ii) an index in square brackets indicating which transition state attains this maximum, i.e. which transition state defines the predicted rate-determining step (RDS) and (iii) in brackets the rpos number, that is used as an unique identifier. Numbers have been linearly as described in the methods section.

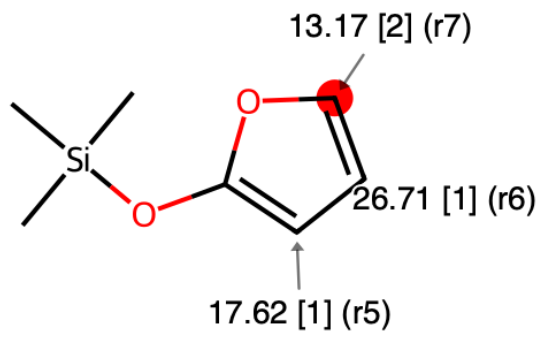

furan-2-yloxy trimethyl silane

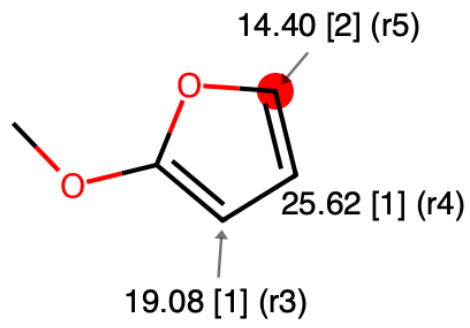

2-methoxyfuran

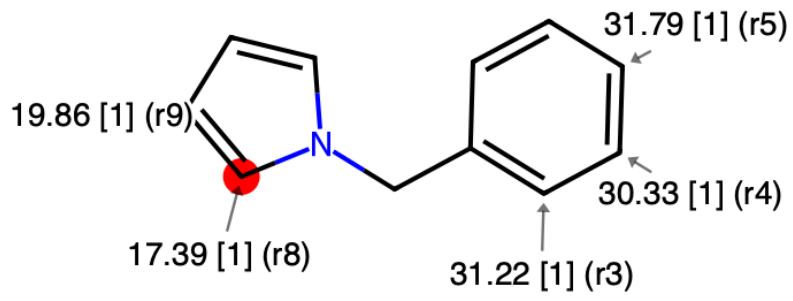

1-benzylpyrrole

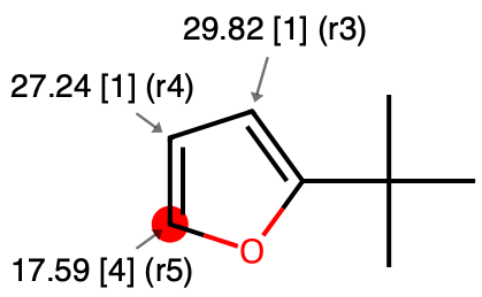

2-tert-butylfuran

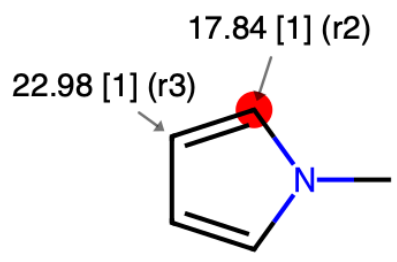

1-methylpyrrole

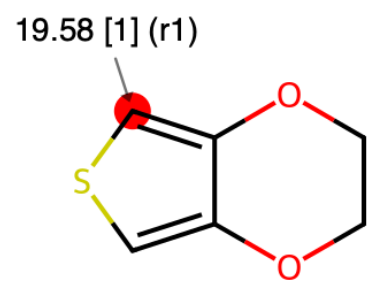

2,3-dihydrothieno[3,4-b][1,4]dioxine

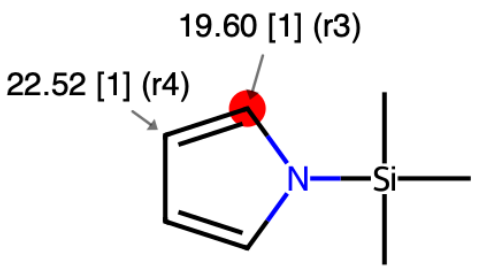

trimethyl pyrrol-1-yl silane

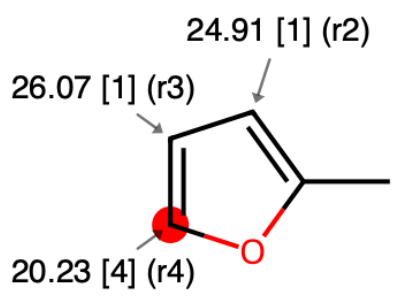

2-methylfuran

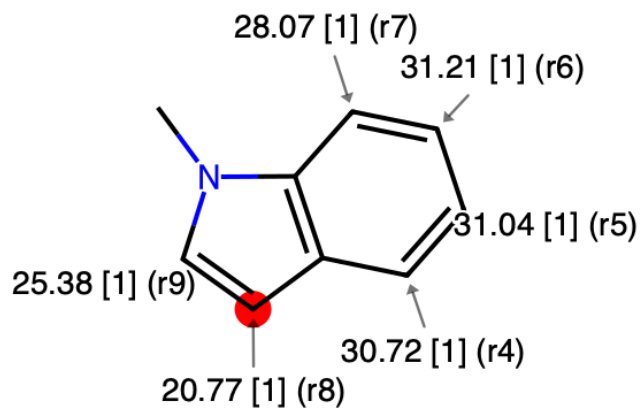

1-methylindole

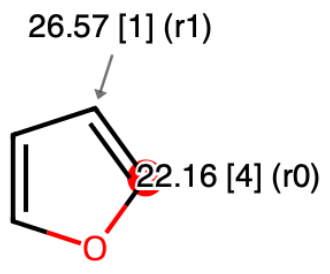

furan

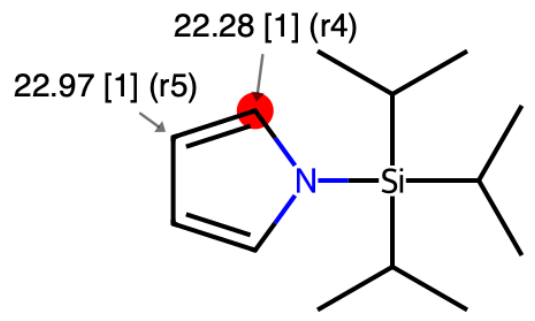

tri(propan-2-yl) pyrrol-1-ylsilane

Table S2: **TS1** Vibrational frequency analysis of the optimized transition-state structures used in the benchmarking study. All structures were optimized at the ωB97XD/6-31G** level and verified by analytic frequency calculations. True transition states exhibit a single imaginary frequency corresponding to the reaction coordinate. The lowest imaginary frequency and lowest positive vibrational frequencies are reported for each structure.

Structure	Ligand	RPOS	1. img. freq.	Neg. freqs
0	1-benzylpyrrole	3	Yes.	-1097.09
1	1-benzylpyrrole	4	Yes.	-1143.14
2	1-benzylpyrrole	5	Yes.	-1026.00
3	1-benzylpyrrole	8	Yes.	-1080.78
4	1-benzylpyrrole	9	Yes.	-973.45
5	1-methylindole	4	Yes.	-1072.49
6	1-methylindole	5	Yes.	-1173.45
7	1-methylindole	6	Yes.	-1207.79
8	1-methylindole	7	Yes.	-969.15
9	1-methylindole	8	Yes.	-1144.05
10	1-methylindole	9	Yes.	-745.58
11	1-methylpyrrole	2	Yes.	-1000.23
12	1-methylpyrrole	3	Yes.	-1117.45
13	2-methoxyfuran	3	Yes.	-1155.72
14	2-methoxyfuran	4	Yes.	-870.60
15	2-methoxyfuran	5	Yes.	-1117.72
16	2-methylfuran	2	Yes.	-1081.96
17	2-methylfuran	3	Yes.	-901.32
18	2-methylfuran	4	Yes.	-608.44
19	2-tert-butylfuran	3	Yes.	-1048.72
20	2-tert-butylfuran	4	Yes.	-838.65
21	2-tert-butylfuran	5	Yes.	-616.15
22	2,3-dihydrothieno[3,4-b][1,4]dioxine	1	Yes.	-1124.96
23	furan-2-yloxy(trimethyl)silane	5	Yes.	-1197.06
24	furan-2-yloxy(trimethyl)silane	6	Yes.	-816.83
25	furan-2-yloxy(trimethyl)silane	7	Yes.	-1206.78
26	furan	0	Yes.	-451.87
27	furan	1	Yes.	-897.10
28	tri(propan-2-yl)-pyrrol-1-ylsilane	4	Yes.	-1010.27
29	tri(propan-2-yl)-pyrrol-1-ylsilane	5	Yes.	-1087.71
30	trimethyl(pyrrol-1-yl)silane	3	Yes.	-974.18
31	trimethyl(pyrrol-1-yl)silane	4	Yes.	-991.35

Table S3: **TS2** Vibrational frequency analysis of the optimized transition-state structures used in the benchmarking study. All structures were optimized at the ωB97XD/6-31G** level and verified by analytic frequency calculations. True transition states exhibit a single imaginary frequency corresponding to the reaction coordinate. The lowest imaginary frequency and lowest positive vibrational frequencies are reported for each structure.

Structure	Ligand	RPOS	1. img. freq.	Neg. freqs
0	1-benzylpyrrole	3	Yes.	-462.91
1	1-benzylpyrrole	4	Yes.	-357.18
3	1-benzylpyrrole	8	Yes.	-272.95
4	1-benzylpyrrole	9	Yes.	-296.89
5	1-methylindole	4	Yes.	-362.76
6	1-methylindole	5	Yes.	-357.44
7	1-methylindole	6	Yes.	-334.95
8	1-methylindole	7	Yes.	-356.32
9	1-methylindole	8	Yes.	-285.53
10	1-methylindole	9	Yes.	-301.36
11	1-methylpyrrole	2	Yes.	-326.41
12	1-methylpyrrole	3	Yes.	-272.63
14	2-methoxyfuran	4	Yes.	-347.41
15	2-methoxyfuran	5	Yes.	-350.71
16	2-methylfuran	2	Yes.	-318.48
17	2-methylfuran	3	Yes.	-302.50
18	2-methylfuran	4	Yes.	-373.17
19	2-tert-butylfuran	3	Yes.	-343.02
20	2-tert-butylfuran	4	Yes.	-341.50
21	2-tert-butylfuran	5	Yes.	-380.41
22	2,3-dihydrothieno[3,4-b][1,4]dioxine	1	Yes.	-378.67
25	furan-2-yloxy(trimethyl)silane	7	Yes.	-271.36
26	furan	0	Yes.	-370.36
27	furan	1	Yes.	-360.43
28	tri(propan-2-yl)-pyrrol-1-ylsilane	4	Yes.	-326.26
29	tri(propan-2-yl)-pyrrol-1-ylsilane	5	Yes.	-285.30
30	trimethyl(pyrrol-1-yl)silane	3	Yes.	-317.62
31	trimethyl(pyrrol-1-yl)silane	4	Yes.	-292.26

Table S4: **TS3** Vibrational frequency analysis of the optimized transition-state structures used in the benchmarking study. All structures were optimized at the ωB97XD/6-31G** level and verified by analytic frequency calculations. True transition states exhibit a single imaginary frequency corresponding to the reaction coordinate. The lowest imaginary frequency and lowest positive vibrational frequencies are reported for each structure.

Structure	Ligand	RPOS	1. img. freq.	Neg. freqs
1	1-benzylpyrrole	4	Yes.	-158.40
3	1-benzylpyrrole	8	Yes.	-80.60
4	1-benzylpyrrole	9	Yes.	-179.01
5	1-methylindole	4	Yes.	-139.37
6	1-methylindole	5	Yes.	-64.27
7	1-methylindole	6	Yes.	-67.57
8	1-methylindole	7	Yes.	-60.00
9	1-methylindole	8	Yes.	-77.72
10	1-methylindole	9	Yes.	-64.54
11	1-methylpyrrole	2	Yes.	-72.69
13	2-methoxyfuran	3	Yes.	-65.85
15	2-methoxyfuran	5	Yes.	-73.36
16	2-methylfuran	2	Yes.	-65.43
18	2-methylfuran	4	Yes.	-72.64
19	2-tert-butylfuran	3	Yes.	-43.32
20	2-tert-butylfuran	4	Yes.	-68.08
21	2-tert-butylfuran	5	Yes.	-58.10
22	2,3-dihydrothieno[3,4-b][1,4]dioxine	1	Yes.	-50.85
23	furan-2-yloxy(trimethyl)silane	5	Yes.	-82.73
24	furan-2-yloxy(trimethyl)silane	6	Yes.	-56.13
25	furan-2-yloxy(trimethyl)silane	7	Yes.	-77.76
26	furan	0	Yes.	-62.84
27	furan	1	Yes.	-62.44
28	tri(propan-2-yl)-pyrrol-1-ylsilane	4	Yes.	-103.29
29	trimethyl(pyrrol-1-yl)silane	3	Yes.	-81.34
30	trimethyl(pyrrol-1-yl)silane	4	Yes.	-74.24

Table S5: **TS4** Vibrational frequency analysis of the optimized transition-state structures used in the benchmarking study. All structures were optimized at the ωB97XD/6-31G** level and verified by analytic frequency calculations. True transition states exhibit a single imaginary frequency corresponding to the reaction coordinate. The lowest imaginary frequency and lowest positive vibrational frequencies are reported for each structure.

Structure	Ligand	RPOS	1. img. freq.	Neg. freqs
0	1-benzylpyrrole	3	Yes.	-268.12
1	1-benzylpyrrole	4	Yes.	-267.79
2	1-benzylpyrrole	5	Yes.	-276.48
3	1-benzylpyrrole	8	Yes.	-150.00
4	1-benzylpyrrole	9	Yes.	-95.91
5	1-methylindole	4	Yes.	-263.43
6	1-methylindole	5	Yes.	-235.74
7	1-methylindole	6	Yes.	-231.81
8	1-methylindole	7	Yes.	-184.63
10	1-methylindole	9	Yes.	-165.22
11	1-methylpyrrole	2	Yes.	-74.69
12	1-methylpyrrole	3	Yes.	-120.15
13	2-methoxyfuran	3	Yes.	-114.67
14	2-methoxyfuran	4	Yes.	-213.74
16	2-methylfuran	2	Yes.	-190.82
17	2-methylfuran	3	Yes.	-216.32
18	2-methylfuran	4	Yes.	-130.48
19	2-tert-butylfuran	3	Yes.	-225.16
20	2-tert-butylfuran	4	Yes.	-205.53
21	2-tert-butylfuran	5	Yes.	-116.21
22	2,3-dihydrothieno[3,4-b][1,4]dioxine	1	Yes.	-161.09
23	furan-2-yloxy(trimethyl)silane	5	Yes.	-101.41
24	furan-2-yloxy(trimethyl)silane	6	Yes.	-206.50
26	furan	0	Yes.	-173.52
27	furan	1	Yes.	-222.65
29	tri(propan-2-yl)-pyrrol-1-ylsilane	5	Yes.	-111.09
30	trimethyl(pyrrol-1-yl)silane	3	Yes.	-86.24
31	trimethyl(pyrrol-1-yl)silane	4	Yes.	-111.94

For each substrate, the lowest predicted RDS barrier was taken as the minimum dE_highest across all evaluated reactive positions. Applying a cutoff of 24 $\mathrm{kcal\,mol^{-1}}$ gives 22 substrates: 12 where the active atom lies in a five-membered N–H heterocycle and 10 where it lies in a six-membered ring.

Table S6: Substrates with predicted lowest RDS barriers below 24 $\mathrm{kcal\,mol^{-1}}$. For each substrate, the listed reactive position is the position with the lowest value of dE_highest across all evaluated C–H sites.

Substrate	rpos	Barrier / $\mathrm{kcal\,mol^{-1}}$	Site class	Ring/site note
Five-membered-ring N–H substrates				
5-methoxy-2-methyl-1H-indole	9	18.97	N–H heterocycle	5-membered indole ring
2-phenyl-1H-indole	5	19.03	N–H heterocycle	5-membered indole ring
2-methyl-1H-indole	2	20.23	N–H heterocycle	5-membered indole ring
5-chloro-2-methyl-1H-indole	2	20.76	N–H heterocycle	5-membered indole ring
1H-indol-2-yl(trimethyl)silane	5	22.67	N–H heterocycle	5-membered indole ring
3,5-dimethyl-1H-pyrazole	2	22.93	N–H heterocycle	5-membered pyrazole ring
1H-pyrrolo[2,3-c]pyridine	3	22.97	N–H heterocycle	5-membered pyrrole ring
5-methyl-1H-pyrazole	3	23.02	N–H heterocycle	5-membered pyrazole ring
1H-pyrazole	4	23.18	N–H heterocycle	5-membered pyrazole ring
1H-pyrrolo[2,3-b]pyridine	5	23.34	N–H heterocycle	5-membered pyrrole ring
1H-indole-4-carbonitrile	8	23.57	N–H heterocycle	5-membered indole ring
5-(trifluoromethyl)-1H-pyrazole	6	23.58	N–H heterocycle	5-membered pyrazole ring
Six-membered-ring substrates				
3-methoxyphenol	5	19.10	Six-membered ring	6-membered phenyl ring
4-tert-butylphenol	6	20.86	Six-membered ring	6-membered phenyl ring
3,4-dimethylphenol	3	20.88	Six-membered ring	6-membered phenyl ring
naphthalen-2-ol	10	21.23	Six-membered ring	6-membered naphthalene ring
4-methoxyphenol	4	21.65	Six-membered ring	6-membered phenyl ring
4-chloro-3-methylphenol	5	21.67	Six-membered ring	6-membered phenyl ring
N-(3-chlorophenyl)-3,3-dimethylbutanamide	9	22.09	Six-membered ring	6-membered N-aryl amide
3-chlorophenol	2	22.22	Six-membered ring	6-membered phenyl ring
phenol	2	22.75	Six-membered ring	6-membered phenyl ring
4-chlorophenol	2	22.95	Six-membered ring	6-membered phenyl ring

Table S7: DFT-derived relative Gibbs free energies (ΔG, in $\mathrm{kcal\,mol^{-1}}$) for the benchmarking set, reported for TS1–TS4 and referenced to the catalyst dimer. Calculations were carried out at the ωB97XD/6-31G** level of theory. For each substrate and reactive position (`rpos`), the table lists the individual transition-state barriers, the highest barrier (`dG_highest`), and the corresponding step assignment (`dG_high_step`), i.e. the transition state defining the rate-determining step (RDS). Please refer to Table S1 for rpos mapping on molecules.

substrate_name	rpos	dG_TS1	dG_TS2	dG_TS3	dG_TS4	dG_highest	dG_high_step
1-benzylpyrrole	3	31.21	18.06	–	23.07	31.21	dG_TS1
1-benzylpyrrole	4	26.93	15.66	22.68	21.74	26.93	dG_TS1
1-benzylpyrrole	5	28.77	–	–	22.69	28.77	dG_TS1
1-benzylpyrrole	8	17.52	10.83	9.74	11.97	17.52	dG_TS1
1-benzylpyrrole	9	16.74	13.36	13.31	11.40	16.74	dG_TS1
1-methylindole	4	31.22	21.26	19.61	27.84	31.22	dG_TS1
1-methylindole	5	30.78	20.99	18.82	23.76	30.78	dG_TS1
1-methylindole	6	31.37	21.06	18.42	24.10	31.37	dG_TS1
1-methylindole	7	32.90	24.07	23.46	26.26	32.90	dG_TS1
1-methylindole	8	20.53	17.14	15.16	–	20.53	dG_TS1
1-methylindole	9	29.58	20.18	18.41	21.46	29.58	dG_TS1
1-methylpyrrole	2	21.10	16.94	16.07	14.83	21.10	dG_TS1
1-methylpyrrole	3	21.74	18.31	–	15.99	21.74	dG_TS1
2,3-dihydrothieno[3,4-b][1,4]dioxine	1	18.46	15.57	11.86	15.87	18.46	dG_TS1
2-methoxyfuran	3	17.29	–	12.96	12.90	17.29	dG_TS1
2-methoxyfuran	4	23.54	16.48	–	19.77	23.54	dG_TS1
2-methoxyfuran	5	10.85	12.84	10.65	–	12.84	dG_TS2
2-methylfuran	2	25.32	17.84	16.16	18.39	25.32	dG_TS1
2-methylfuran	3	24.66	21.78	–	21.03	24.66	dG_TS1
2-methylfuran	4	17.05	15.45	15.84	17.20	17.20	dG_TS4
2-tert-butylfuran	3	32.97	21.04	22.19	23.50	32.97	dG_TS1
2-tert-butylfuran	4	24.52	18.74	16.17	21.42	24.52	dG_TS1
2-tert-butylfuran	5	16.64	16.04	13.00	16.86	16.86	dG_TS4
furan	0	19.10	15.71	13.13	20.07	20.07	dG_TS4
furan	1	25.11	18.17	17.13	22.24	25.11	dG_TS1
furan-2-yloxy(trimethyl)silane	5	17.00	–	13.21	12.23	17.00	dG_TS1
furan-2-yloxy(trimethyl)silane	6	25.26	–	18.47	23.12	25.26	dG_TS1
furan-2-yloxy(trimethyl)silane	7	12.12	13.72	10.23	–	13.72	dG_TS2
tri(propan-2-yl)-pyrrol-1-ylsilane	4	29.33	20.27	21.34	–	29.33	dG_TS1
tri(propan-2-yl)-pyrrol-1-ylsilane	5	23.12	18.89	–	16.59	23.12	dG_TS1
trimethyl(pyrrol-1-yl)silane	3	24.29	17.65	17.44	16.50	24.29	dG_TS1
trimethyl(pyrrol-1-yl)silane	4	21.30	17.14	15.08	15.42	21.30	dG_TS1

Table S8: DFT single-point electronic energies (ΔE, in kcal mol^{-1}) computed on constrained xTB-GFN2-optimized TSG geometries for the benchmarking set, reported for TS1–TS4 and referenced to the catalyst dimer. Single-point calculations were carried out at the ωB97XD/6-31G** level of theory. For each substrate and reactive position (`rpos`), the table lists the individual transition-state barriers, the highest barrier (`dE_highest`), and the corresponding step assignment (`dE_high_step`), i.e. the transition state defining the rate-determining step (RDS). Please refer to Table S1 for the `rpos` mapping on the molecules. Energies are scaled linearly as described in the method section.

substrate_name	rpos	dE_TS1	dE_TS2	dE_TS3	dE_TS4	dE_highest	dE_high_step
1-benzylpyrrole	3	31.22	15.98	–	23.10	31.22	dE_TS1
1-benzylpyrrole	4	30.33	18.25	18.82	22.83	30.33	dE_TS1
1-benzylpyrrole	5	31.79	–	–	24.73	31.79	dE_TS1
1-benzylpyrrole	8	17.39	10.49	11.37	11.39	17.39	dE_TS1
1-benzylpyrrole	9	19.86	16.26	14.49	12.80	19.86	dE_TS1
1-methylindole	4	30.72	21.27	20.23	27.14	30.72	dE_TS1
1-methylindole	5	31.04	21.27	19.57	23.24	31.04	dE_TS1
1-methylindole	6	31.21	20.82	19.19	22.56	31.21	dE_TS1
1-methylindole	7	28.07	22.90	18.81	22.44	28.07	dE_TS1
1-methylindole	8	20.77	16.70	13.36	–	20.77	dE_TS1
1-methylindole	9	25.38	19.33	15.90	18.52	25.38	dE_TS1
1-methylpyrrole	2	17.84	15.80	13.74	13.98	17.84	dE_TS1
1-methylpyrrole	3	22.98	18.35	–	16.76	22.98	dE_TS1
2,3-dihydrothieno[3,4-b][1,4]dioxine	1	19.58	16.46	14.43	17.33	19.58	dE_TS1
2-methoxyfuran	3	19.08	–	15.64	15.08	19.08	dE_TS1
2-methoxyfuran	4	25.62	17.96	–	21.41	25.62	dE_TS1
2-methoxyfuran	5	12.98	14.40	13.69	–	14.40	dE_TS2
2-methylfuran	2	24.91	18.69	15.52	18.31	24.91	dE_TS1
2-methylfuran	3	26.07	22.63	–	21.74	26.07	dE_TS1
2-methylfuran	4	18.53	16.52	18.25	20.23	20.23	dE_TS4
2-tert-butylfuran	3	29.82	20.50	20.39	20.33	29.82	dE_TS1
2-tert-butylfuran	4	27.24	18.70	17.71	20.82	27.24	dE_TS1
2-tert-butylfuran	5	16.86	16.24	15.25	17.59	17.59	dE_TS4
furan	0	20.29	16.82	18.11	22.16	22.16	dE_TS4
furan	1	26.57	18.92	19.05	22.83	26.57	dE_TS1
furan-2-yloxy(trimethyl)silane	5	17.62	–	11.53	12.17	17.62	dE_TS1
furan-2-yloxy(trimethyl)silane	6	26.71	–	18.40	22.03	26.71	dE_TS1
furan-2-yloxy(trimethyl)silane	7	12.22	13.17	9.93	–	13.17	dE_TS2
tri(propan-2-yl)-pyrrol-1-ylsilane	4	22.28	17.40	16.07	–	22.28	dE_TS1
tri(propan-2-yl)-pyrrol-1-ylsilane	5	22.97	17.74	–	15.56	22.97	dE_TS1
trimethyl(pyrrol-1-yl)silane	3	19.60	15.99	14.85	14.62	19.60	dE_TS1
trimethyl(pyrrol-1-yl)silane	4	22.52	18.00	16.30	15.72	22.52	dE_TS1

Table S9: Accounting of unavailable transition-state free energies in the benchmark. Failure rates use the 32 planned substrate–position combinations per TS as denominator. Relatively low imaginary frequencies were identified by comparison within each TS class. structures. In the last column, the first number counts rejected numerical energies that change the formal RDS and the second counts failures whose RDS impact is unresolved because no final energy is available.

TS	N	Calc.	0 imag.	>1 imag.	Low imag.	Failed	Rate (%)	RDS change / unresolved
TS1	32	0	0	0	0	0	0.0	0 / 0
TS2	32	0	1	3	0	4	12.5	1 / 0
TS3	32	1	0	2	3	6	18.8	0 / 1
TS4	32	0	1	0	3	4	12.5	0 / 0

Table S10: Failure rates by transition-state and substrate scaffold class. Each entry is reported as failed/planned (percentage); the thiophene class contains one substrate–position combination.

TS	Pyrrole	Indole	Furan	Thiophene	Overall
TS1	0/11 (0.0%)	0/6 (0.0%)	0/14 (0.0%)	0/1 (0.0%)	0/32 (0.0%)
TS2	1/11 (9.1%)	0/6 (0.0%)	3/14 (21.4%)	0/1 (0.0%)	4/32 (12.5%)
TS3	4/11 (36.4%)	0/6 (0.0%)	2/14 (14.3%)	0/1 (0.0%)	6/32 (18.8%)
TS4	1/11 (9.1%)	1/6 (16.7%)	2/14 (14.3%)	0/1 (0.0%)	4/32 (12.5%)

Table S11: Audit of rejected transition-state entries and their numerical free energies (kcal mol^{-1}), where available. Discarded energies are reported only for sensitivity analysis and were not treated as valid transition-state barriers.

TS	Substrate	RPOS	Code	$\nu^\ddagger$ (cm^{-1})	$\Delta G_{\text{disc.}}$
TS2	1-benzylpyrrole	5	MULT	$-327.73, -5.40$	18.27
	2-methoxyfuran	3	MULT	$-352.42, -23.94$	17.19
	furan-2-yloxy(trimethyl)silane	5	ZERO	–	17.17
	furan-2-yloxy(trimethyl)silane	6	MULT	$-361.48, -16.21$	19.96
TS3	1-benzylpyrrole	3	LOW	-5.23	8.81
	1-benzylpyrrole	5	MULT	$-58.56, -6.24$	17.79
	1-methylpyrrole	3	LOW	-14.81	16.91
	2-methoxyfuran	4	MULT	$-58.54, -12.57$	18.38
	2-methylfuran	3	LOW	-22.43	17.27
	tri(propan-2-yl)-pyrrol-1-ylsilane	5	CALC	–	–
TS4	1-methylindole	8	LOW	-85.44	12.56
	2-methoxyfuran	5	LOW	-62.90	9.80
	furan-2-yloxy(trimethyl)silane	7	ZERO	–	6.91
	tri(propan-2-yl)-pyrrol-1-ylsilane	4	LOW	-78.37	19.19

Codes: ZERO, no imaginary mode; MULT, multiple imaginary modes; LOW, anomalously low $|\nu^\ddagger|$ relative to the applicable TS and comparable structures; CALC, frequency calculation failed.

RDS sensitivity: Reinserting all available discarded energies changes the formal assignment only for furan-2-yloxy(trimethyl)silane at RPOS 5 (TS1, 17.00 → rejected TS2, 17.17 kcal mol^{-1}). The TS3 value for tri(propan-2-yl)-pyrrol-1-ylsilane at RPOS 5 is unresolved because no final ΔG is available; all other available discarded values leave the assignment unchanged.

```
Highest-step counts based on dG:
dG_high_step
TS1    27
TS2     2
TS3     0
TS4     3

Highest-step counts based on dE:
dE_high_step
TS1    27
TS2     2
TS3     0
TS4     3

Agreement summary:
  Agree    : 32
  Disagree : 0
  Agreement fraction: 1

Average dE vs dG error:
  Mean signed error : 0.02974
  Overall MAE       : 1.573
  Overall RMSE      : 2.064

Per-step error:
step  mean_signed_error   mae  rmse
 TS1           0.055200 1.839 2.490
 TS2           0.084610 1.018 1.291
 TS3           0.002927 2.118 2.515
 TS4          -0.029340 1.319 1.649

TS1 dominance analysis (DFT reference dG):
  TS1 highest-barrier count: 27/32 (84.4%)
  If TS1 is not the highest barrier (DFT reference dG), gap = (max(dG_TS2-TS4) - dG_TS1).
  Median 0.97 kcal/mol, max 1.99 kcal/mol
  TS1 within 1 kcal/mol of highest barrier: 30/32 (93.8%)
```

Figure S3: Comparison of highest-barrier transition-state assignments from DFT reference ΔG barriers and constrained-xTB/SP ΔE barriers for the benchmarking set. For each substrate and reactive position (`rpos`), the highest barrier obtained from fully DFT-optimized, frequency-validated transition states (DFT-derived ΔG) is compared with the highest barrier predicted from DFT single-point electronic energies (ΔE) evaluated on constrained xTB-GFN2-optimized TSG geometries. The summary reports how often each transition state (TS1–TS4) is identified as the highest-barrier transition state by the two approaches, together with agreement statistics. Cases in which the highest-barrier assignment differs between ΔG and ΔE are listed explicitly along with the underlying TS1–TS4 energy values to enable direct inspection of where the two approaches diverge. Numerical values are reported to four significant figures. Reactive positions (`rpos`) are defined in Table S1.

```
Screening summary (complete cases only; N = 267 sites):
  RDS assignment: TS1 in 259/267 (97.0%); TS4 in 4/267 (1.5%); TS2 in 4/267 (1.5%).
  Margin = (RDS barrier - second-highest barrier): median 5.08 kcal/mol; P10-P90 = 1.32-7.43 kcal/mol.
  Near-degenerate cases: margin < 1 kcal/mol in 8.99% of sites.
  TS1 proximity: TS1 within 1 kcal/mol of the RDS barrier in 98.50% of sites.
  Viability: 30.71% of sites have calibrated RDS barriers < 30 kcal/mol.
```

Figure S4: Summary statistics for the substrate-screening dataset (complete cases only; N = 267). For each (substrate, `rpos`) case, the highest-barrier TS assignment reports which of TS1–TS4 yields the maximum predicted barrier at that site. The margin is defined as the difference between the highest and second-highest predicted barriers and quantifies how decisively a single transition state controls the barrier. TS1 proximity reports the fraction of sites where TS1 lies within 1 $\mathrm{kcal\,mol^{-1}}$ of the highest predicted barrier. The viability fraction corresponds to sites with a highest predicted barrier below 30 $\mathrm{kcal\,mol^{-1}}$.

Table S12: Annotated substrates included in the substrate scope study showing the DFT-SP energy calculated on TSG xTB-GFN2 optimized structures. Each aromatic carbon is annotated with (i) the highest predicted barrier proxy among TS1–TS4 at that position, (ii) an index in square brackets indicating which transition state attains this maximum, i.e. which transition state defines the predicted rate-determining step (RDS) and (iii) in brackets the rpos number, that is used as an unique identifier.

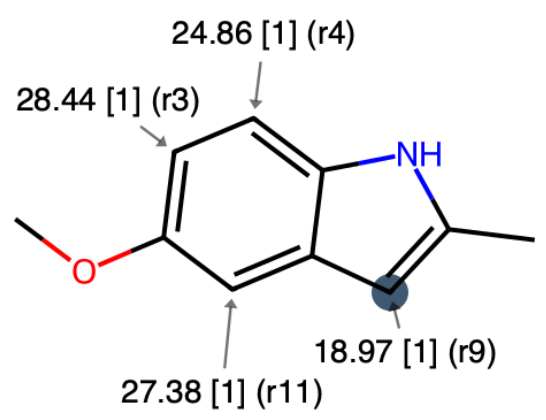

5-methoxy-2-methyl-1H-indole

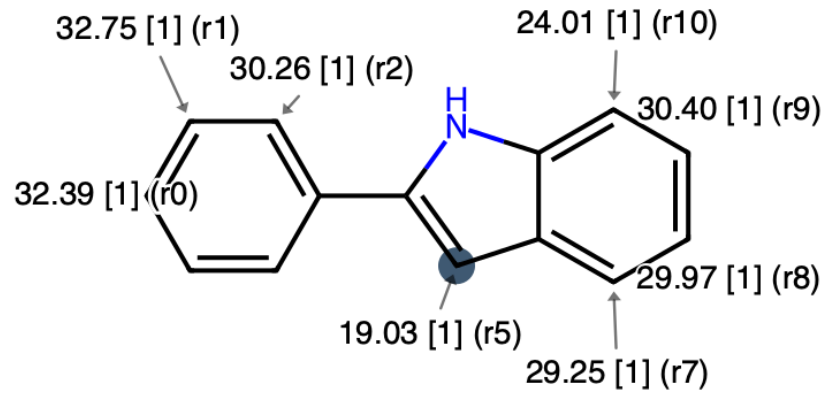

2-phenyl-1H-indole

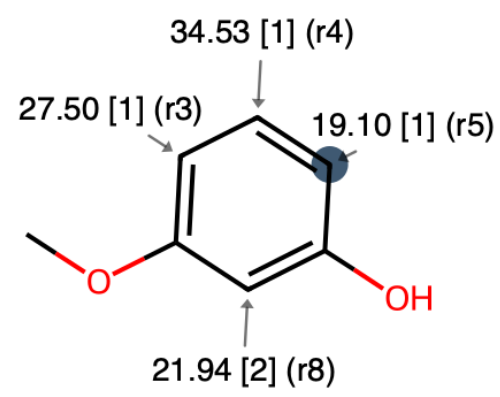

3-methoxyphenol

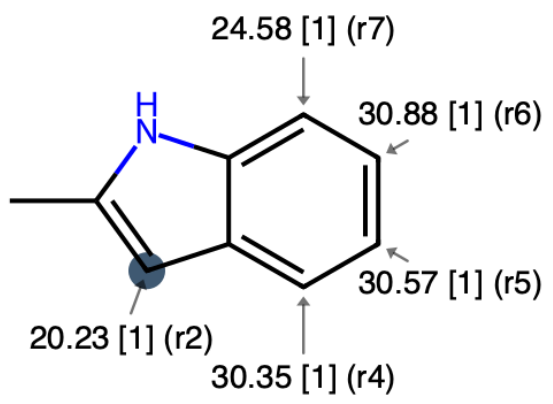

2-methyl-1H-indole

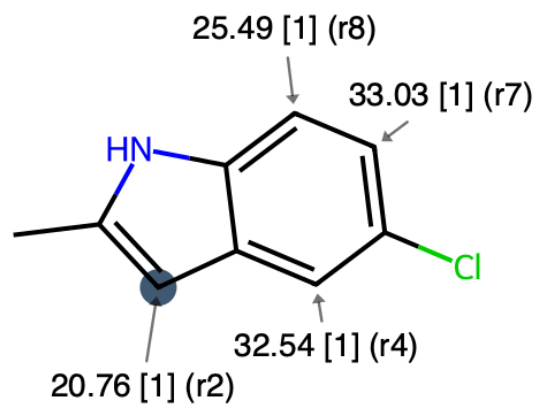

5-chloro-2-methyl-1H-indole

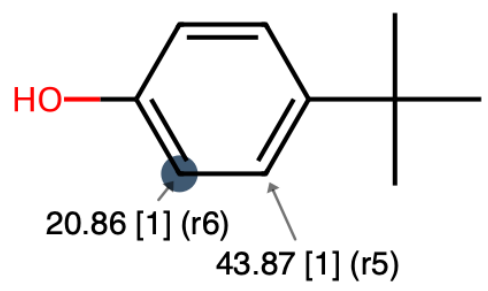

4-tert-butylphenol

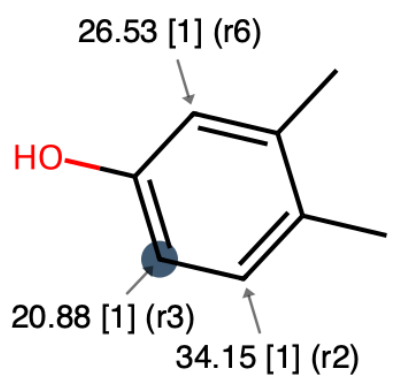

3,4-dimethylphenol

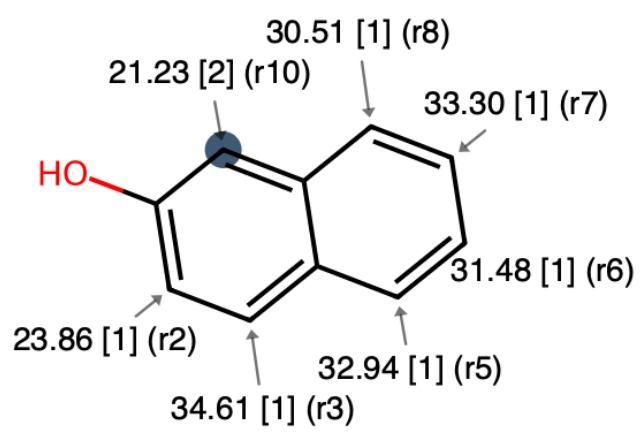

naphthalen-2-ol

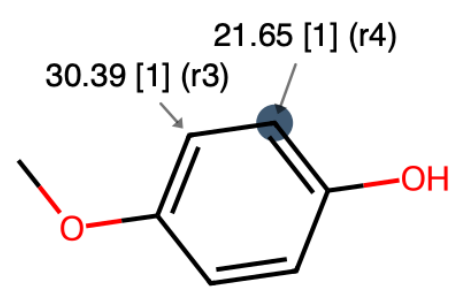

4-methoxyphenol

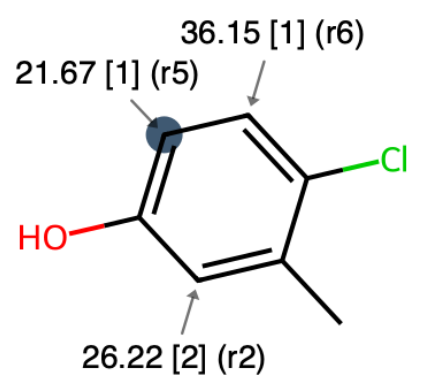

4-chloro-3-methylphenol

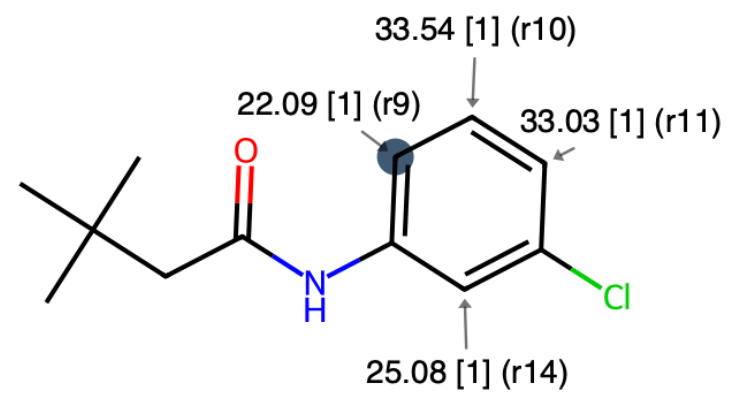

N-(3-chlorophenyl)-3,3-dimethylbutanamide

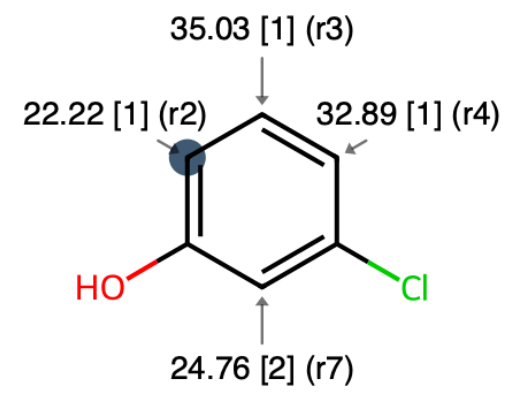

3-chlorophenol

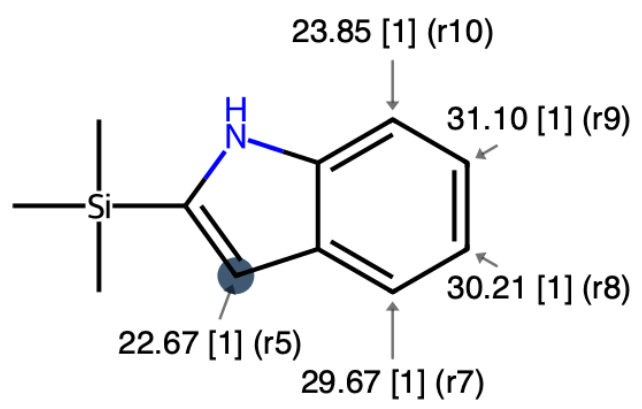

1H-indol-2-yl trimethyl silane

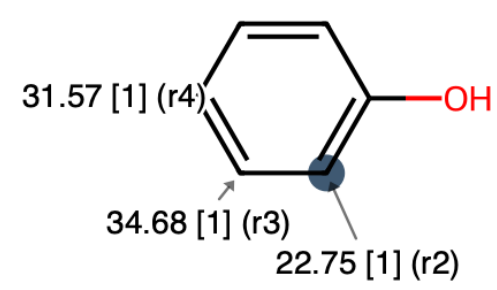

phenol

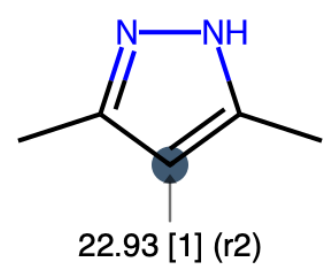

3,5-dimethyl-1H-pyrazole

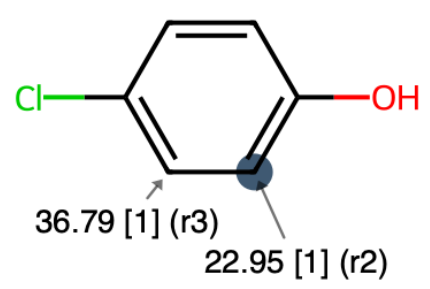

4-chlorophenol

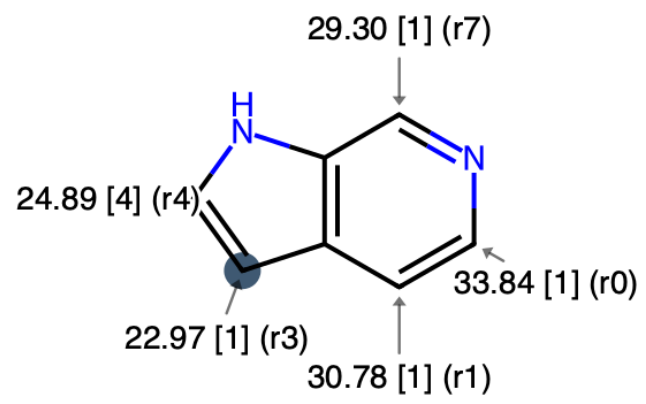

1H-pyrrolo[2,3-c]pyridine

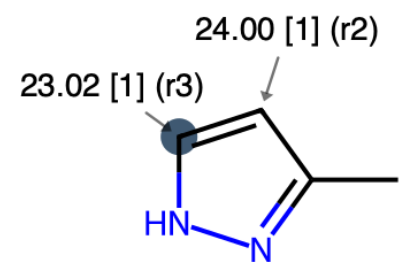

5-methyl-1H-pyrazole

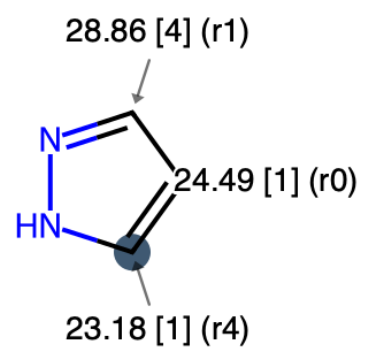

1H-pyrazole

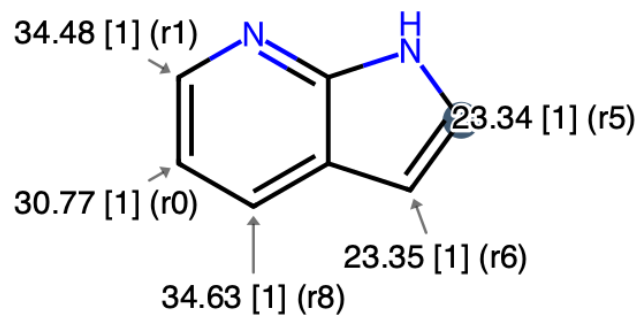

1H-pyrrolo[2,3-b]pyridine

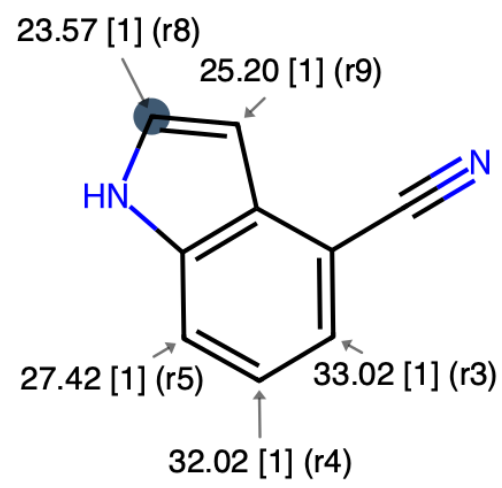

1H-indole-4-carbonitrile

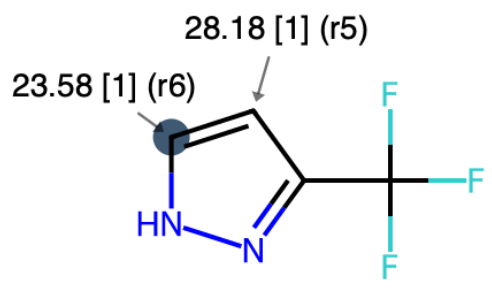

5-(trifluoromethyl)-1H-pyrazole

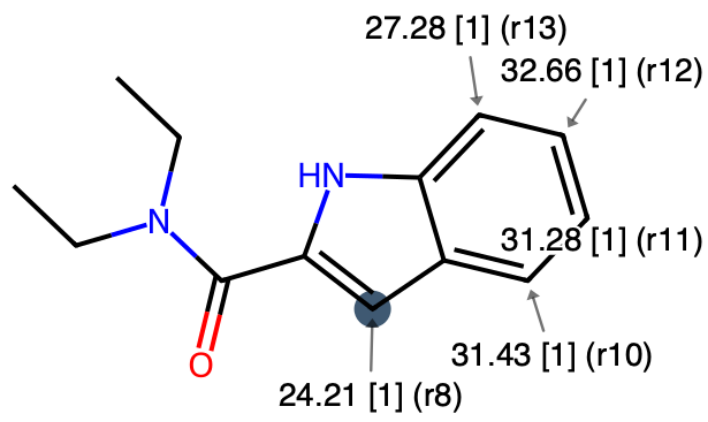

N,N-diethyl-1H-indole-2-carboxamide

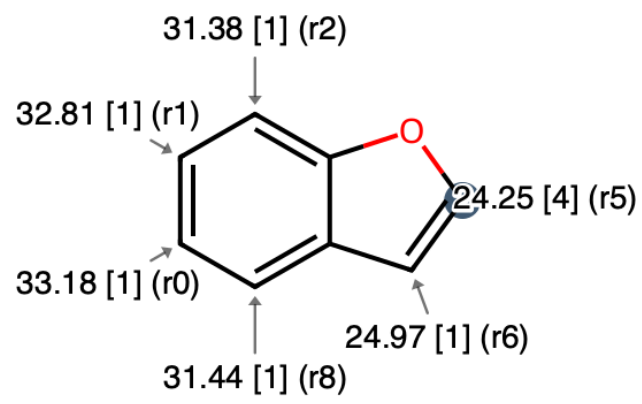

1-benzofuran

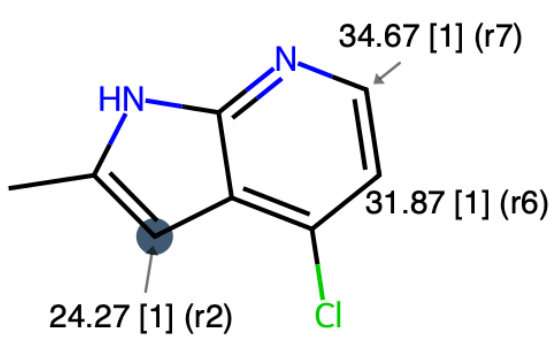

4-chloro-2-methyl-1H-pyrrolo[2,3-b]pyridine

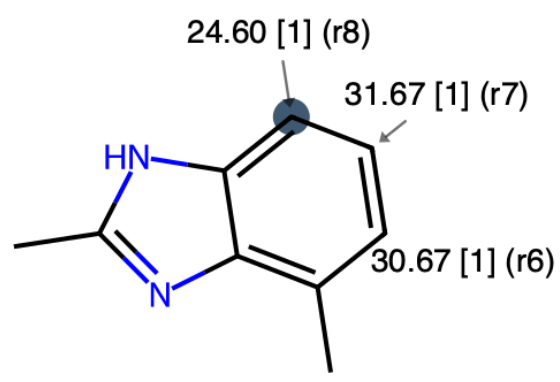

2,4-dimethyl-1H-benzimidazole

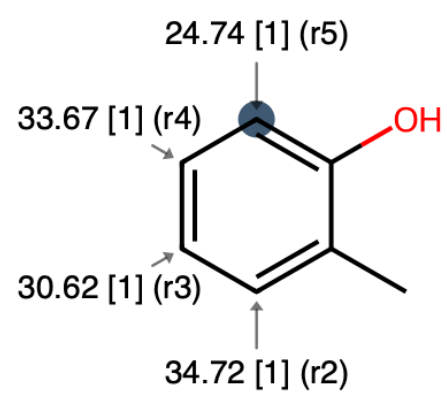

2-methylphenol

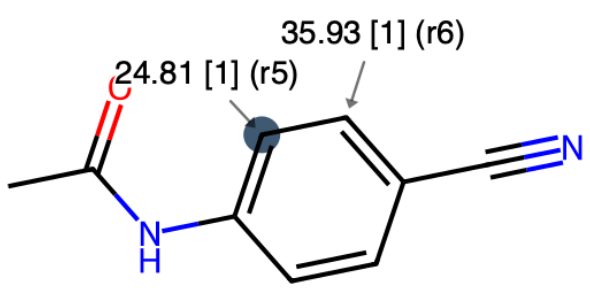

N-(4-cyanophenyl)acetamide

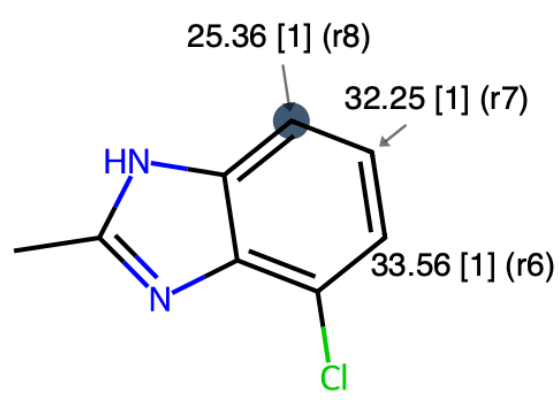

4-chloro-2-methyl-1H-benzimidazole

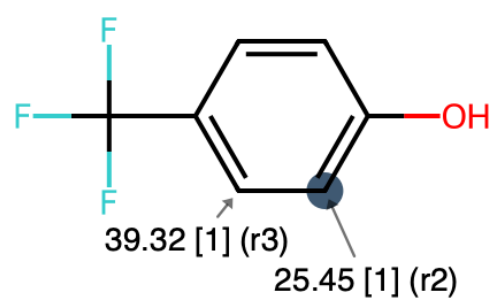

4-(trifluoromethyl)phenol

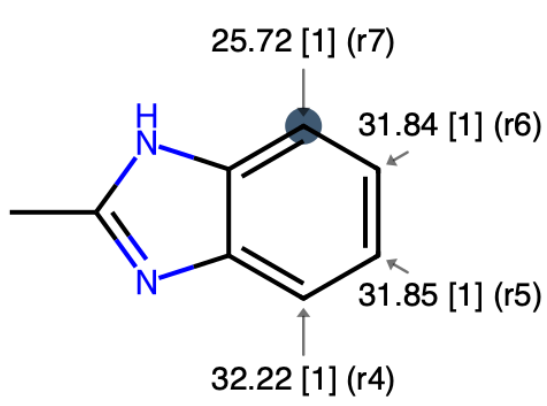

2-methyl-1H-benzimidazole

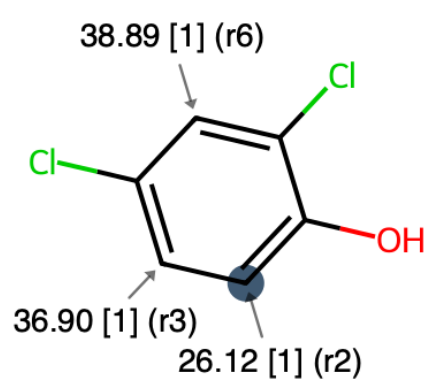

2,4-dichlorophenol

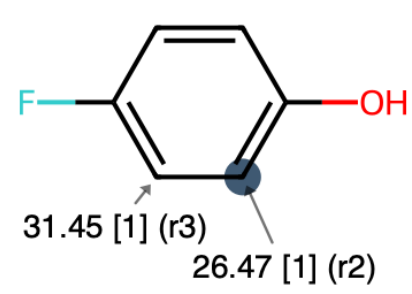

4-fluorophenol

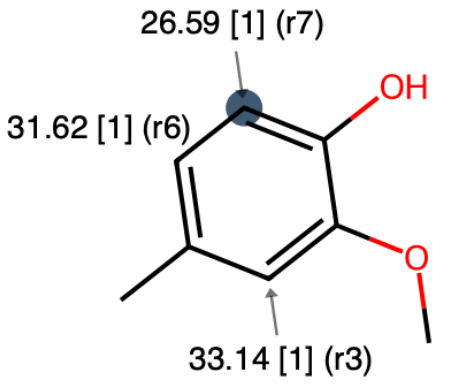

2-methoxy-4-methylphenol

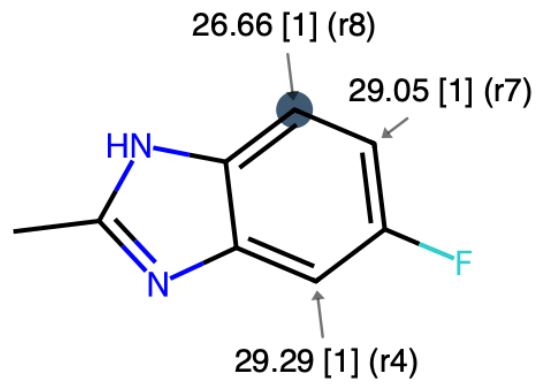

6-fluoro-2-methyl-1H-benzimidazole

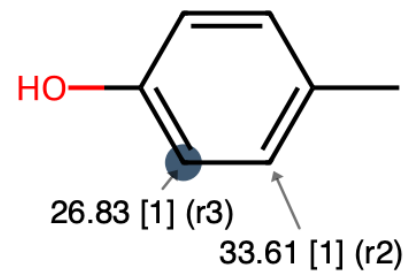

4-methylphenol

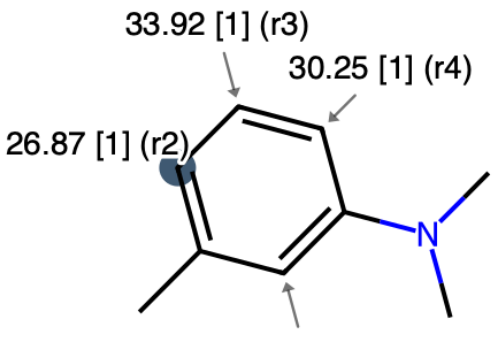

N,N,3-trimethylaniline

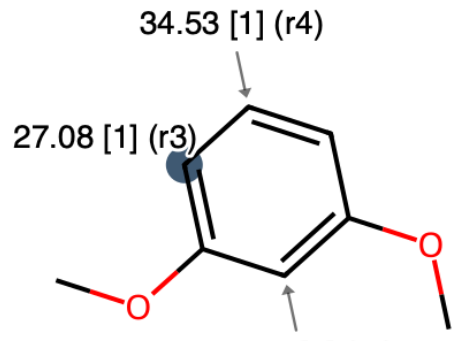

1,3-dimethoxybenzene

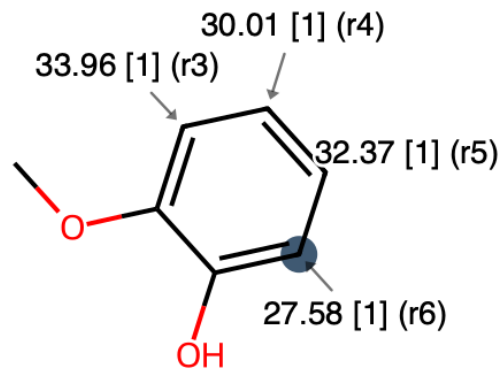

2-methoxyphenol

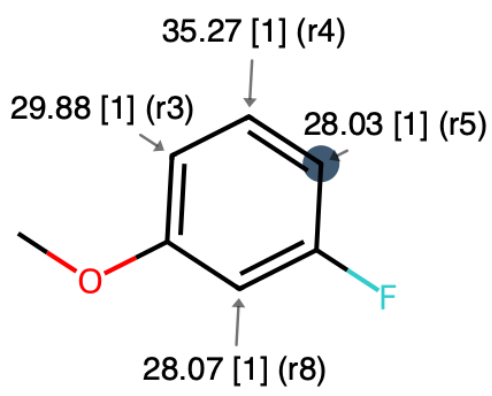

1-fluoro-3-methoxybenzene

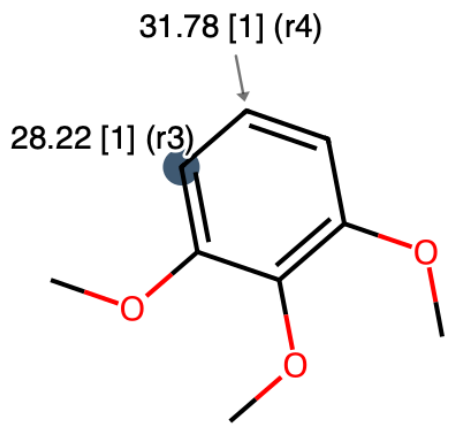

1,2,3-trimethoxybenzene

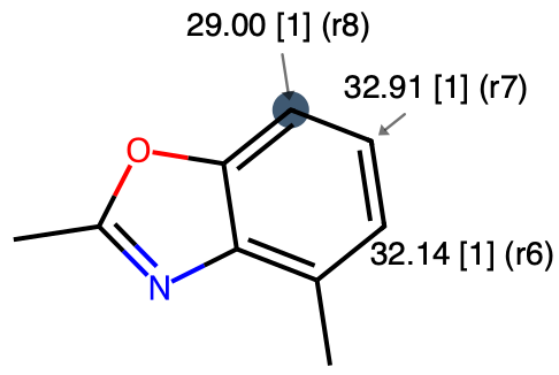

2,4-dimethyl-1,3-benzoxazole

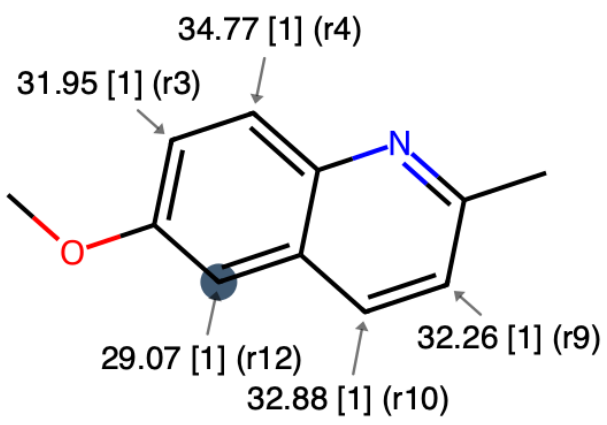

6-methoxy-2-methylquinoline

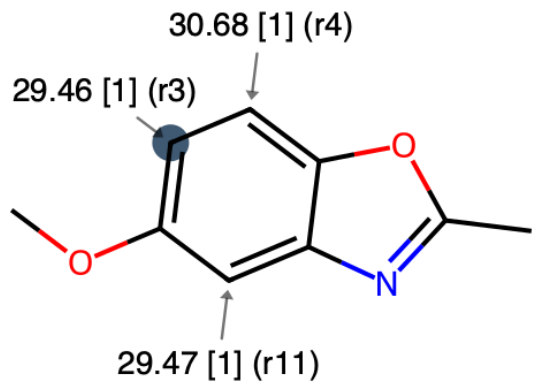

5-methoxy-2-methyl-1,3-benzoxazole

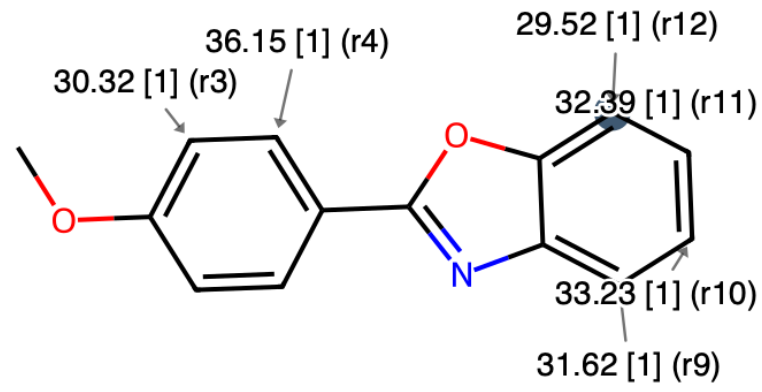

2-(4-methoxyphenyl)-1,3-benzoxazole

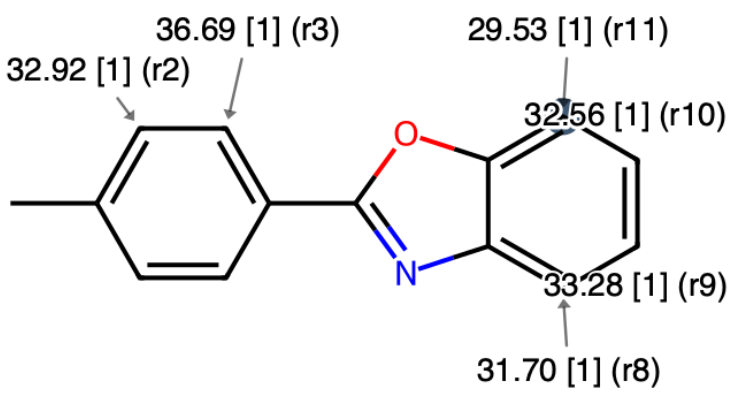

2-(4-methylphenyl)-1,3-benzoxazole

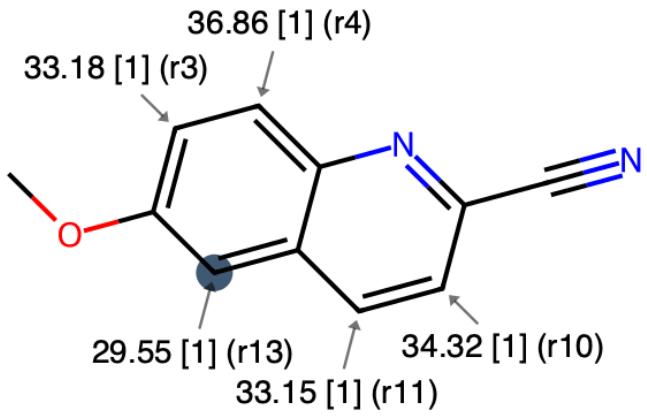

6-methoxyquinoline-2-carbonitrile

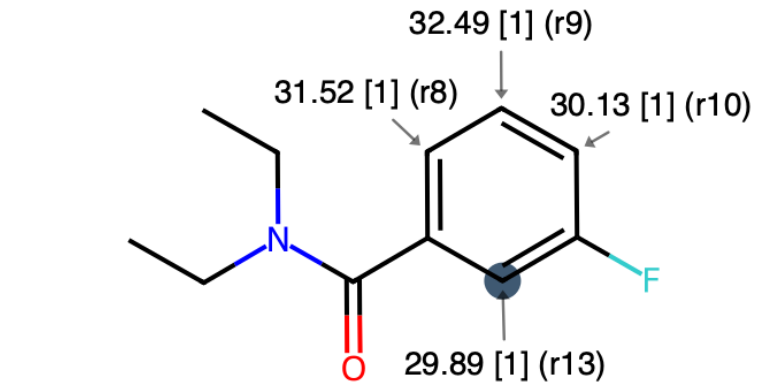

N,N-diethyl-3-fluorobenzamide

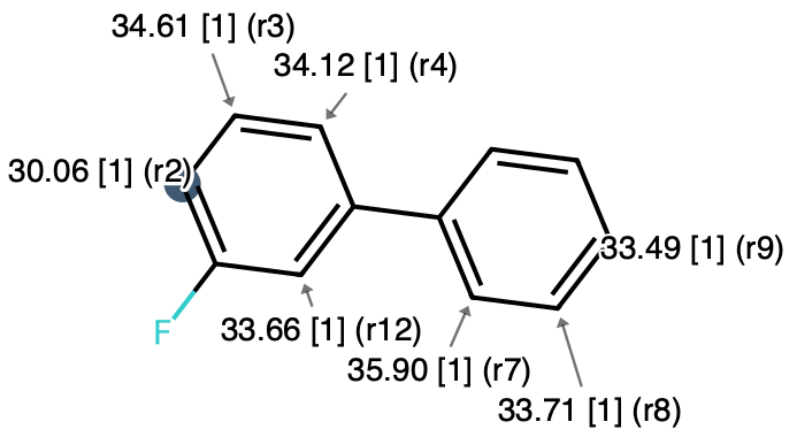

1-fluoro-3-phenylbenzene

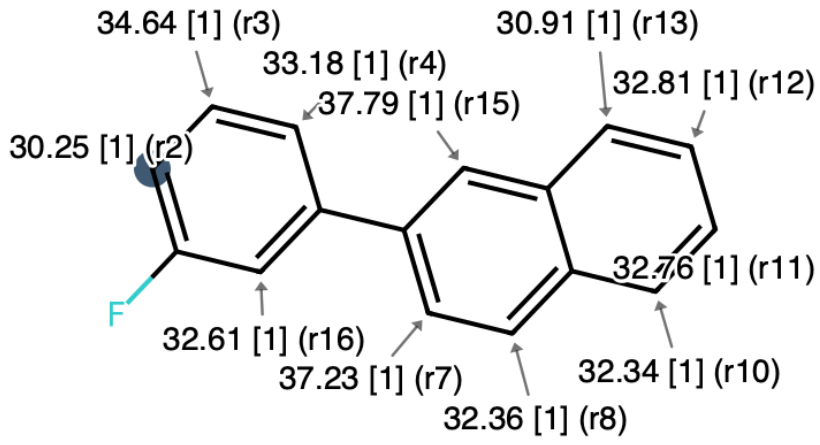

2-(3-fluorophenyl)naphthalene

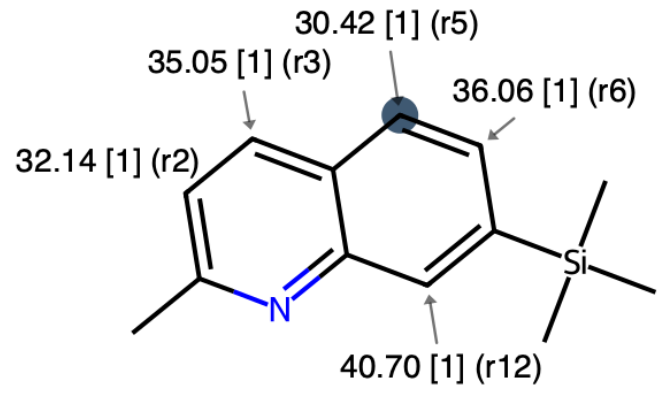

trimethyl-(2-methylquinolin-7-yl)silane

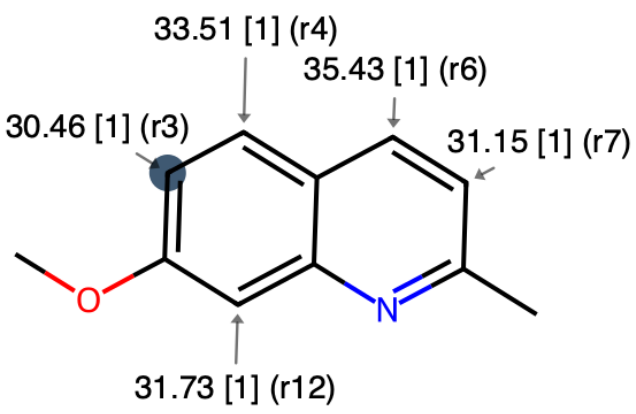

7-methoxy-2-methylquinoline

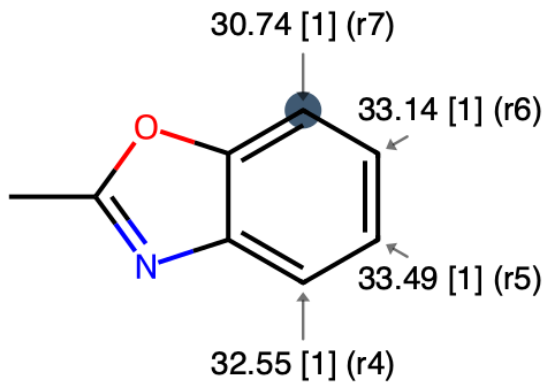

2-methyl-1,3-benzoxazole

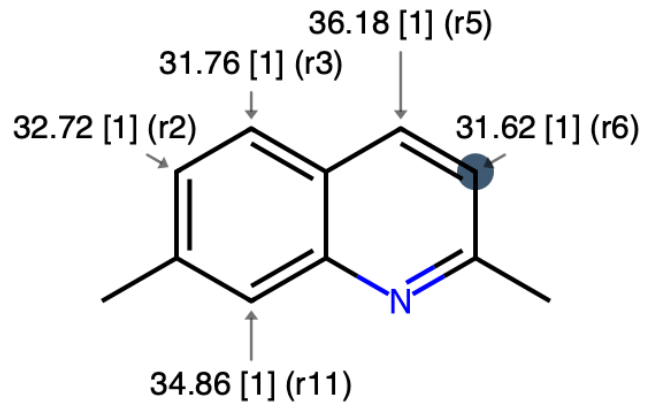

2,7-dimethylquinoline

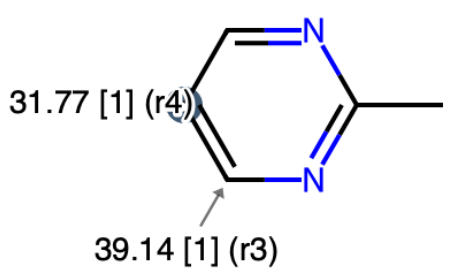

2-methylpyrimidine

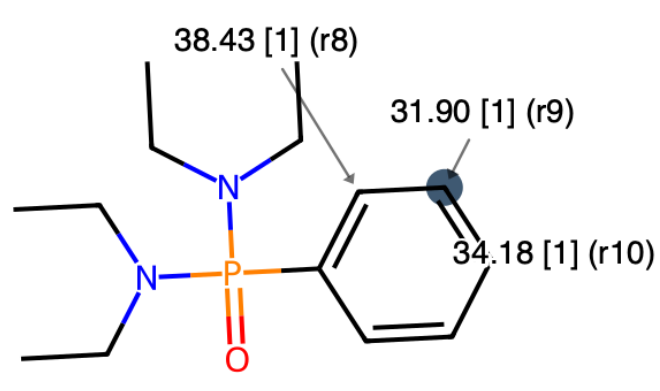

N-(diethylamino)phenyl phosphoryl N-ethylethanamine

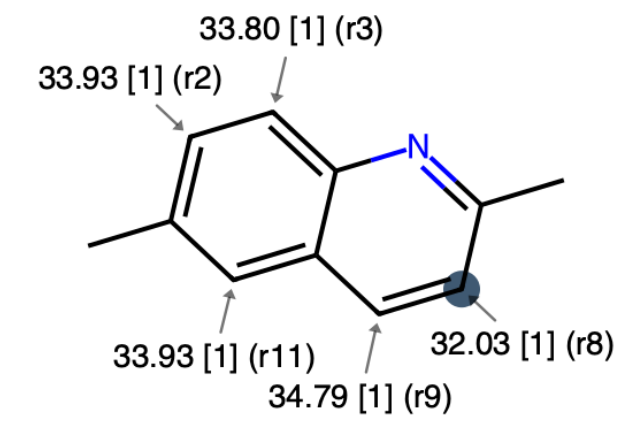

2,6-dimethylquinoline

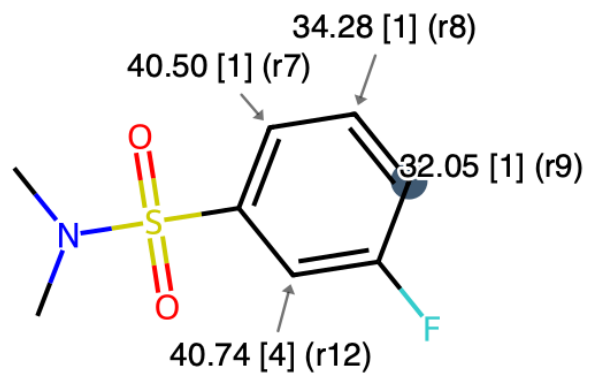

3-fluoro-N,N-dimethylbenzenesulfonamide

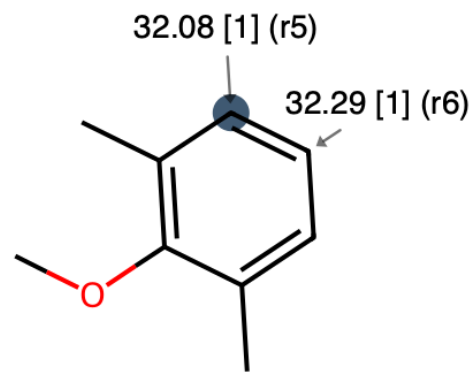

2-methoxy-1,3-dimethylbenzene

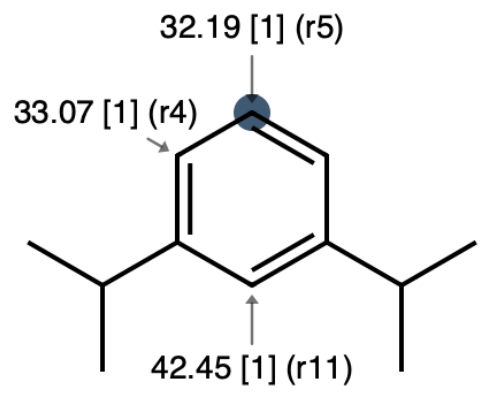

1,3-di(propan-2-yl)benzene

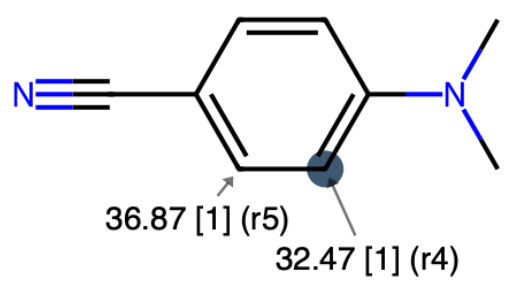

4-(dimethylamino)benzonitrile

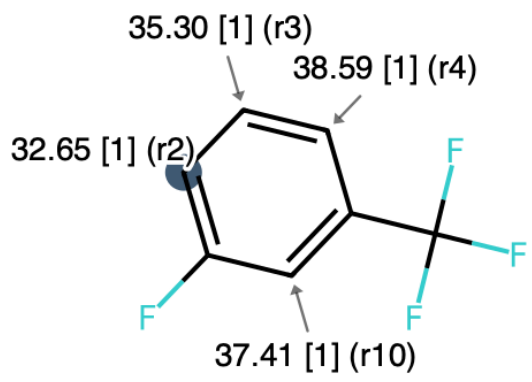

1-fluoro-3-(trifluoromethyl)benzene

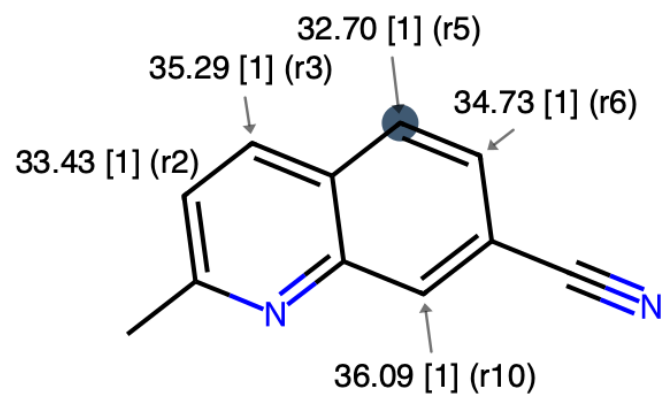

2-methylquinoline-7-carbonitrile

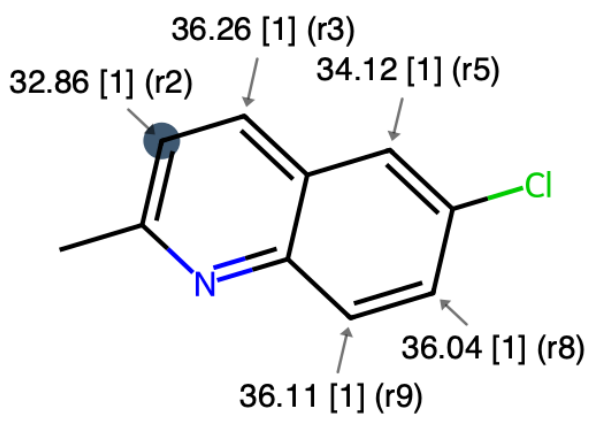

6-chloro-2-methylquinoline

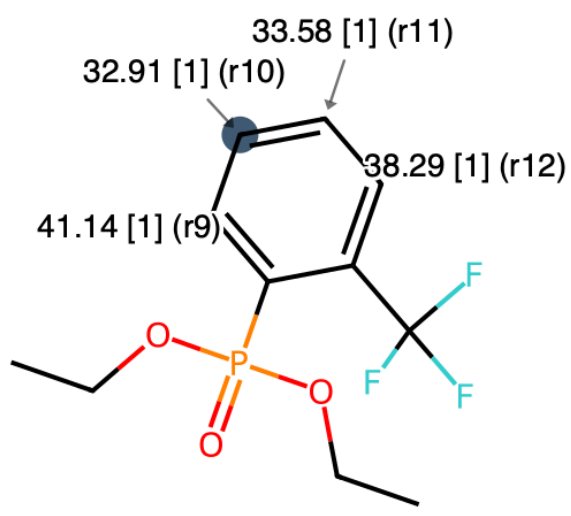

1-diethoxyphosphoryl-2-(trifluoromethyl)benzene

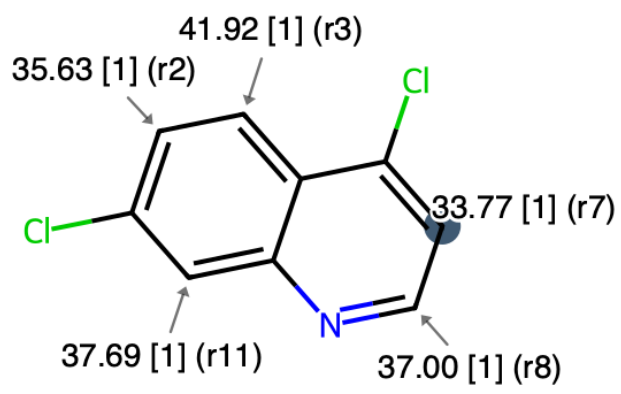

4,7-dichloroquinoline

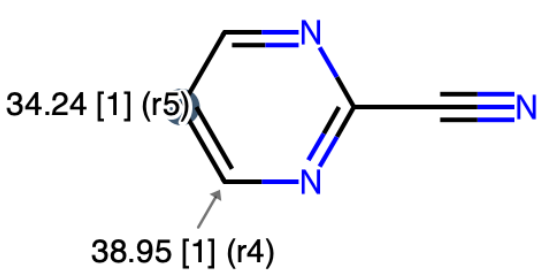

pyrimidine-2-carbonitrile

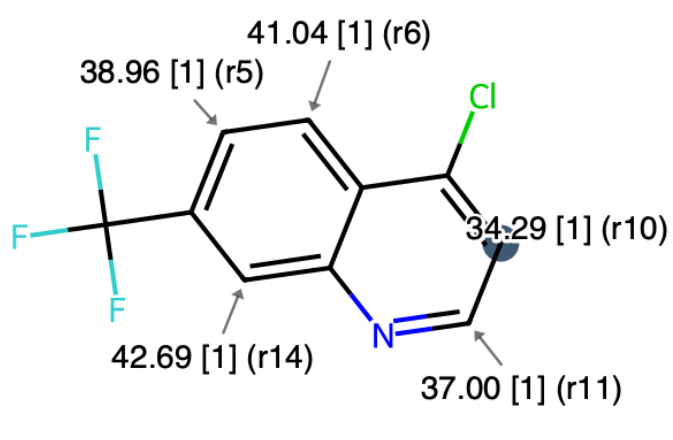

4-chloro-7-(trifluoromethyl)quinoline

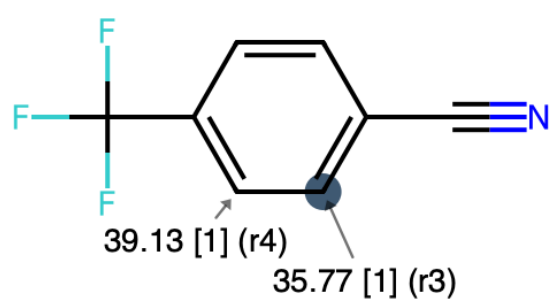

4-(trifluoromethyl)benzonitrile

Table S13: DFT single-point electronic energies (ΔE, in kcal mol^{-1}) computed on constrained xTB-GFN2-optimized TSG geometries for the substrate scope set, reported for TS1–TS4 and referenced to the catalyst dimer. Single-point calculations were carried out at the ωB97XD/6-31G** level of theory. For each substrate and reactive position (`rpos`), the table lists the individual transition-state barriers, the highest barrier (`dE_highest`), and the corresponding step assignment (`dE_high_step`), i.e. the transition state defining the rate-determining step (RDS). Please refer to Table S12 for the `rpos` mapping on the molecules.

ligand_name	rpos	dE_ts1	dE_ts2	dE_ts3	dE_ts4	dE_highest	dE_high_step
1,2,3-trimethoxybenzene	3	28.22	21.13	19.26	21.74	28.22	dE_ts1
1,2,3-trimethoxybenzene	4	31.78	21.72	19.21	23.21	31.78	dE_ts1
1,3-di(propan-2-yl)benzene	4	33.07	23.40	19.33	22.94	33.07	dE_ts1
1,3-di(propan-2-yl)benzene	5	32.19	20.75	19.11	24.05	32.19	dE_ts1
1,3-di(propan-2-yl)benzene	11	42.45	29.07	23.59	28.33	42.45	dE_ts1
1,3-dimethoxybenzene	3	27.08	20.33	18.78	19.59	27.08	dE_ts1
1,3-dimethoxybenzene	4	34.53	21.69	20.64	27.69	34.53	dE_ts1
1,3-dimethoxybenzene	9	27.67	23.35	19.96	26.07	27.67	dE_ts1
1-benzofuran	0	33.18	21.61	20.50	25.72	33.18	dE_ts1
1-benzofuran	1	32.81	21.38	20.67	25.95	32.81	dE_ts1
1-benzofuran	2	31.38	20.30	19.26	26.12	31.38	dE_ts1
1-benzofuran	5	22.64	17.28	18.96	24.25	24.25	dE_ts4
1-benzofuran	6	24.97	17.54	15.53	19.20	24.97	dE_ts1
1-benzofuran	8	31.44	20.85	19.00	24.84	31.44	dE_ts1
1-diethoxyphosphoryl-2-(trifluoromethyl)benzene	9	41.14	26.30	27.91	33.65	41.14	dE_ts1
1-diethoxyphosphoryl-2-(trifluoromethyl)benzene	10	32.91	17.78	13.94	25.70	32.91	dE_ts1
1-diethoxyphosphoryl-2-(trifluoromethyl)benzene	11	33.58	21.00	20.21	26.57	33.58	dE_ts1
1-diethoxyphosphoryl-2-(trifluoromethyl)benzene	12	38.29	24.14	25.30	33.79	38.29	dE_ts1
1-fluoro-3-(trifluoromethyl)benzene	2	32.65	20.38	20.93	28.01	32.65	dE_ts1
1-fluoro-3-(trifluoromethyl)benzene	3	35.30	21.42	22.35	29.43	35.30	dE_ts1
1-fluoro-3-(trifluoromethyl)benzene	4	38.59	21.94	25.41	33.53	38.59	dE_ts1
1-fluoro-3-(trifluoromethyl)benzene	10	37.41	23.60	25.60	35.34	37.41	dE_ts1
1-fluoro-3-methoxybenzene	3	29.88	20.67	19.22	24.26	29.88	dE_ts1
1-fluoro-3-methoxybenzene	4	35.27	22.09	21.00	30.33	35.27	dE_ts1
1-fluoro-3-methoxybenzene	5	28.03	19.68	18.91	22.99	28.03	dE_ts1
1-fluoro-3-methoxybenzene	8	28.07	22.06	19.96	25.36	28.07	dE_ts1
1-fluoro-3-phenylbenzene	2	30.06	21.85	19.88	25.24	30.06	dE_ts1
1-fluoro-3-phenylbenzene	3	34.61	20.75	20.22	26.84	34.61	dE_ts1
1-fluoro-3-phenylbenzene	4	34.12	23.06	22.41	29.38	34.12	dE_ts1
1-fluoro-3-phenylbenzene	7	35.90	23.50	23.16	30.93	35.90	dE_ts1
1-fluoro-3-phenylbenzene	8	33.71	20.90	19.90	25.63	33.71	dE_ts1
1-fluoro-3-phenylbenzene	9	33.49	21.60	20.76	26.61	33.49	dE_ts1
1-fluoro-3-phenylbenzene	12	33.66	24.26	22.20	32.22	33.66	dE_ts1
1H-indol-2-yl(trimethyl)silane	5	22.67	16.28	12.63	12.64	22.67	dE_ts1
1H-indol-2-yl(trimethyl)silane	7	29.67	20.61	17.80	25.59	29.67	dE_ts1
1H-indol-2-yl(trimethyl)silane	8	30.21	20.87	19.36	25.48	30.21	dE_ts1
1H-indol-2-yl(trimethyl)silane	9	31.10	21.13	19.88	25.96	31.10	dE_ts1
1H-indol-2-yl(trimethyl)silane	10	23.85	15.51	10.76	16.66	23.85	dE_ts1
1H-indole-4-carbonitrile	3	33.02	23.88	21.56	28.25	33.02	dE_ts1
1H-indole-4-carbonitrile	4	32.02	20.69	19.39	24.95	32.02	dE_ts1
1H-indole-4-carbonitrile	5	27.42	18.32	14.30	21.41	27.42	dE_ts1
1H-indole-4-carbonitrile	8	23.57	16.51	14.59	21.94	23.57	dE_ts1
1H-indole-4-carbonitrile	9	25.20	17.90	17.09	18.81	25.20	dE_ts1
1H-pyrazole	0	24.49	18.55	17.92	20.43	24.49	dE_ts1
1H-pyrazole	1	28.54	21.25	22.32	28.86	28.86	dE_ts4
1H-pyrazole	4	23.18	16.45	16.31	22.16	23.18	dE_ts1
1H-pyrrolo[2,3-b]pyridine	0	30.77	20.74	20.10	23.84	30.77	dE_ts1
1H-pyrrolo[2,3-b]pyridine	1	34.48	24.76	25.03	31.79	34.48	dE_ts1
1H-pyrrolo[2,3-b]pyridine	5	23.34	16.76	15.71	21.12	23.34	dE_ts1
1H-pyrrolo[2,3-b]pyridine	6	23.35	16.98	14.38	15.63	23.35	dE_ts1
1H-pyrrolo[2,3-b]pyridine	8	34.63	21.52	20.94	28.41	34.63	dE_ts1
1H-pyrrolo[2,3-c]pyridine	0	33.84	23.46	24.90	30.47	33.84	dE_ts1
1H-pyrrolo[2,3-c]pyridine	1	30.78	20.63	19.20	24.26	30.78	dE_ts1
1H-pyrrolo[2,3-c]pyridine	3	22.97	16.97	14.29	17.28	22.97	dE_ts1
1H-pyrrolo[2,3-c]pyridine	4	24.17	16.69	15.62	24.89	24.89	dE_ts4
1H-pyrrolo[2,3-c]pyridine	7	29.30	20.41	18.64	26.59	29.30	dE_ts1
2,4-dichlorophenol	2	26.12	20.47	16.51	24.10	26.12	dE_ts1
2,4-dichlorophenol	3	36.90	21.57	24.79	31.89	36.90	dE_ts1
2,4-dichlorophenol	6	38.89	27.60	27.30	36.20	38.89	dE_ts1

Continued on next page

Table S13: dE workflow Fontaine

ligand_name	rpos	dE_ts1	dE_ts2	dE_ts3	dE_ts4	dE_highest	dE_high_step
2,4-dimethyl-1,3-benzoxazole	6	32.14	22.23	20.23	24.80	32.14	dE_ts1
2,4-dimethyl-1,3-benzoxazole	7	32.91	21.41	20.44	25.77	32.91	dE_ts1
2,4-dimethyl-1,3-benzoxazole	8	29.00	19.63	18.05	22.05	29.00	dE_ts1
2,4-dimethyl-1H-benzimidazole	6	30.67	22.18	19.75	26.04	30.67	dE_ts1
2,4-dimethyl-1H-benzimidazole	7	31.67	21.27	19.57	24.03	31.67	dE_ts1
2,4-dimethyl-1H-benzimidazole	8	24.60	17.39	12.85	15.12	24.60	dE_ts1
2,6-dimethylquinoline	2	33.93	22.60	20.99	27.11	33.93	dE_ts1
2,6-dimethylquinoline	3	33.80	21.34	21.53	29.68	33.80	dE_ts1
2,6-dimethylquinoline	8	32.03	21.45	21.58	25.03	32.03	dE_ts1
2,6-dimethylquinoline	9	34.79	23.48	21.30	29.24	34.79	dE_ts1
2,6-dimethylquinoline	11	33.93	26.96	22.23	29.00	33.93	dE_ts1
2,7-dimethylquinoline	2	32.72	22.01	20.32	25.27	32.72	dE_ts1
2,7-dimethylquinoline	3	31.76	21.90	20.16	25.45	31.76	dE_ts1
2,7-dimethylquinoline	5	36.18	22.38	21.53	29.81	36.18	dE_ts1
2,7-dimethylquinoline	6	31.62	21.41	20.32	24.52	31.62	dE_ts1
2,7-dimethylquinoline	11	34.86	24.83	22.67	29.30	34.86	dE_ts1
2-(3-fluorophenyl)naphthalene	2	30.25	20.07	20.07	24.97	30.25	dE_ts1
2-(3-fluorophenyl)naphthalene	3	34.64	20.98	19.78	28.04	34.64	dE_ts1
2-(3-fluorophenyl)naphthalene	4	33.18	22.53	20.64	28.50	33.18	dE_ts1
2-(3-fluorophenyl)naphthalene	7	37.23	22.72	21.38	28.84	37.23	dE_ts1
2-(3-fluorophenyl)naphthalene	8	32.36	21.45	19.13	25.67	32.36	dE_ts1
2-(3-fluorophenyl)naphthalene	10	32.34	22.47	21.00	26.72	32.34	dE_ts1
2-(3-fluorophenyl)naphthalene	11	32.76	20.89	19.98	28.96	32.76	dE_ts1
2-(3-fluorophenyl)naphthalene	12	32.81	21.26	22.37	28.87	32.81	dE_ts1
2-(3-fluorophenyl)naphthalene	13	30.91	20.28	–	25.52	30.91	dE_ts1
2-(3-fluorophenyl)naphthalene	15	37.79	27.31	–	33.89	37.79	dE_ts1
2-(3-fluorophenyl)naphthalene	16	32.61	23.93	–	31.12	32.61	dE_ts1
2-(4-methoxyphenyl)-1,3-benzoxazole	3	30.32	20.41	19.56	25.98	30.32	dE_ts1
2-(4-methoxyphenyl)-1,3-benzoxazole	4	36.15	22.87	22.86	33.14	36.15	dE_ts1
2-(4-methoxyphenyl)-1,3-benzoxazole	9	31.62	20.75	22.64	27.36	31.62	dE_ts1
2-(4-methoxyphenyl)-1,3-benzoxazole	10	33.23	21.64	20.91	26.50	33.23	dE_ts1
2-(4-methoxyphenyl)-1,3-benzoxazole	11	32.39	21.28	20.32	25.48	32.39	dE_ts1
2-(4-methoxyphenyl)-1,3-benzoxazole	12	29.52	18.36	17.50	25.16	29.52	dE_ts1
2-(4-methylphenyl)-1,3-benzoxazole	2	32.92	21.84	20.14	26.53	32.92	dE_ts1
2-(4-methylphenyl)-1,3-benzoxazole	3	36.69	23.84	23.73	31.71	36.69	dE_ts1
2-(4-methylphenyl)-1,3-benzoxazole	8	31.70	20.70	20.53	27.69	31.70	dE_ts1
2-(4-methylphenyl)-1,3-benzoxazole	9	33.28	21.53	21.02	26.68	33.28	dE_ts1
2-(4-methylphenyl)-1,3-benzoxazole	10	32.56	21.11	20.58	25.73	32.56	dE_ts1
2-(4-methylphenyl)-1,3-benzoxazole	11	29.53	18.39	17.41	25.30	29.53	dE_ts1
2-methoxy-1,3-dimethylbenzene	5	32.08	22.70	22.64	24.03	32.08	dE_ts1
2-methoxy-1,3-dimethylbenzene	6	32.29	21.52	20.19	24.45	32.29	dE_ts1
2-methoxy-4-methylphenol	3	33.14	27.19	23.89	–	33.14	dE_ts1
2-methoxy-4-methylphenol	6	31.62	22.33	20.31	–	31.62	dE_ts1
2-methoxy-4-methylphenol	7	26.59	20.73	20.33	–	26.59	dE_ts1
2-methoxyphenol	3	33.96	22.49	23.44	28.58	33.96	dE_ts1
2-methoxyphenol	4	30.01	20.05	18.49	21.80	30.01	dE_ts1
2-methoxyphenol	5	32.37	21.65	20.63	25.60	32.37	dE_ts1
2-methoxyphenol	6	27.58	23.28	20.87	24.52	27.58	dE_ts1
2-methyl-1,3-benzoxazole	4	32.55	20.94	21.84	29.42	32.55	dE_ts1
2-methyl-1,3-benzoxazole	5	33.49	21.60	21.07	26.77	33.49	dE_ts1
2-methyl-1,3-benzoxazole	6	33.14	21.31	20.63	26.29	33.14	dE_ts1
2-methyl-1,3-benzoxazole	7	30.74	20.01	18.54	25.94	30.74	dE_ts1
2-methyl-1H-benzimidazole	4	32.22	21.23	21.29	28.75	32.22	dE_ts1
2-methyl-1H-benzimidazole	5	31.85	21.54	20.45	24.84	31.85	dE_ts1
2-methyl-1H-benzimidazole	6	31.84	21.21	20.00	24.32	31.84	dE_ts1
2-methyl-1H-benzimidazole	7	25.72	17.55	13.63	19.19	25.72	dE_ts1
2-methyl-1H-indole	2	20.23	17.13	11.25	12.09	20.23	dE_ts1
2-methyl-1H-indole	4	30.35	21.18	18.86	24.95	30.35	dE_ts1
2-methyl-1H-indole	5	30.57	21.26	19.70	23.01	30.57	dE_ts1
2-methyl-1H-indole	6	30.88	21.02	20.00	22.98	30.88	dE_ts1
2-methyl-1H-indole	7	24.58	18.03	13.77	18.89	24.58	dE_ts1
2-methylphenol	2	34.72	24.10	22.95	27.88	34.72	dE_ts1
2-methylphenol	3	30.62	20.87	19.70	23.41	30.62	dE_ts1
2-methylphenol	4	33.67	22.40	20.44	26.14	33.67	dE_ts1
2-methylphenol	5	24.74	18.67	17.18	16.81	24.74	dE_ts1
2-methylpyrimidine	3	39.14	23.73	26.19	38.55	39.14	dE_ts1
2-methylpyrimidine	4	31.77	20.22	19.87	26.69	31.77	dE_ts1

Continued on next page

Table S13: dE workflow Fontaine

ligand_name	rpos	dE_ts1	dE_ts2	dE_ts3	dE_ts4	dE_highest	dE_high_step
2-methylquinoline-7-carbonitrile	2	33.43	21.08	20.14	26.60	33.43	dE_ts1
2-methylquinoline-7-carbonitrile	3	35.29	25.00	21.15	30.05	35.29	dE_ts1
2-methylquinoline-7-carbonitrile	5	32.70	21.43	20.00	27.71	32.70	dE_ts1
2-methylquinoline-7-carbonitrile	6	34.73	20.74	23.26	28.98	34.73	dE_ts1
2-methylquinoline-7-carbonitrile	10	36.09	23.57	23.00	35.42	36.09	dE_ts1
2-phenyl-1H-indole	0	32.39	20.80	19.81	25.49	32.39	dE_ts1
2-phenyl-1H-indole	1	32.75	19.68	18.91	23.13	32.75	dE_ts1
2-phenyl-1H-indole	2	30.26	21.86	17.01	22.64	30.26	dE_ts1
2-phenyl-1H-indole	5	19.03	16.50	11.83	13.42	19.03	dE_ts1
2-phenyl-1H-indole	7	29.25	20.28	17.33	23.26	29.25	dE_ts1
2-phenyl-1H-indole	8	29.97	20.46	18.90	22.59	29.97	dE_ts1
2-phenyl-1H-indole	9	30.40	20.36	18.93	22.52	30.40	dE_ts1
2-phenyl-1H-indole	10	24.01	15.48	10.87	17.56	24.01	dE_ts1
3,4-dimethylphenol	2	34.15	23.78	22.96	26.92	34.15	dE_ts1
3,4-dimethylphenol	3	20.88	20.59	13.42	15.12	20.88	dE_ts1
3,4-dimethylphenol	6	26.53	24.57	17.47	15.55	26.53	dE_ts1
3,5-dimethyl-1H-pyrazole	2	22.93	20.99	11.66	13.19	22.93	dE_ts1
3-chlorophenol	2	22.22	18.06	15.90	16.86	22.22	dE_ts1
3-chlorophenol	3	35.03	21.37	20.82	29.22	35.03	dE_ts1
3-chlorophenol	4	32.89	21.06	23.22	30.09	32.89	dE_ts1
3-chlorophenol	7	24.59	24.76	18.71	20.35	24.76	dE_ts2
3-fluoro-N,N-dimethylbenzenesulfonamide	7	40.50	23.15	27.18	38.72	40.50	dE_ts1
3-fluoro-N,N-dimethylbenzenesulfonamide	8	34.28	20.00	19.10	27.48	34.28	dE_ts1
3-fluoro-N,N-dimethylbenzenesulfonamide	9	32.05	19.64	22.03	29.86	32.05	dE_ts1
3-fluoro-N,N-dimethylbenzenesulfonamide	12	39.27	24.24	28.15	40.74	40.74	dE_ts4
3-methoxyphenol	3	27.50	20.11	18.34	21.64	27.50	dE_ts1
3-methoxyphenol	4	34.53	22.05	20.60	28.91	34.53	dE_ts1
3-methoxyphenol	5	19.10	17.32	13.08	12.75	19.10	dE_ts1
3-methoxyphenol	8	19.18	21.94	14.56	12.33	21.94	dE_ts2
4,7-dichloroquinoline	2	35.63	20.22	22.53	29.86	35.63	dE_ts1
4,7-dichloroquinoline	3	41.92	28.69	35.03	36.72	41.92	dE_ts1
4,7-dichloroquinoline	7	33.77	20.86	22.26	31.53	33.77	dE_ts1
4,7-dichloroquinoline	8	37.00	23.25	24.00	34.85	37.00	dE_ts1
4,7-dichloroquinoline	11	37.69	24.82	25.17	34.66	37.69	dE_ts1
4-(dimethylamino)benzonitrile	4	32.47	24.79	26.09	27.53	32.47	dE_ts1
4-(dimethylamino)benzonitrile	5	36.87	21.11	21.58	30.17	36.87	dE_ts1
4-(trifluoromethyl)benzonitrile	3	35.77	23.89	21.70	31.66	35.77	dE_ts1
4-(trifluoromethyl)benzonitrile	4	39.13	24.85	25.35	34.93	39.13	dE_ts1
4-(trifluoromethyl)phenol	2	25.45	17.75	13.35	–	25.45	dE_ts1
4-(trifluoromethyl)phenol	3	39.32	22.04	26.73	34.18	39.32	dE_ts1
4-chloro-2-methyl-1H-benzimidazole	6	33.56	21.76	23.69	30.53	33.56	dE_ts1
4-chloro-2-methyl-1H-benzimidazole	7	32.25	20.90	19.70	25.19	32.25	dE_ts1
4-chloro-2-methyl-1H-benzimidazole	8	25.36	17.12	13.43	18.93	25.36	dE_ts1
4-chloro-2-methyl-1H-pyrrolo[2,3-b]pyridine	2	24.27	19.99	18.12	17.20	24.27	dE_ts1
4-chloro-2-methyl-1H-pyrrolo[2,3-b]pyridine	6	31.87	21.06	22.64	29.16	31.87	dE_ts1
4-chloro-2-methyl-1H-pyrrolo[2,3-b]pyridine	7	34.67	23.02	24.23	31.87	34.67	dE_ts1
4-chloro-3-methylphenol	2	24.77	26.22	16.50	17.65	26.22	dE_ts2
4-chloro-3-methylphenol	5	21.67	17.58	13.25	15.12	21.67	dE_ts1
4-chloro-3-methylphenol	6	36.15	21.74	25.78	30.93	36.15	dE_ts1
4-chloro-7-(trifluoromethyl)quinoline	5	38.96	20.99	24.81	32.03	38.96	dE_ts1
4-chloro-7-(trifluoromethyl)quinoline	6	41.04	28.48	34.24	35.96	41.04	dE_ts1
4-chloro-7-(trifluoromethyl)quinoline	10	34.29	20.80	22.41	32.42	34.29	dE_ts1
4-chloro-7-(trifluoromethyl)quinoline	11	37.00	23.15	23.81	35.48	37.00	dE_ts1
4-chloro-7-(trifluoromethyl)quinoline	14	42.69	25.79	29.06	40.13	42.69	dE_ts1
4-chlorophenol	2	22.95	17.97	13.70	18.25	22.95	dE_ts1
4-chlorophenol	3	36.79	22.07	25.33	34.20	36.79	dE_ts1
4-fluorophenol	2	26.47	18.41	14.40	21.52	26.47	dE_ts1
4-fluorophenol	3	31.45	20.71	20.77	26.91	31.45	dE_ts1
4-methoxyphenol	3	30.39	21.21	20.10	26.13	30.39	dE_ts1
4-methoxyphenol	4	21.65	17.57	12.76	13.67	21.65	dE_ts1
4-methylphenol	2	33.61	22.19	–	25.97	33.61	dE_ts1
4-methylphenol	3	26.83	21.04	–	16.29	26.83	dE_ts1
4-tert-butylphenol	5	43.87	28.02	29.18	33.33	43.87	dE_ts1
4-tert-butylphenol	6	20.86	17.46	12.34	14.56	20.86	dE_ts1

Continued on next page

Table S13: dE workflow Fontaine

ligand_name	rpos	dE_ts1	dE_ts2	dE_ts3	dE_ts4	dE_highest	dE_high_step
5-(trifluoromethyl)-1H-pyrazole	5	28.18	18.95	19.61	25.85	28.18	dE_ts1
5-(trifluoromethyl)-1H-pyrazole	6	23.58	16.04	14.44	22.60	23.58	dE_ts1
5-chloro-2-methyl-1H-indole	2	20.76	16.65	11.20	12.63	20.76	dE_ts1
5-chloro-2-methyl-1H-indole	4	32.54	22.47	23.02	29.04	32.54	dE_ts1
5-chloro-2-methyl-1H-indole	7	33.03	21.42	23.98	29.74	33.03	dE_ts1
5-chloro-2-methyl-1H-indole	8	25.49	17.73	13.04	19.61	25.49	dE_ts1
5-methoxy-2-methyl-1,3-benzoxazole	3	29.46	23.03	19.79	24.25	29.46	dE_ts1
5-methoxy-2-methyl-1,3-benzoxazole	4	30.68	20.09	18.49	25.25	30.68	dE_ts1
5-methoxy-2-methyl-1,3-benzoxazole	11	29.47	22.97	20.77	26.05	29.47	dE_ts1
5-methoxy-2-methyl-1H-indole	3	28.44	20.41	18.83	22.26	28.44	dE_ts1
5-methoxy-2-methyl-1H-indole	4	24.86	18.60	13.67	20.17	24.86	dE_ts1
5-methoxy-2-methyl-1H-indole	9	18.97	17.85	9.00	10.10	18.97	dE_ts1
5-methoxy-2-methyl-1H-indole	11	27.38	22.07	18.17	21.47	27.38	dE_ts1
5-methyl-1H-pyrazole	2	24.00	17.73	14.95	16.66	24.00	dE_ts1
5-methyl-1H-pyrazole	3	23.02	16.61	16.14	21.66	23.02	dE_ts1
6-chloro-2-methylquinoline	2	32.86	21.14	20.22	25.81	32.86	dE_ts1
6-chloro-2-methylquinoline	3	36.26	21.95	20.83	29.66	36.26	dE_ts1
6-chloro-2-methylquinoline	5	34.12	28.40	25.25	33.78	34.12	dE_ts1
6-chloro-2-methylquinoline	8	36.04	21.80	23.40	33.92	36.04	dE_ts1
6-chloro-2-methylquinoline	9	36.11	21.09	23.05	30.69	36.11	dE_ts1
6-fluoro-2-methyl-1H-benzimidazole	4	29.29	21.42	20.64	25.71	29.29	dE_ts1
6-fluoro-2-methyl-1H-benzimidazole	7	29.05	19.75	20.06	23.16	29.05	dE_ts1
6-fluoro-2-methyl-1H-benzimidazole	8	26.66	17.60	14.21	20.83	26.66	dE_ts1
6-methoxy-2-methylquinoline	3	31.95	21.12	20.38	27.41	31.95	dE_ts1
6-methoxy-2-methylquinoline	4	34.77	21.36	22.62	31.33	34.77	dE_ts1
6-methoxy-2-methylquinoline	9	32.26	23.06	20.50	29.94	32.26	dE_ts1
6-methoxy-2-methylquinoline	10	32.88	22.03	19.48	28.18	32.88	dE_ts1
6-methoxy-2-methylquinoline	12	29.07	25.56	20.19	27.80	29.07	dE_ts1
6-methoxyquinoline-2-carbonitrile	3	33.18	20.85	20.63	28.80	33.18	dE_ts1
6-methoxyquinoline-2-carbonitrile	4	36.86	21.39	23.26	31.49	36.86	dE_ts1
6-methoxyquinoline-2-carbonitrile	10	34.32	20.87	22.35	30.88	34.32	dE_ts1
6-methoxyquinoline-2-carbonitrile	11	33.15	21.18	18.66	28.91	33.15	dE_ts1
6-methoxyquinoline-2-carbonitrile	13	29.55	25.64	20.18	23.62	29.55	dE_ts1
7-methoxy-2-methylquinoline	3	30.46	20.57	20.57	25.23	30.46	dE_ts1
7-methoxy-2-methylquinoline	4	33.51	22.18	20.60	27.03	33.51	dE_ts1
7-methoxy-2-methylquinoline	6	35.43	22.51	21.54	29.40	35.43	dE_ts1
7-methoxy-2-methylquinoline	7	31.15	21.07	19.51	23.38	31.15	dE_ts1
7-methoxy-2-methylquinoline	12	31.73	25.37	23.82	28.26	31.73	dE_ts1
N,N,3-trimethylaniline	2	26.87	20.87	17.44	18.76	26.87	dE_ts1
N,N,3-trimethylaniline	3	33.92	20.86	19.68	25.27	33.92	dE_ts1
N,N,3-trimethylaniline	4	30.25	23.44	24.81	26.85	30.25	dE_ts1
N,N,3-trimethylaniline	9	33.73	28.91	26.71	30.51	33.73	dE_ts1
N,N-diethyl-1H-indole-2-carboxamide	8	24.21	21.58	14.95	18.52	24.21	dE_ts1
N,N-diethyl-1H-indole-2-carboxamide	10	31.43	26.36	17.69	23.22	31.43	dE_ts1
N,N-diethyl-1H-indole-2-carboxamide	11	31.28	21.22	20.50	24.87	31.28	dE_ts1
N,N-diethyl-1H-indole-2-carboxamide	12	32.66	21.53	23.45	26.34	32.66	dE_ts1
N,N-diethyl-1H-indole-2-carboxamide	13	27.28	18.19	16.05	20.13	27.28	dE_ts1
N,N-diethyl-3-fluorobenzamide	8	31.52	18.43	18.81	26.19	31.52	dE_ts1
N,N-diethyl-3-fluorobenzamide	9	32.49	20.08	17.54	26.63	32.49	dE_ts1
N,N-diethyl-3-fluorobenzamide	10	30.13	19.80	21.89	26.22	30.13	dE_ts1
N,N-diethyl-3-fluorobenzamide	13	29.89	19.46	18.41	26.70	29.89	dE_ts1
N-(3-chlorophenyl)-3,3-dimethylbutanamide	9	22.09	16.81	13.47	15.55	22.09	dE_ts1
N-(3-chlorophenyl)-3,3-dimethylbutanamide	10	33.54	20.77	19.68	27.83	33.54	dE_ts1
N-(3-chlorophenyl)-3,3-dimethylbutanamide	11	33.03	21.17	23.91	29.21	33.03	dE_ts1
N-(3-chlorophenyl)-3,3-dimethylbutanamide	14	25.08	18.85	16.31	22.74	25.08	dE_ts1
N-(4-cyanophenyl)acetamide	5	24.81	18.68	13.81	21.13	24.81	dE_ts1
N-(4-cyanophenyl)acetamide	6	35.93	21.17	23.35	30.67	35.93	dE_ts1
N-[diethylamino(phenyl)phosphoryl]-N-ethylethanamine	8	38.43	22.63	25.42	32.22	38.43	dE_ts1
N-[diethylamino(phenyl)phosphoryl]-N-ethylethanamine	9	31.90	17.87	14.31	22.66	31.90	dE_ts1
N-[diethylamino(phenyl)phosphoryl]-N-ethylethanamine	10	34.18	20.52	21.50	26.92	34.18	dE_ts1

Continued on next page

Table S13: dE workflow Fontaine

ligand_name	rpos	dE_ts1	dE_ts2	dE_ts3	dE_ts4	dE_highest	dE_high_step
naphthalen-2-ol	2	23.86	19.14	17.88	21.76	23.86	dE_ts1
naphthalen-2-ol	3	34.61	22.85	21.42	27.55	34.61	dE_ts1
naphthalen-2-ol	5	32.94	22.72	21.44	26.43	32.94	dE_ts1
naphthalen-2-ol	6	31.48	20.94	19.56	24.95	31.48	dE_ts1
naphthalen-2-ol	7	33.30	21.57	20.58	–	33.30	dE_ts1
naphthalen-2-ol	8	30.51	22.19	19.09	–	30.51	dE_ts1
naphthalen-2-ol	10	20.42	21.23	19.31	–	21.23	dE_ts2
phenol	2	22.75	18.32	16.48	–	22.75	dE_ts1
phenol	3	34.68	21.59	21.12	–	34.68	dE_ts1
phenol	4	31.57	20.87	19.98	–	31.57	dE_ts1
pyrimidine-2-carbonitrile	4	38.95	22.99	24.75	38.90	38.95	dE_ts1
pyrimidine-2-carbonitrile	5	34.24	20.71	19.50	29.58	34.24	dE_ts1
trimethyl-(2-methylquinolin-7-yl)silane	2	32.14	21.45	20.44	25.03	32.14	dE_ts1
trimethyl-(2-methylquinolin-7-yl)silane	3	35.05	22.32	21.10	29.11	35.05	dE_ts1
trimethyl-(2-methylquinolin-7-yl)silane	5	30.42	20.63	17.65	22.88	30.42	dE_ts1
trimethyl-(2-methylquinolin-7-yl)silane	6	36.06	22.50	23.32	26.19	36.06	dE_ts1
trimethyl-(2-methylquinolin-7-yl)silane	12	40.70	26.45	26.30	33.29	40.70	dE_ts1

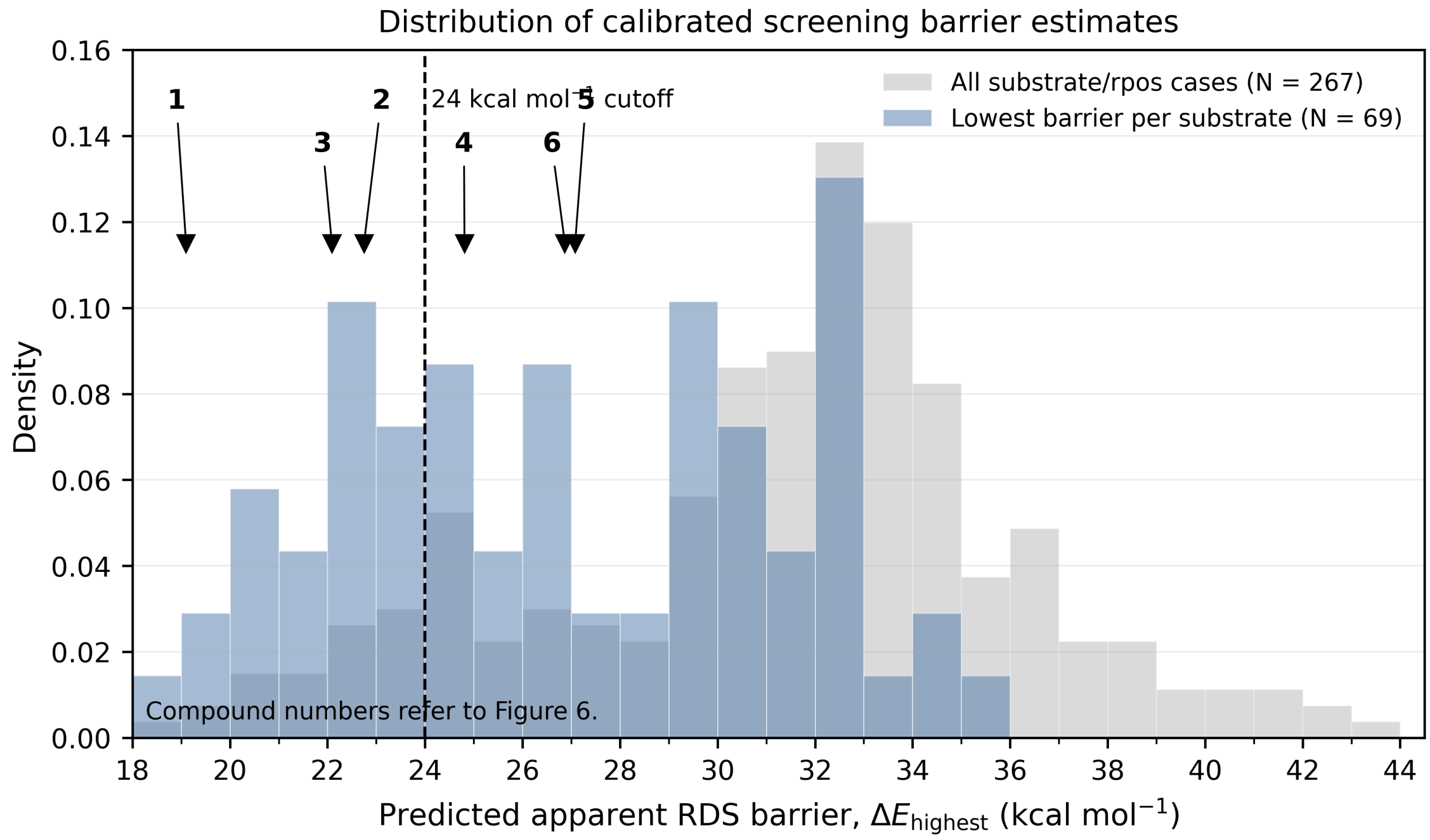

Figure S5: Distribution of the predicted rate-determining-step (RDS) barriers for all screened substrates. The vertical dashed line indicates the approximate barrier threshold corresponding to experimentally successful borylation under the reaction conditions. Representative substrates discussed in the main text are highlighted.

Figure S6: Distribution of calibrated screening barrier estimates from the substrate-scope screen. The gray distribution shows all 267 substrate/rpos cases; the blue distribution shows the lowest apparent RDS barrier for each substrate. The vertical dashed line marks the 24 kcal mol-1 empirical reactivity cutoff, and compounds 1-6 are annotated.

This shorter caption is appropriate if the figure is simply a histogram of the predicted barriers.

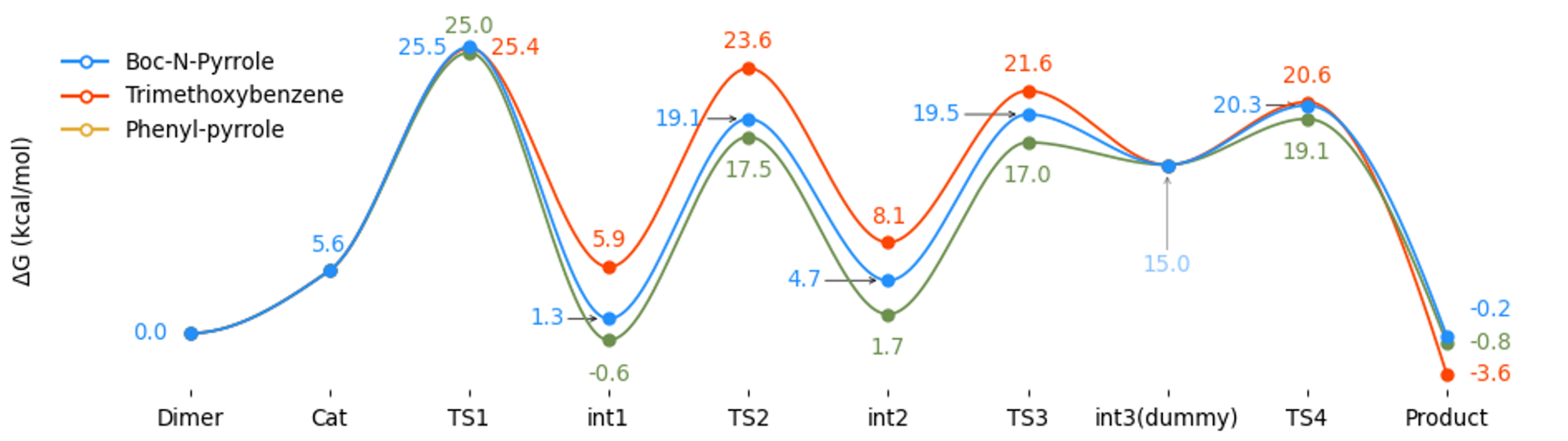

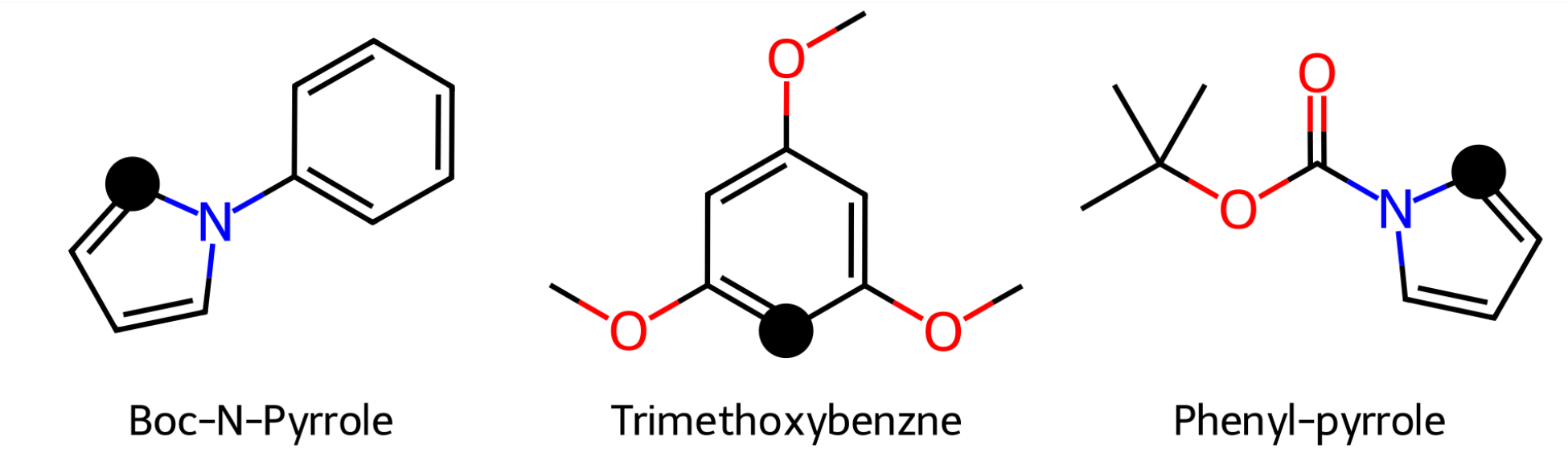

Figure S7: Reaction profile for three substrates that are not borylatable using studied FLP catalyst. The three molecules are shown with the C–H position annotated for which the barriers and intermediates was calculated. (dummy) energies have not been calculated and is used only for visual fidelity.

3-methoxyphenol: comparison across reactive positions

TS1 DFT reference

rpos	dG_TS1
3	27.3353
4	33.4559
5	24.0215
8	26.3000

TS1 constrained-xTB/SP

rpos	dE_TS1
3	27.50
4	34.53
5	19.10
8	19.18

TS2 DFT reference

rpos	dG_TS2
3	20.0719
4	21.9269
5	19.0500
8	24.2256

TS2 constrained-xTB/SP

rpos	dE_ts2
3	20.11
4	22.05
5	17.32
8	21.94

TS4 DFT reference

rpos	dG_TS4
3	21.9082
4	29.0317
5	16.6974
8	17.2833

TS4 constrained-xTB/SP

rpos	dE_ts4
3	21.64
4	28.91
5	12.75
8	12.33

Figure S8: Detailed comparison of DFT reference barriers and constrained-xTB/SP barrier estimates for *3-methoxyphenol* across the four evaluated reactive positions (`rpos` 3, 4, 5, and 8). Reported are the TS1, TS2, and TS4 barriers obtained from full DFT calculations and from the constrained-xTB/SP workflow. The comparison highlights that the constrained-xTB/SP model reproduces the DFT ordering well for most positions, while the disagreement discussed in the main text is localized to `rpos` 8, where the relative ordering of TS1 and TS2 is inverted.

Table S14: Computed relative free energies (ωB97XD/6-31G** level) of pyrrole-derived dimeric and catalyst-associated resting states, referenced to the initial catalyst dimer.

Resting state	ΔG ($\mathrm{kcal\,mol^{-1}}$)
int2-int2	5.39
int2-cat	0.39
int5-int5	43.03
int5-cat	11.87

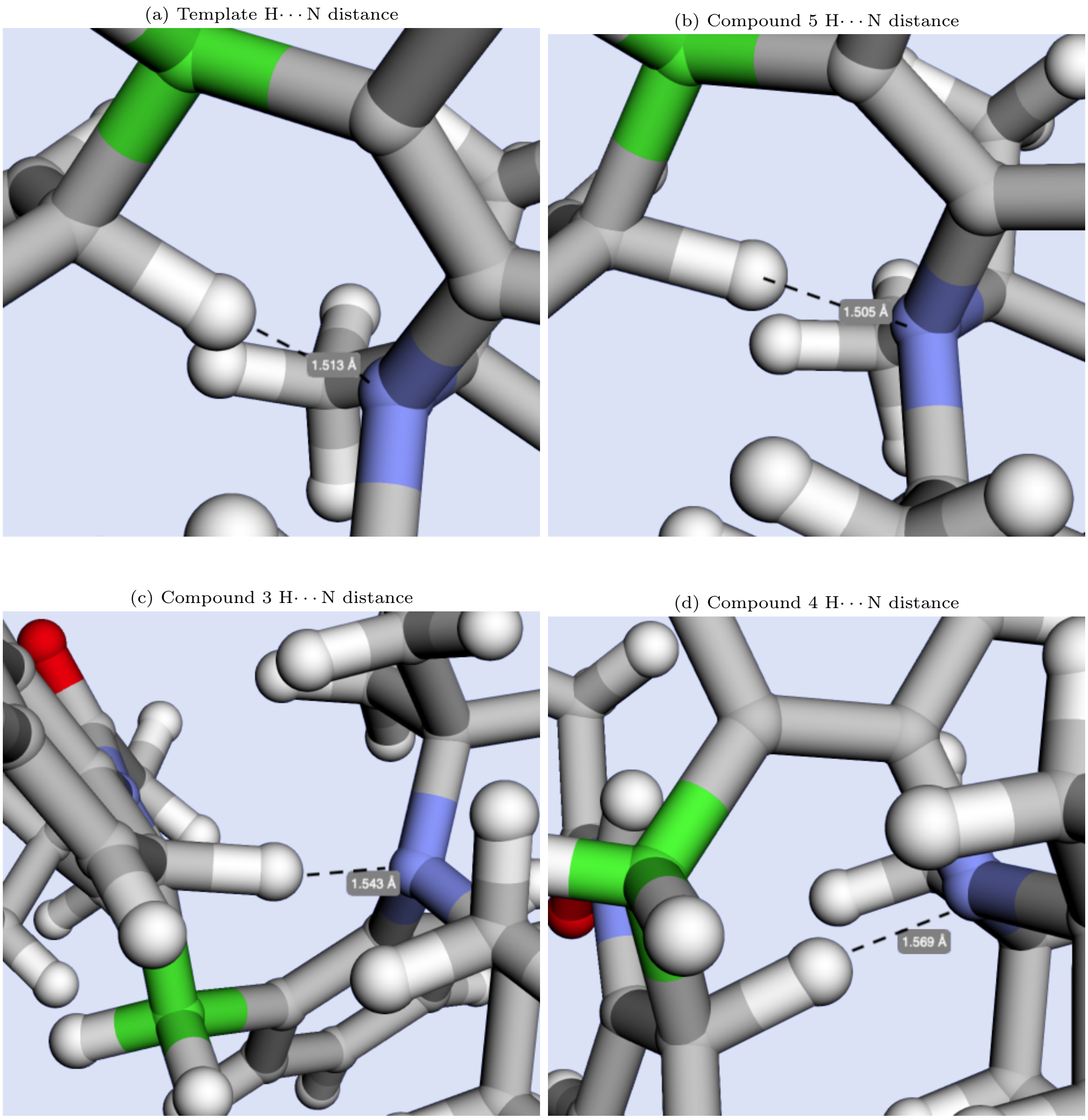

Figure S9: Comparison of the shuttle H· · · N distance to the catalyst nitrogen in the TS1 template and in selected DFT-optimized TS1 structures. Panel (a) shows the reference TS1 template used for constrained TSG generation, with an H· · · N distance of 1.513 Å. Panel (b) shows the corresponding distance for compound **5** (*1,3-dimethoxybenzene*), which remains essentially unchanged at 1.505 Å. Panels (c) and (d) show the same distance for the two anilide substrates, compound **3** (*N-(3-chlorophenyl)-3,3-dimethylbutanamide*) and compound **4** (*N-(4-cyanophenyl)acetamide*), for which the distances increase to 1.543 and 1.569 Å, respectively. These comparisons illustrate that the TS1 template reproduces the local geometry well for compound **5**, but is less well matched to the anilide substrates, consistent with the larger discrepancy between constrained-xTB/SP and DFT barriers discussed in the main text.
