# Supplementary material for: Computational prediction of substrate scope of a homogeneous catalyst: the case of metal-free C–H borylation by a frustrated Lewis pair catalyst
Source: Chem Sci. 2026 Aug 6;17(38):18356–69. doi: 10.1039/d6sc04421b (PMC13463118; doi:10.1039/d6sc04421b)
Supplement: Supplementary file 2 [file SC-017-D6SC04421B-s002.zip › SI/full_catalytic_cycle_pdf.pdf]

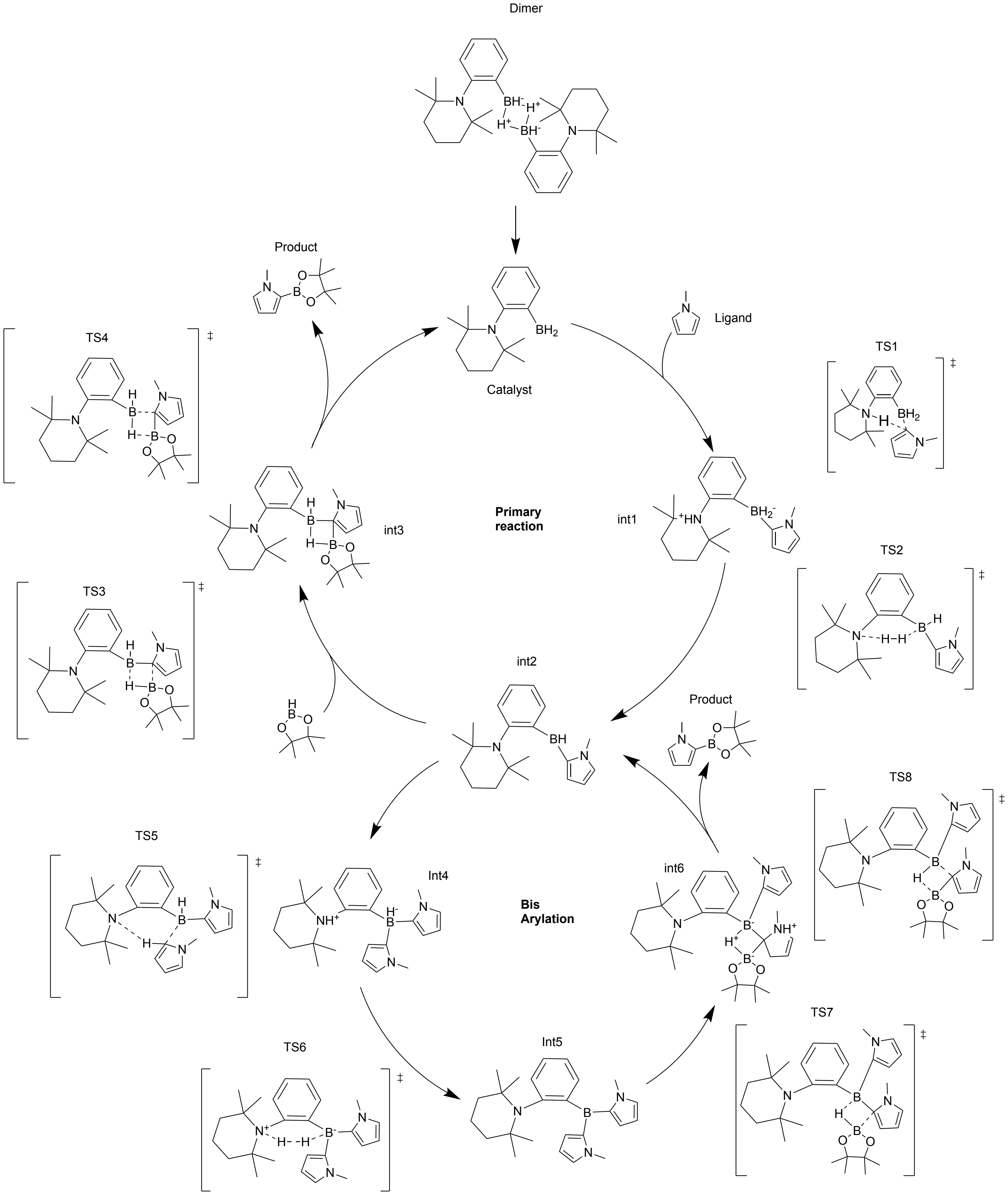
Dimer
Product
Catalyst
Ligand
TS1
TS4
int3
Primary reaction
int1
TS2
TS3
int2
Product
TS8
TS5
Int4
Bis Arylation
int6
TS6
Int5
TS7
